# Supplementary material for: Adjunctive Varespladib after antivenom administration for snakebite: A phase II randomized clinical trial
Source: PLoS Negl Trop Dis. 2026 Sep 21;20(9):e0014736. doi: 10.1371/journal.pntd.0014736 (PMC13614639; doi:10.1371/journal.pntd.0014736)
Supplement: Supplementary file 1 [file pntd.0014736.s002.pdf]

PROTOCOL

PRODUCT NAME:	Varespladib (intravenous) and varespladib-methyl (oral)
PROTOCOL NUMBER:	OPX-PR-03
IND NUMBER:	142870
NCT NUMBER:	05717062
CTRI NUMBER:	2023/10/058782
DEVELOPMENT PHASE:	Phase 2
PROTOCOL TITLE:	Randomized, Double-Blinded, Placebo-Controlled Study to Evaluate the Safety, Tolerability, and Efficacy of Intravenous Varespladib Followed by Oral Varespladib in Addition to Standard of Care in Subjects Bitten by Venomous Snakes<br>(**BRAVIO** - **B**road-spectrum **R**apid **A**ntidote: **V**arespladib **I**V to **O**ral Trial for snakebite)
PROTOCOL DATE:	Version 6.0, 21 October 2024
SPONSORED BY:	Ophirex, Inc.<br>5643 Paradise Drive #2<br>Corte Madera, CA 94925<br>(415) 891-8682
CONTRACT RESEARCH ORGANIZATION:	Syneos Health

This study will be performed in compliance with International Council for Harmonisation (ICH) Good Clinical Practices and applicable regulatory requirements, including the archiving of essential documents. Information contained in this protocol is confidential in nature, and may not be used, divulged, published, or otherwise disclosed to others except to the extent necessary to obtain approval of the institutional review board or independent ethics committee, or as required by law. Persons to whom this information is disclosed should be informed that it is confidential and may not be further disclosed without the express permission of Ophirex, Inc.

1 PROTOCOL SUMMARY

1.1 Synopsis

**Number of Subjects**	Approximately 140 subjects enrolled
**Population**	Subjects ≥ 5 years of age
**Investigational Product**	Varespladib (LY315920), intravenous form, and varespladib-methyl (LY333013), oral form
**Study Objectives**	
Primary Efficacy	To evaluate the efficacy of intravenous (IV) followed by oral varespladib in addition to standard of care (SOC) in subjects bitten by venomous snakes.
**Study Endpoints**	
Primary Efficacy	For subjects bitten by elapids: Time to complete head-lift recovery defined by a 5-second head-lift of 5 second.<br>For subjects bitten by vipers: Area Under the Curve (AUC) of an abbreviated Snakebite Severity Score (SSS) composed of local wound, hematologic, and neurologic subscores from Baseline (pre-dosing) to Day 14.<br>The independent p-values from subjects bitten by elapids and subjects bitten by vipers will be analyzed using Fisher's combined probability test to obtain a single global p-value for testing the primary endpoint.
Secondary Efficacy	• Area under the curve of the composite outcome of the pulmonary, cardiovascular, local wound, hematologic, renal, and nervous system sections of the snakebite severity score (SSS) from Baseline (pre-dosing) to Day 7 for all patients and for patients randomized in ≤5 hours from bite.<br>• Patient Specific Functional Scale (PSFS) score at Day 3 and Day 7.<br>• Complete snakebite severity score (SSS) recovery, defined as a composite score of 0 for the pulmonary, cardiovascular, local wound, hematologic, renal, and nervous system sections of the SSS at Day 28.<br>• Total antivenom requirement from Baseline through Day 28<br>• Duration of hospitalization from Baseline through Day 28
Exploratory Efficacy	• Change in the composite outcome of the 6-item SSS (pulmonary, cardiovascular, local wound, hematologic, renal, and nervous system subscores) from Baseline through Day 28<br>• Change in the 6-item SSS from Baseline through Day 28 using compressed SSS scale<br>• Individual SSS subscores from Baseline through Day 28<br>• Focused hematologic score based on INR and platelets from Baseline through Day 28<br>• Coagulation abnormalities (separately PT, PTT, platelets, and fibrinogen) from Baseline through Day 28<br>• Change in NPRS from Baseline through Day 28

	• Clinical Global Impression-Improvement (CGI-I) from Baseline through Day 28<br>• Patient Global Impression of Change (PGIC) from Baseline through Day 28<br>• Patient-Specific Functional Scale (PSFS) average score from Baseline through Day 28<br>• Levels of the myonecrosis marker, creatine kinase (CK), from Baseline through Day 3<br>• For patients bitten by elapids, change in 5-second head-lift duration from Baseline through Day 28<br>• For patients bitten by elapids, grip strength from Baseline through Day 28<br>• For patients bitten by elapids, duration of ventilatory support from Baseline through Day 28<br>• Analgesic use from Baseline through Day 28<br>• Transfusion requirement from Baseline (Day 1) through Day 28<br>• Elapsed time from initiation of IV varespladib treatment until the transition to oral varespladib-methyl<br>• All cause mortality through Day 28<br>• Secretory phospholipase A2 (sPLA2) from Baseline through Day 7
Pharmacokinetics	PK parameters of varespladib in plasma from all study subjects using the following schedule. PK analysis will be conducted using population PK modeling methods.

IV	Oral 1st dose	Oral 2nd dose	Day 7 (Oral)
• Predose<br>• 0.5h post initiation<br>• End of infusion	• 2 hours after first oral dose<br>• Either 5-10 hours after first oral dose OR after 2nd, 3rd, or 4th in-hospital oral dose	**If Hospitalized:**<br>Sample prior to second oral dose<br>OR<br>**If Discharged prior to second dose**:<br>Sample prior to discharge	• Predose

Safety	• Incidence and severity of adverse events (AEs), serious AEs (SAEs), and AEs leading to discontinuation of Investigational Product (IP)<br>• Safety of varespladib and varespladib-methyl as assessed by the number and rates of reported treatment-emergent adverse events (TEAEs) from beginning of treatment until last Follow-Up Visit/Telephone Call at Day 28<br>• Number of subjects with a treatment-related SAE from beginning of treatment until last Follow-Up Visit/Telephone Call at Day 28<br>• Safety as assessed by<br>– Vital signs<br>– Clinical laboratory evaluations: CBC, urinalysis, liver function tests [LFTs], renal function tests (creatinine, blood urea nitrogen [BUN], estimated glomerular filtration rate [eGFR])<br>– 12-lead electrocardiogram (ECG)<br>• Concomitant medications and therapies<br>• Columbia-Suicide Severity Rating Scale (C-SSRS) evaluated at Baseline or at the earliest time point clinically allowable (ideally Day 1) and then at every study visit through Day 28
**Investigational Product**	<u>Varespladib</u><br>Sterile Injectable: Varespladib (LY315920) formulated as lyophilized powder in a single-use vial containing 100 mg of LY315920 for reconstitution with Water for Injection (WFI), followed by dilution into 0.9% Sodium Chloride.<br><br><u>Varespladib-methyl</u><br>Tablet: Varespladib-methyl (LY333013) formulated as an immediate-release, film-coated oval tablet (250 mg).<br>Capsule: Varespladib-methyl (LY333013) formulated as an immediate-release capsule (50 mg).
**Control Article**	<u>Placebo</u><br>IV: Intravenous saline (0.9%).<br>Tablet: The oral placebo tablet is supplied as a film-coated oval tablet to match the LY333013 250 mg tablet for oral administration and contains only the excipients: lactose monohydrate, microcrystalline cellulose, and magnesium stearate.<br>Capsule: The oral placebo capsule is supplied as an immediate-release capsule to match the LY333013 50 mg capsule for oral administration and contains only the excipients: lactose monohydrate, microcrystalline cellulose, and magnesium stearate.
**Route of Administration**	Intravenous (varespladib) followed by oral (varespladib-methyl)
**Dose and Administration**	Following randomization, study subjects will receive a continuous rate infusion of intravenous investigational product (IP) or placebo for 6 hours. (The age-based dosing of the intravenous and oral drug are provided in the table below.) At 6 hours, the subject will be assessed for transition to oral IP. If the subject meets clinical criteria, the infusion will be stopped, and the oral IP will be initiated. Recommended clinical criteria for stopping the

<table>
<tr><td></td><td>
infusion and initiating oral IP include the following:

(1) SSS Nervous System Score ≤ 1;
(2) clinically stable as judged by the treating physician;
(3) able to tolerate oral intake.

If the subject does not meet clinical criteria, the infusion will continue. Thereafter, the subject will be reassessed at least twice a day, and transitioned to the oral IP when they meet clinical criteria. The maximum duration of IV study drug is 48 hours, at which point all patients will switch to the oral drug, unless a patient is intubated and is unable to receive medication via nasogastric tube. Regardless of when the subject transitions from intravenous to oral therapy, the total duration of therapy from initiation of intravenous IP for all patients will be 7 days.

When the subject transitions to the oral IP, subjects will be given an initial loading dose of oral varespladib-methyl based on their age. Thereafter, subjects will take oral varespladib-methyl every 12 hours (q12h) for a total duration of 7 days from the initiation of the infusion.

Oral treatment will be initiated immediately (within ≤15 minutes) after the cessation of the infusion.

<table>
<tr><th>Formulation, Dose</th><th>Age 5 to 11 years</th><th>Age ≥12 and <18 years</th><th>Age ≥18 years</th></tr>
<tr><td>Intravenous</td><td>0.6 mg/kg/hr</td><td>0.6 mg/kg/hr</td><td>0.45 mg/kg/hr</td></tr>
<tr><td>Oral, first dose</td><td>200 mg (4 x 50 mg capsules)</td><td>400 mg (8 x 50 mg capsules)</td><td>500 mg (2 x 250 mg tablets)</td></tr>
<tr><td>Oral, all doses after the first dose</td><td>100 mg (2 x 50 mg capsules)</td><td>200 mg (4 x 50 mg capsules)</td><td>250 mg (1 x 250 mg tablet)</td></tr>
</table>
</td></tr>
<tr><td>Follow-Up</td><td>Subjects will be assessed on a regular schedule for a total of 28 days after initiating treatment with varespladib.</td></tr>
<tr><td>Eligibility</td><td>To be eligible to participate in the study, an individual must meet all listed inclusion criteria and none of the exclusion criteria.</td></tr>
</table>

Inclusion Criteria	1. Is a male or female ≥ 5 years of age.<br>2. Patients must have known or suspected venomous snakebite. In India, enrollment will be restricted to patients bitten by suspected or confirmed Russell's viper (*Daboia russelii*) or krait (*Bungarus spp.*). In the U.S., any snakebite that meets all other criteria may be eligible.<br>3. Patients must meet one of two categories of inclusion criteria:<br>**Category 1**: The patient is enrolled within 5 hours of venomous snake bite or symptom onset with an SSS score* of ≥2 in one system and ≥1 in another system (2+1).<br>OR<br>**Category 2**: The patient has a suspected or confirmed bite from an elapid and is enrolled within 10 hours of bite or symptom onset with moderate to severe cranial nerve or skeletal muscle weakness**.<br>4. Is willing (or legally authorized representative is willing) to provide informed consent prior to initiation of any study procedures.<br>* Only local wound, pulmonary, cardiovascular, hematologic, or nervous system scores qualify for SSS inclusion criteria. Hematologic score may be used if available, but inclusion should not wait for laboratory results. Point of care tests (e.g., 20WBCT) may be used for enrollment, if used per site standard of care (see Appendix A). GI and Renal scores are not used for inclusion.<br>** Isolated ptosis does not meet the definition of moderate to severe neurotoxicity for enrollment
Exclusion Criteria	1. Has history of or is suspected to have cerebrovascular accident or intracranial bleeding of any kind, acute coronary syndrome, myocardial infarction, or severe pulmonary hypertension.<br>2. Has known history of inherited bleeding or coagulation disorder.<br>3. Is, at Screening Visit, using the following anticoagulants: warfarin/coumadin, argatroban, bilvalirudin, lepirudin, apixaban, dabigatran, clopidogrel, prasugrel, ticlodipine or another anticoagulant agent not specifically listed, or has used heparin, enoxaparin, fondaparinux, or other low molecular weight heparin or any antiarrhythmic drugs within 14 days prior to treatment.<br>4. Has a history of chronic liver disease such as chronic active viral hepatitis, alcohol-related liver disease, non-alcoholic steatohepatitis, non-alcoholic fatty liver disease, hemochromatosis, primary biliary cirrhosis, primary sclerosing cholangitis, autoimmune hepatitis.<br>5. Reports or has known pre-existing renal impairment or chronic kidney disease.<br>6. Has a known allergy or significant adverse reaction to varespladib or varespladib-methyl.

	7. Is considered by the Investigator to be unable to comply with protocol requirements due to geographic considerations, psychiatric disorders, or other compliance concerns.<br>8. Is pregnant, has a positive urine or serum human chorionic gonadotropin (hCG) pregnancy test or not willing to use a highly effective method of contraception for 14 days after initial treatment, or is breast-feeding.
**Safety Follow-up Assessments**	Follow-up schedule: Day 3, Day 7, Day 14 and Day 28 after initiation of varespladib. Following hospital discharge these follow-ups will still occur in person as arranged by site.
**Study Duration**	Approximately 18 months
**Sample Size**	The total enrollment for this study will be approximately 140 subjects. The study will have approximately 90% power.<br><br>The assumptions used to calculate power are as follows:<br>• Two-sided alpha level 5% for each of the two tests (elapids and vipers).<br>• Randomization Ratio 1:1. The randomization will be stratified by snake type: copperhead, Mojave rattlesnake, non-Mojave rattlesnake, Russell's viper, Elapid, or other, and by age group: pediatrics aged 5 to 17 years or adults aged 18 years and older.<br>• The power for the elapid analysis will be greater than 90%. To determine power, we assumed 44 participants with elapid bites and calculated a mean difference in time to recovery between the treatment groups based on expected outcomes. The mean difference based on expected outcomes was then compared to the distribution of mean differences for 200 cases in which varespladib and placebo were randomly assigned in a 1:1 ratio (22 for each treatment group) to the expected outcome data.<br>• The power for the viper analysis will be approximately 77%. This calculation assumes the study will enroll at least 120 viper-bite patients and a difference in the relevant outcome (composite local wound, hematology, and neurologic subscores of the SSS AUC) from Baseline to Day 14 of 125 points with a standard deviation of 225. Further, the sample size assumes that roughly 10% of randomized viper-bite patients will fail to complete the study and that those will be assigned imputed values as if they had been assigned to placebo.
**Statistical Analysis**	Descriptive statistics (n, mean, median, standard deviation [SD], minimum, and maximum for continuous data; frequencies and percentages for categorical data) will be used to summarize study data and details of analysis are outlined in the study Statistical Analysis Plan.
Efficacy	All statistical testing for efficacy endpoints will be 2-sided and performed using a significance level of 0.05. All efficacy analyses will be performed on the analysis populations using descriptive statistics.

Safety	Safety and tolerability will be evaluated by examining the occurrence of AEs, including TEAEs. AEs leading to discontinuation from the study drug, causality and severity will be summarized by treatment group. AEs will also be presented in listings. Summary statistics of clinical laboratory measures, vital signs, and ECG will be presented by treatment group for each assessment. Number and percentage of subjects presenting at least one post-Baseline potentially clinically significant abnormality will be presented by treatment group for selected parameters. All safety analyses will be performed on the Safety Population set using descriptive statistics.

1.2 Schedule of Events

Table 1 Schedule of Events

**Visit[a]**	**Baseline (Day 1)**	**Dosing (Day 1)**	**Day 1[c]**			**Day 2[d]**	**Day 3**	**Day 7**	**Day 14**	**Day 28 EOS Visit**
**Time After Dosing[e]**	**Pre-dosing**	**0h**	**0.5−1h**	**3−4h**	**5−6h**	**2d**	**3d**	**7d ± 1d**	**14d ± 2d**	**28d ± 3d**
Abbreviated consent (optional)[f]	X									
Informed consent	X									
Review inclusion / exclusion criteria	X									
Demographics	X[b]									
Medical history, including details of envenoming and treatments/interventions including antivenom receipt prior to enrollment	X									
Body height/weight per institutional protocol	X[b]									X
Physical examination	X					X	X	X	X	X
Vital signs[g]	X			X	X	X	X	X	X	X
Randomization and IP assignment	X									
Snakebite Severity Score (SSS) assessments:[h]<br><br>Individual Category Severity Assessments: Pulmonary, Cardiovascular, Local Wound, Gastrointestinal, Hematologic, Renal, Nervous Systems	X			X[g]	X[g]	X	X	X	X	X
Head-Lift duration in inpatient participants (0 to 5 seconds)[i]	X			X	X	X	X	X	X	X
Numeric Pain Rating Scale (NPRS)	X			X	X	X	X	X	X	X
12-lead ECG	X[b]				X		X	X		X
Grip strength[j]	X			X	X	X	X	X	X	X
Laboratory assessments (see individual Laboratory Assessments Schedule of Events below)[k]	X			X	X	X	X	X	X	X

Visit[a]	Baseline (Day 1)	Dosing (Day 1)	Day 1[c]			Day 2[d]	Day 3	Day 7	Day 14	Day 28 EOS Visit
**Time After Dosing[e]**	**Pre-dosing**	**0h**	**0.5−1h**	**3−4h**	**5−6h**	**2d**	**3d**	**7d ± 1d**	**14d ± 2d**	**28d ± 3d**
Patient Global Impression of Change (PGIC)[l]	X[b]			X	X	X	X	X	X	X
Clinical Global Impression-Improvement (CGI-I)[m]	X			X	X	X	X	X	X	X
Clinical Global Impression-Expected Recovery (CGI-ER)								X		
Clinical Global Impression-Severity of Illness (CGI-S) Scale	X									
Patient-specific functional scale (PSFS)	X[b]				X	X	X	X	X	X
Columbia-Suicide Severity Rating Scale (C-SSRS)[n]	X[b]					X	X	X	X	X
IP administration (including IV to Oral transition)[o]		←	—	—	—	—	→			
Standard of care (SOC)[p]	←	—	—	—	—	—	—	—	—	→
Assess and record adverse events (AEs)	←	—	—	—	—	—	—	—	—	→
Record concomitant medications/therapies/analgesic use, including sedatives and paralytics for intubated patients	←	—	—	—	—	—	—	—	—	→
Additional assessments in intubated subjects[q]	←	—	—	—	—	—	—	—	—	→
Record pulmonary support interventions[r]	←	—	—	—	—	—	—	—	—	→
Record hospitalization details	←	—	—	—	—	—	—	—	—	→
Complete blood count (including WBCs with differential, hemoglobin/hematocrit, platelets)	X			X	X	X	X	X	X	X
Serum chemistry (including sodium, potassium, chloride, bicarbonate, BUN and creatinine with estimated GFR)	X			X	X	X	X	X	X	X
Serum pregnancy	X[v]									X
Liver function tests (LFTs)[s]	X			X	X	X	X	X		X
Hemolysis markers (hemolysis measured by LDH and bilirubin)	X			X	X	X	X			
PT, PTT, thrombin time, INR	X			X	X	X	X	X	X	X

**Visit**[a]	**Baseline (Day 1)**	**Dosing (Day 1)**	**Day 1**[c]			**Day 2**[d]	**Day 3**	**Day 7**	**Day 14**	**Day 28 EOS Visit**
**Time After Dosing**[e]	**Pre-dosing**	**0h**	**0.5−1h**	**3−4h**	**5−6h**	**2d**	**3d**	**7d ± 1d**	**14d ± 2d**	**28d ± 3d**
Absolute fibrinogen	X			X	X	X	X	X	X	X
Urinalysis, general[t]	X[b]					X	X	X		X
Urine pregnancy	X[w]									
Myonecrosis marker: creatine kinase (CK)	X			X	X	X	X			
$sPLA_2$ biomarker[u,v]	X		X[s]	X[s]				X		
Pharmacokinetics sample[u,v]	X		X[s]	X[s]				X		

Abbreviations: AE = adverse event; BID = twice daily; BUN = blood urea nitrogen; CGI-I = Clinical Global Impression-Improvement; CGI-S = Clinical Global Impression-Severity of Illness; CK = creatine kinase; C- SSRS = Columbia-Suicide Severity Rating Scale; ECG = electrocardiogram; EOS = End of Study; GFR = glomerular filtration rate; INR = international normalized ratio; IP = investigational product; LDH = lactate dehydrogenase; MIP = maximum inspiratory pressure; NIF = negative inspiratory force; NPRS = numeric pain rating scale; PGIC = patient global impression of change; PK = pharmacokinetic; PSFS = patient-specific functional scale; PT = prothrombin time; PTT = partial thromboplastin time; q12h = once every 12 hours; SOC = Standard of care; $sPLA_2$ = secretory phospholipase A_2; SSS = snakebite severity score; WBC = white blood cell

a Screening, enrollment, and Day 1 of the Treatment Period will take place on the same day.

b This noncritical assessment should be performed as early as possible, but may be performed after randomization and initiation of study drug or SOC if required due to patient condition. Weight is required at Baseline (pre-dosing) for IV dose calculations.

c Assessments (besides PK blood draws for each individual subject on Day 1) should be timed such that ≥ 90 minutes elapses between measurements.

d Assessments for each individual subject between hours 5 to 6 of Day 1 and the same measurements on Day 2 (except PK blood draws) should be timed such that at least 16 and not more than 24 hours elapse between measurements.

e Assessments for each individual subject on Day 2 should be timed such that assessment occurs 16 to 24 hours after initiation of IP.

f The abbreviated consent is optional. The purpose of the abbreviated consent is to provide key information about the study to the patient in order to allow the patient to provide initial agreement regarding randomization. This agreement can be used by sites to randomize patients and request study drug, which should reduce the time to initiation of the study drug. Patients will not receive study drug until they have reviewed and signed the full consent.

g Vital signs will be assessed per institutional practice, but at a minimum of once per time point.

h For intubated patients, please collect and record a complete SSS within 6 hours of extubation.

i Head-lift will be evaluated from 0 to 5 seconds as described in Section 9.3.1.8.

j Grip strength should be performed as described in Section 9.3.1.9.

k Administration of treatment should **not** be delayed until laboratory test results are returned (e.g., liver, renal, or serum pregnancy tests).

l The PGIC score is a rapid, qualitative assessment.

m Baseline CGI-I should be in comparison to previous clinical assessment. All post-baseline CGI-I assessments should be compared to baseline CGI-I as described in Appendix C1.

[n] The C-SSRS will be evaluated at Baseline or at the earliest time point clinically allowable and then at every study visit through Day 28, with modifications to the scales at different study dates as described in Section 9.3.1.6.

[o] Continuous rate IV infusion (0.45 mg/kg/hr) will be administered from time 0 to 6 hours, at which point each subject will be assessed to determine if they meet criteria for transitioning to the oral investigational product as described in Section 6.3. If and when the subject meets clinical criteria for transition to oral drug, they will be transitioned to varespladib-methyl (initial dose 500 mg or 400/200 mg for adults or pediatric patients, respectively) then continued oral dosing q12 (250 mg or 200/100 mg for adult or pediatric patients, respectively) for the remainder of the study period. Subjects that do not meet the criteria for transitioning to the oral drug will remain on the IV infusion at 0.45 mg/kg/hr until they meet criteria for transition. For pediatric patients, the continuous rate IV infusion is 0.6 mg/kg/hr.

[p] SOC (including antivenom as required) will continue to be administered throughout the subject's participation in the study according to the protocol and the judgment of the Investigator.

[q] For endotracheally intubated subjects, perform additional assessments as described in Section 9.3.1.7.

[r] Record the type of control of mechanical ventilation as described in Section 9.3.1.7 and Appendix C8. Criteria for initiation of weaning should be assessed as described in Section 9.3.1.7.

[s] Thrombin time, direct and indirect bilirubin, and gamma-glutamyl transpeptidase (GGT) are optional tests

[t] Urinalysis should not delay care and should not be obtained by catheter unless patient already being catheterized (e.g., Russell's viper). Urine from catheterized patients should not be collected from bag, but rather proximal port.

[u] $sPLA_2$ activity and PK samples will be processed and analyzed in a central laboratory, not at individual sites.

[v] PK and sPLA2 sampling will be performed for all subjects as follows: During IV dosing: pre-initiation of infusion, 0.5 hours post initiation of IV infusion, and immediately prior to the end of IV infusion; during oral dosing: 2 hours post initiation of first oral dose (500 mg adult / 400/200 mg pediatric), 5-10 hours post first (500 mg / 400/200 mg) OR after 2nd, 3rd, or 4th oral dose (250 mg / 200/100 mg), pre-dose second oral dose (first 250 mg / 200/100 mg) OR prior to discharge if discharged before second oral dose, and Day 7 pre-dose.

[w] Pregnancy test with negative result must be completed prior to randomization. This test may be serum-based or urine-based. If urine-based test is used to support randomization, a serum test must be performed as a validation.

Figure 1 **Study Schematic**

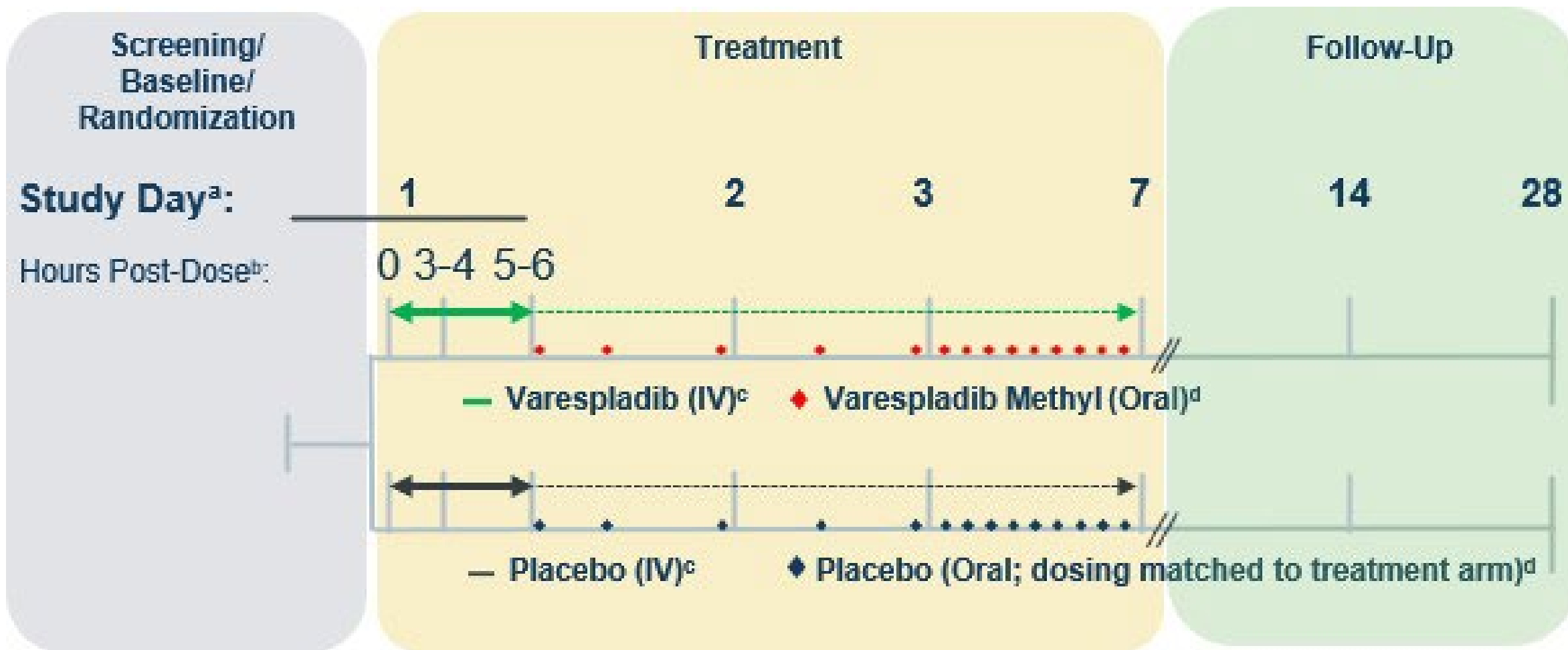

[a] Because snakebite subjects are considered critically ill and will arrive in an emergency department setting, Screening, Baseline evaluation, randomization, and treatment must all take place on Study Day 1

[b] Assessments (see Schedule of Events) must be performed at the following times post initial dose: Day 1: 3 to 4 hours, 5 to 6 hours; Day 2: 16 to 24 hours after last Day 1 measurements; Day 3: 48 hours ± 12 hours; Day 7 ± 1 day; Day 14 ± 2 days; Day 28 ± 3 days

[c] Continuous rate IV infusion (Age ≥18 years 0.45 mg/kg/hr; Age 5 to <18 years 0.6 mg/kg/hr) will be administered from time 0 to 6 hours, at which point each subject will be assessed to determine if they meet criteria for transitioning to the oral investigational product as described in Section 6.3.

[d] If and when the subject meets clinical criteria for transition to oral drug, they will be transitioned to varespladib-methyl with initial dose (Age 5 to 11 years 200 mg; Age ≥12 and <18 years 400 mg; Age ≥18 years 500 mg) then continued oral dosing q12h (Age 5 to 11 years 100 mg; Age ≥12 and <18 years 200 mg; Age ≥18 years 250 mg) for the remainder of the study period. If subjects do not meet clinical criteria for transition, IV administration will continue (dotted green line) and subjects will be reassessed at least twice per day. Solid lines show IV study drug being given for first 6 hours which is minimum requirement. All patients are expected to switch to oral drug by 48 hours unless intubated and unable to receive oral drug via nasogastric tube. Abbreviations: q12h = once every 12 hours; yr = years

2 TABLE OF CONTENTS

1 PROTOCOL SUMMARY 2
1.1 Synopsis 2
1.2 Schedule of Events 9
2 TABLE OF CONTENTS 14
2.1 List of In-text Tables 19
2.2 List of In-text Figures 19
LIST OF ABBREVIATIONS AND DEFINITION OF TERMS 20
3 INTRODUCTION 21
3.1 Background and Rationale 21
3.1.1 Scientific Rationale 22
3.2 Clinical Experience 24
3.3 Summary of Potential Risks and Intent to Benefit 24
4 OBJECTIVES AND ENDPOINTS 28
5 STUDY DESIGN 30
5.1 Overall Study Design and Plan 30
5.2 Rationale and Discussion of Study Design 31
5.3 Selection of Doses in the Study 32
5.3.1 Study Design Rationale 32
5.3.2 Dose Justification 34
5.4 Study Sites 35
5.5 End of Study Definition 36
6 STUDY POPULATION 36
6.1 Selection of Study Population 36
6.2 Study Entry Criteria 36
6.2.1 Inclusion Criteria 36
6.2.2 Exclusion Criteria 37
6.3 Early Subject Withdrawal 37
6.4 Discontinuation of Study Intervention 38
6.5 Subject Replacement Criteria 38
7 TREATMENTS 38
7.1 Identification of Investigational Products or Placebo 38
7.2 Selection of Timing of Dose for Each Subject 39
7.3 Dose Adjustment Criteria 40
7.4 Treatment Compliance 40
7.5 Method of Assigning Subjects to Treatment Groups 40
7.6 Blinding and Unblinding Treatment Assignment 40

7.7 Prohibited and Permitted Therapies 41
7.7.1 Prohibited Therapies 41
7.7.2 Permitted Therapies 41
7.8 Treatment After End of Study 41
7.9 Dispensing and Storage 42
7.10 Drug Accountability 42
7.11 Labeling and Packaging 42
7.11.1 Labeling 43
8 STUDY PROCEDURES 43
8.1 Study Duration 43
8.2 Study Periods and Visits 43
8.2.1 sPLA2 and Pharmacokinetics Sample Timing 43
8.2.2 Screening (Enrollment and Baseline) 44
8.2.3 Treatment Period 46
8.2.3.1 Day 1 (Dosing, 0 Hours) 46
8.2.3.2 Day 1 (0.5 to 1 Hour) 47
8.2.3.3 Day 1 (3 to 4 Hours) 47
8.2.3.4 Day 1 (5 to 6 Hours) 48
8.2.3.5 Day 1 (6 Hours) 49
8.2.3.6 Day 2 (18-24 hours after the "Day 1, 6 hour" assessment) 49
8.2.3.7 Day 3 (48 hours ±12 hours) 50
8.2.3.8 Day 7 (7 Days ± 1 Day) 52
8.2.3.9 Day 14 (14 Days ± 2 Days) 53
8.2.3.10 Day 28 (28 ± 3 Days) (End of Study Visit) 54
8.3 Assessments 55
8.3.1 Efficacy Assessments 55
8.3.1.1 Snakebite Severity Score 56
8.3.1.2 Clinical Global Impression-Improvement 57
8.3.1.2.1 Clinical Global Impression-Expected Recovery (CGI-ER) 57
8.3.1.3 Patient Global Impression of Change Scale (PGIC) 57
8.3.1.4 Patient-Specific Functional Scale 57
8.3.1.5 Numeric Pain Rating Scale 58
8.3.1.6 Columbia-Suicide Severity Rating Scale 58
8.3.1.7 Additional Assessments on Endotracheally Intubated Subjects 58
8.3.1.8 Head-Lift Duration 59
8.3.1.9 Grip Strength 59
8.3.1.10 Analgesic Use 59
8.3.2 Pharmacokinetics 59

8.3.2.1 Pharmacokinetic Analysis Methods 59
8.3.2.2 Pharmacokinetic Parameters 60
8.3.2.3 Sample Collection 60
8.3.3 Safety Variables 60
8.3.3.1 Clinical Laboratory Safety Assessments 61
8.3.3.2 Clinical Examinations 61
8.3.3.3 Adverse Events 62
9 ADVERSE EVENTS 62
9.1 Definitions 62
9.1.1 Adverse Events 62
9.1.2 Unexpected Adverse Event 63
9.1.3 Serious Adverse Events 63
9.1.4 Treatment-Emergent Adverse Events 64
9.2 Event Assessment and Follow-Up of Adverse Events 64
9.2.1 Assessment 64
9.2.2 Evaluation 65
9.2.2.1 Severity of Adverse Events 65
9.2.2.2 Seriousness 65
9.2.2.3 Action(s) Taken 65
9.2.2.4 Outcome at the Time of Last Observation 65
9.2.2.5 Adverse Event Relationship to Investigational Product 66
9.2.3 Documentation 66
9.2.4 Treatment of Adverse Events 66
9.2.5 Follow-Up 67
9.2.6 Reporting 67
9.2.6.1 Serious Adverse Events 67
9.3 Special Considerations 68
9.3.1 Adverse Events of Special Interest 68
9.3.2 Pregnancy 68
9.3.3 Overdosage 69
10 DATA SAFETY MONITORING BOARD 70
11 STATISTICS 70
11.1 Statistical Analysis 70
11.1.1 Analysis Populations 70
11.1.2 Study Subjects and Demographics 71
11.1.2.1 Disposition and Withdrawals 71
11.1.2.2 Protocol Deviations 71
11.1.2.3 Demographics and Other Baseline Characteristics 71

11.1.3 Exposure and Compliance 72
11.1.4 Efficacy Analysis 72
11.1.4.1 Primary Efficacy Endpoints 72
11.1.4.2 Secondary Efficacy Endpoints 72
11.1.4.3 Exploratory Endpoints 73
11.1.4.4 Primary Analysis 73
11.1.4.5 Secondary Analyses 74
11.1.4.6 Exploratory, Corroborative, Sensitivity, and Other Analyses 74
11.1.5 Clinical Pharmacology Analyses 75
11.1.5.1 Pharmacokinetics 75
11.1.6 Safety and Tolerability Analyses 75
11.1.6.1 Adverse Events 75
11.1.6.2 Clinical Laboratory Evaluations 75
11.1.6.3 Vital Signs 75
11.1.6.4 12-lead Electrocardiograms 76
11.1.6.5 Physical Examination Findings 76
11.1.6.6 Biomarkers 76
11.2 Sample Size Determination 76
12 STUDY CONDUCT 77
12.1 Sponsor and Investigator Responsibilities 77
12.1.1 Sponsor Responsibilities 77
12.1.2 Investigator Responsibilities 77
12.1.3 Confidentiality and Privacy 78
12.2 Site Initiation 78
12.3 Screen Failures 79
12.4 Study Documents 79
12.4.1 Informed Consent 79
12.4.2 Investigator's Regulatory Documents 80
12.4.3 Case Report Forms 80
12.4.4 Source Documents 80
12.5 Data Quality Control 81
12.5.1 Monitoring Procedures 81
12.5.2 Data Management 82
12.5.3 Quality Assurance/Audit 82
12.6 Study Termination 83
12.6.1 Premature Study Termination 83
12.7 Study Site Closure 84
12.7.1 Record Retention 84

12.8 Changes to the Protocol....84
12.9 Use of Information and Publication....84
13 FINAL CLINICAL STUDY REPORT....85
14 ETHICAL AND LEGAL CONSIDERATIONS....85
14.1 Good Clinical Practice....85
14.2 Subject Information and Informed Consent and/or Assent....85
14.3 Approval by Institutional Review Board or Ethics Committee....85
14.4 Finance and Insurance....86
15 REFERENCES....87
16 ATTACHMENTS....92
16.1 Investigator's Agreement....92
APPENDICES....93
APPENDIX A SCORING SHEET FOR SNAKEBITE SEVERITY INCLUSION CRITERIA* 94
APPENDIX B COMPOSITE SNAKEBITE SEVERITY SCORE FOR BASELINE AND OTHER EFFICACY ENDPOINTS....95
APPENDIX C STUDY-SPECIFIC REQUIREMENTS....96
Appendix C1a: Clinical Global Impression-Improvement (CGI-I) Form....97
Appendix C1b: Clinical Global Impression-Expected Recovery (CGI-ER)....98
Appendix C2: Clinical Global Impression – Severity of Illness (CGI-S) Scale....99
Appendix C3: Patient Global Impression of Change Scale....100
Appendix C4: Patient-Specific Functional Scale....101
Appendix C5: NPRS Form....103
Appendix C6: Columbia-Suicide Severity Rating Scale Forms....104
Appendix C7: Grip Strength....111
Appendix C8: Modes of Mechanical Ventilation....112
Appendix C9: Head-Lift Duration....113
Appendix C10: Analgesic Use....114
APPENDIX D COVID-19 PROTOCOL DEVIATIONS....115
APPENDIX E REGULATIONS AND GOOD CLINICAL PRACTICE GUIDELINES....116
1. Regulations....116
2. Good Clinical Practice Guidelines....116

2.1 List of In-text Tables

Table 1 Schedule of Events 9
Table 2 Comparison of Varespladib and Currently Available Serum-Based Antivenom Therapies 25
Table 3 Summary of Dosing 34
Table 4 Pharmacokinetic Sampling Schedule 60

2.2 List of In-text Figures

Figure 1 Study Schematic 13

LIST OF ABBREVIATIONS AND DEFINITION OF TERMS

Abbreviation	Definition
ANCOVA	Analysis of covariance
BUN	Blood urea nitrogen
CGI-I	Clinical Global Impression – Improvement
CGI-S	Clinical Global Impression – Severity of Illness
C-SSRS	Columbia-Suicide Severity Rating Scale
DDI	Drug-drug interaction
FDA	Food and Drug Administration
OHRO	Office of Human Research Oversight
IC_{50}	Half-maximal inhibitory concentration
IND	Investigational New Drug application
INR	International normalized ratio
IV	Intravenous
LFT	Liver function test
MOF	Multiple organ failure
MIP	Maximum inspiratory pressure
NIF	Negative inspiratory force
PD	Pharmacodynamics
PK	Pharmacokinetics
PLA_2	Phospholipase A2
PSFS	Patient-Specific Functional Scale
PT	Prothrombin time
PTT	Partial thromboplastin time
PTSD	Post-traumatic stress disorder
Q12H	Once every 12 hours
QoL	Quality of life
SOC	Standard of care
$sPLA_2$	Secreted phospholipase A_2
TEAE	Treatment-emergent adverse effect
U.S.	United States
WHO	World Health Organization

3 INTRODUCTION

3.1 Background and Rationale

The treatment of snakebite envenoming is an urgent, global unmet medical need. It is estimated that worldwide more than 5 million snakebite envenomings occur annually, causing significant morbidity (approximately 400,000 causing permanent deformities or amputation) and mortality (up to 138,000 causing death) (Gutierrez et al., 2017; Longbottom et al., 2018). Snakebite primarily affects poor, rural populations in tropical and sub-tropical regions of low and middle-income countries, where outcomes are adversely impacted by the snakebite victim's lack of proximity and/or access to healthcare, including access to currently available antivenom therapies (Longbottom et al., 2018). Although comparatively unusual in the U.S., snakebite envenoming incurs significant morbidity and occasional mortality with bad outcomes primarily associated with delays in care, young age, neurological deficit, thrombocytopenia and coagulation abnormalities (Gerardo et al., 2019).

Approximately 5.8 billion people live in regions that place them at risk for being bitten by a medically important snake (Longbottom et al., 2018) and it is estimated that > 75% of deaths from snakebite occur outside the hospital setting before victims can reach medical care. While not all snakebites result in envenoming with morbidity and mortality, often by the time patients receive the appropriate medical care in a hospital setting it is far too late and the toxicities of snakebite envenoming have substantially progressed and are no longer amenable to effective treatment by antivenom therapy (Vaiyapuri et al., 2013; Gerardo et al., 2019). To underscore the urgency of the global public health crisis posed by snakebite envenoming, in 2017 the World Health Organization (WHO) classified snakebite envenoming as a neglected tropical disease (Longbottom et al., 2018). Furthermore, the WHO's Snakebite Envenoming Working Group stated that "accelerating preclinical and clinical testing of promising prehospital adjunctive treatments, such as the phospholipase A_2 inhibitor varespladib or varespladib-methyl, as part of the WHO snake bite envenoming research agenda may lead to early improvements in prehospital survival" (Williams et al., 2019).

Even when the standard of care (SOC) antivenom therapies are available, their use is associated with a number of challenges and limitations, many of which could potentially be overcome with a small-molecule therapeutic (Bulfone et al., 2018) (summarized in Section 4.3). Indeed, because snake venom secreted phospholipase A2 ($sPLA_2$) is involved in virtually every critical venom effect and exists as a toxin in virtually all venomous snakes around the world (Tasoulis and Isbister, 2017), $sPLA_2$ has been identified as a desirable target for a toxin-specific approach to the treatment of snakebite (Williams et al., 2019).

The objective of the development program by Ophirex is to advance varespladib, a high-potency small-molecule inhibitor of $sPLA_2$, and its oral prodrug formulation (varespladib-methyl) for the treatment of snakebite envenoming. Varespladib-methyl (LY333013) is rapidly hydrolyzed into varespladib (LY315920). LY315920 is a potent inhibitor of $sPLA_2$ (Snyder et al., 1999). LY333013 is an orally bioavailable, methyl ester prodrug of the active $sPLA_2$ inhibitor LY315920, which is administered intravenously (IV).

Varespladib was originally developed as a $sPLA_2$ inhibitor for treating multiple diseases with an underlying inflammatory component (e.g., sepsis, rheumatoid arthritis, and cardiovascular disease) (Adis, 2011). These previous development efforts were terminated because of lack of efficacy or futility in diseases studied, but they have provided a substantial amount of clinical safety and bioavailability data, with > 4,600 subjects exposed to both varespladib and varespladib-methyl.

3.1.1 Scientific Rationale

In mammals, $sPLA_2$ represents a family of enzymes implicated in the activation of inflammatory process that produces multiple organ failure in sepsis (Anderson et al., 1994). $sPLA_2$ originates from a variety of cells including macrophages, neutrophils, and inflamed synovial tissue. In snake venom it originates from modified salivary glands and is a key toxin not reliably neutralized by antibody-based products such as antivenom (Prasarnpun et al., 2005). During envenoming, snake venom $sPLA_2$ proteins are injected in high concentrations directly into the victim with potentially rapid lethality and, frequently, severe hematological and/or persistent tissue damage due to hemotoxic and cytotoxic venom $sPLA_2$ toxins, respectively (Gutierrez et al., 2017).

$sPLA_2$s are the most pharmacologically active component of snake venoms and one of the components most responsible for lethality and long-term morbidity (Gutierrez et al., 2017). Venom $sPLA_2$ exhibits a wide spectrum of toxicities in snakebite victims which include neurotoxicity, myotoxicity, hemotoxicity, cytotoxicity, cardiotoxicity, homeostatic instability and edema (Tasoulis and Isbister, 2017; Fernandez et al., 2018). The toxicities of $sPLA_2$ in venom are related to the multiple functional binding sites on the surface of the molecule and the cellular receptors or lipids to which they bind (Kini, 2003).

Venoms from more than 95% of the world's venomous snake species utilize $sPLA_2$ enzymes as weaponry for predation and defense (Tanaka et al., 2010; Bocian et al., 2016; Tasoulis and Isbister, 2017). In addition to high enzymatic activity and characteristics resulting in poor neutralization by antivenom, $sPLA_2$ in venom can account for a high absolute percentage of venom content (Senji Laxme et al., 2019). In the Papuan black snake, *Pseudechis papuanus* (an elapid), $sPLA_2$ accounts for greater than 90% of venom content and 65 to 70% in many species of krait (*Bungarus spp.)* (Ziganshin et al., 2015). $sPLA_2$ may account for >60% of venom from *Dabioa russelii* (Russell's viper) and European adders such as *Vipera nikolskii* (viper), and 30 to 40% of the venom content in *Agkistrodon* (e.g., water moccasins and copperheads) (Lomonte et al., 2014; Tasoulis and Isbister, 2017; Pla et al., 2019). Nevertheless, due to $sPLA_2$'s potent enzymatic activity and an ability to diffuse from the circulation into interstitial spaces, even venoms with a seemingly low $sPLA_2$ content can be extremely toxic (Gutierrez and Lomonte, 2013; Tasoulis and Isbister, 2017; Senji Laxme et al., 2019).

Neurotoxic venom $sPLA_2$ is found in both viper and elapid venoms. Blockade of neuromuscular transmission in vertebrate skeletal muscle results in weakness and flaccid paralysis. Unaddressed, this may result in respiratory depression and death by respiratory failure, total cardiovascular collapse or, simply, obstructive asphyxiation (Ranawaka et al., 2013; Sehgal et al., 2022). Neurotoxic $sPLA_2$s are resistant to antivenom therapy especially

when there are delays in treatment and the relatively low molecular weight presynaptic toxins have left the circulation making them inaccessible to high-molecular weight antivenom antibodies (Prasarnpun et al., 2005; Anil et al., 2010; Senji Laxme et al., 2019).

The anticoagulant effects of venom $sPLA_2$ leads to dysregulation of the blood coagulation cascade (Alangode et al., 2020b). Other $sPLA_2$s in venom can cause instability in blood pressure or destruction of skeletal muscle followed by kidney failure. The hydrolysis of phospholipids by venom $sPLA_2$ and release of free-fatty acids appears to be the primary mechanism to account for the many enzymatic effects observed (Kini, 2006; Alangode et al., 2020b; Salvador et al., 2021), while other effects of $sPLA_2$ may be due to non-enzymatic interactions (Kini, 2006; Salvador et al., 2021). $sPLA_2$s in venom can also induce acute necrosis of skeletal muscle (myonecrosis) (Lomonte and Rangel, 2012), leading to permanent tissue loss or amputation (Otero et al., 2002; Gopalakrishnan, 2019). Further, venom $sPLA_2$ can act as a catalyst of activities of other venom components such as metalloproteases (Bickler, 2020).

The hemolytic, anticoagulant and consumptive effects of venom $sPLA_2$ lead to anemia, hypoxia, acute kidney injury and difficult to treat dysregulation of the coagulation cascade (Lewin et al., 2016; Bittenbinder et al., 2018; Lewin et al., 2018a; Lewin et al., 2018b; Xie et al., 2019; Alangode et al., 2020a; Xie et al., 2020b). The hydrolysis of phospholipids by venom $sPLA_2$ can be a primary mechanism to account for the anticoagulation effects observed in envenoming by elapids (Kini, 2006). However, even in the absence of phospholipids, some venom $sPLA_2$ subtypes still inhibit coagulation (Saikia et al., 2011). Venom $sPLA_2$ can also induce other toxicities in blood, such as myoglobinuria-inducing, platelet aggregation initiating/inhibiting activities (Kerns et al., 1999). The combination of hemolysis and venom-induced consumption of coagulation factors can lead to catastrophic homeostatic and hemodynamic instability (Park et al., 2020). While it is the $sPLA_2$-mediated neurotoxicity of snake venom that typically causes most rapid lethality in humans, venom $sPLA_2$s are also responsible for lasting medical challenges that arise from hemolysis, myonecrosis, kidney damage and pain (Arce-Bejarano et al., 2014; Gutierrez et al., 2017; Zambelli et al., 2017; Surm and Moran, 2021).

In vitro, varespladib and varespladib-methyl potently inhibit snake venom $sPLA_2$ with highly variable tertiary and quaternary structures at half-maximal inhibitory concentration (IC_{50}) in the nano- and sub-nanomolar range (Lewin et al., 2016). Several in vivo studies show that both the orally-administered varespladib-methyl and IV-administered varespladib rescue mice, rats and pigs from 100% lethal doses of coral snake venom, even when antivenom was no longer of benefit to the animals, returning platelet function to normal and reversing venom-induced hemolysis (Lewin et al., 2018a; Lewin et al., 2018b). Further, in vitro and in vivo results by others have replicated and extended the original finding of in vitro potency and broad-spectrum effect (Xiao et al., 2017; Bittenbinder et al., 2018; Bryan-Quiros et al., 2019; Ferraz et al., 2019; Alangode et al., 2020b; Albulescu et al., 2020; Xie et al., 2020a; Xie et al., 2020b; Youngman et al., 2020; Silva-Carvalho et al., 2021; Tan et al., 2021). In in vitro and in vivo studies, protection and/or recovery from venom-induced lethality, return of neurological function and recovery from venom $sPLA_2$- induced coagulation abnormalities were observed (Lewin et al., 2016; Bittenbinder et al., 2018; Lewin et al., 2018a; Lewin et al., 2018b). The structural basis for varespladib inhibition of snake venom $sPLA_2$ has

recently been established through crystallographic studies. In co-crystal structures, varespladib made specific, high-affinity contacts within the structurally conserved hydrophobic groove and Ca^{2+} binding active site of the venom $sPLA_2$. The conformationally restrained indole backbone and ketoamide substituent of LY315920 specifically bound a hydrophobic groove conserved across Group II $sPLA_2$ homologs, inhibiting catalytic phospholipase activity regardless of the species-specific $sPLA_2$ quaternary structure (Salvador et al., 2019; Barbosa et al., 2021; Salvador et al., 2021).

Together these studies demonstrate that 1) LY315920 inhibits the phospholipase activity of a broad-spectrum of venom $sPLA_2$ from a variety of snake species, 2) LY315920 and LY333013 block venom $sPLA_2$ activity in vitro and in vivo, 3) LY315920 or LY333013 provide therapeutic benefit throughout the time course of envenoming 4) LY315920 or LY333013 protect or reverse neurological damage caused by venom $sPLA_2$ and 5) LY315920 or LY333013 and antivenom combined may have a synergistic effect on $sPLA_2$ inhibition based on recent non-clinical studies (Silva-Carvalho et al., 2021).

A detailed summary of additional structural, in vitro, and in vivo studies supporting the use of LY315920 or LY333013 to neutralize venom $sPLA_2$ toxins is provided in the varespladib Investigator's Brochure (IB).

3.2 Clinical Experience

Multiple clinical studies have been performed by the previous sponsors for LY333013 (varespladib-methyl) and LY315920 (varespladib), Eli Lilly and Anthera, to characterize the safety, efficacy, pharmacokinetics (PK), and pharmacodynamics (PD) of LY315920 or LY333013. The inflammatory conditions studied clinically included sepsis, rheumatoid arthritis, ulcerative colitis, asthma, atherosclerosis, and acute chest syndrome in at-risk patients with sickle cell disease. Previous clinical studies have been summarized in the current version of the varespladib IB.

3.3 Summary of Potential Risks and Intent to Benefit

All subjects, regardless of randomized arm, will benefit from participation in this study through safety monitoring beyond the standard of care. Other benefits include added clinical oversight from local study physician(s), providers, nurses, and research staff, as well as medical personnel at the Sponsor and the Contract Research Organization. Continued monitoring/oversight starting with the subject entering the study, through day 28, from health professionals as part of the Post-Treatment Observation Period will provide subjects clinical monitoring well beyond hospital discharge and normal follow-up. Vital sign monitoring is a key aspect of this study, and due to the increased frequency of monitoring trends indicating worsening of condition will potentially be identified earlier. All subjects will receive a total of five 12-lead electrocardiograms. Additional laboratory tests to monitor liver function, cardiac function, renal function, coagulation, and hematology in addition to standard of care will increase safety monitoring of all subjects (Table 1). Because of the high-level of monitoring and oversight for patients within the study, these patients, including those randomized to placebo, will likely have more timely access to resources including clinician evaluation, procedures, and escalation of care if warranted.

For patients randomized to the intervention arm, there is the additional potential benefit of improved outcomes in terms of snakebite severity and quality of life (QoL) assessments. Table 2 describes the limitations of current serum-based antivenoms, and the potential advantages of varespladib and varespladib-methyl.

Table 2 Comparison of Varespladib and Currently Available Serum-Based Antivenom Therapies

Challenge or Limitation	Limitations of Serum-Based Antivenom Therapy	Potential Advantages of IV and Oral Varespladib
**Ease of Use**	Must be administered IV in advanced care clinical setting: cannot be self-administered at the time of snakebite<br><br>Intensive monitoring for antivenom induced complications required	IV and oral formulations can be administered in combination with antivenom therapies not able to adequately neutralize venom sPLA2
**Species Specificity**	Generally specific to one species or closely related species of venomous snakes and sPLA2 is poorly antigenic	Varespladib is a potent, broad-spectrum inhibitor of snake venom sPLA2
**Tissue Penetration**	Large proteinaceous molecules with limited or no ability to penetrate tissues	Small molecule with the ability to penetrate tissues to neutralize or reverse sPLA2-mediated venom toxicities
**Safety/ Allergenicity**	High (23% - 56% acute allergic reactions)	Low (no documented allergies)
**Cost to Manufacture**	Higher	Lower
**Requirement for Refrigeration**	Yes (most formulations)	No refrigeration
**Available Global Supply**	Current and anticipated long-term global shortage (Williams et al., 2019)	Potential for a comparably broader, more cost-effective, global distribution

Abbreviations: IV = intravenous; $sPLA_2$ = secreted phospholipase A2.

The proposed treatment is the sequential administration of the intravenous formulation by continuous rate infusion (CRI) followed by the oral formulation of varespladib. The initial dosing consists of a CRI of varespladib at 0.45 mg/kg/hr for adults and 0.6 mg/kg/hr for pediatric patients aged 5 to 17 years for 6 hours. After 6 hours, the patient will be assessed for transition to oral IP. If the subject meets clinical criteria, the infusion will be stopped, and the oral IP will be initiated.

Recommended clinical criteria for stopping the infusion and initiating oral IP include the following:

1. SSS Nervous System Score ≤ 1;
2. clinically stable as judged by the treating physician;
3. able to tolerate oral intake.

If the subject does not meet clinical criteria, the infusion will continue. Thereafter, the subject will be reassessed at least twice a day, and transitioned to the oral IP when they meet clinical criteria. All subjects will switch to oral treatment no later than 48 hours, unless the subject is intubated and unable to receive oral drug via nasogastric tube. Regardless of when the subject transitions from intravenous to oral therapy, the total duration of therapy starting from initiation of intravenous IP for all patients will be 7 days.

When the subject transitions to the oral IP, adults will be given an initial dose of 500 mg varespladib-methyl (2 x 250 mg tablets). Pediatric subjects aged 12 to 17 years will receive an initial dose of 400 mg varespladib-methyl (8 x 50 mg capsules). Pediatric subjects aged 5 to 11 years will receive an initial dose of 200 mg varespladib-methyl (4 x 50 mg capsules). Thereafter, subjects will take oral doses every 12 hours (q12h) for a total duration of 7 days from the initiation of the infusion. Adults will receive 250 mg varespladib-methyl (1 x 250 mg tablet) q12h, pediatric subjects aged 12 to 17 years will receive 200 mg varespladib methyl (4 x 50 mg capsules) q12h, and pediatric subjects aged 5 to 11 years will receive 100 mg varespladib methyl (2 x 50 mg capsules) q12h.

Formulation, Dose	Age 5 to 11 years	Age ≥12 and <18 years	Age ≥18 years
Intravenous	0.6 mg/kg/hr	0.6 mg/kg/hr	0.45 mg/kg/hr
Oral, first dose	200 mg (4 x 50 mg capsules)	400 mg (8 x 50 mg capsules)	500 mg (2 x 250 mg tablets)
Oral, all doses after the first dose	100 mg (2 x 50 mg capsules)	200 mg (4 x 50 mg capsules)	250 mg (1 x 250 mg tablet)

The doses proposed for this study are anticipated to be safe and tolerable. The potential risks of study participation include those associated with varespladib, including adverse events (AEs) and serious adverse events (SAEs) as well as the risks of medical evaluation and treatment, including the minimal possibility of allergic reaction from the study drug. Across clinical studies, varespladib was tolerated well and generally did not induce AEs beyond those observed in placebo treatment groups, even in severely ill subjects. In a total of seven phase 1 and three phase 2 studies, IV varespladib was administered to 733 subjects. In a total of twelve phase 1 and five phase 2 studies oral varespladib-methyl was administered to a total of 3,679 subjects. Common AEs observed included rhinitis, nausea, and diarrhea. No clinically meaningful varespladib or varespladib-methyl treatment-related effects were noted for biochemical and hematologic parameters other than modest transient increases in liver enzymes that occurred in several subjects. Treatment with varespladib or varespladib-methyl was not associated with changes in heart rate or blood pressure and there was no evidence of

an effect on ventricular repolarization (QT interval prolongation). SAEs including cerebrovascular accident (CVA) and myocardial infarction (MI) have been observed in some acutely ill subjects in clinical studies for indications such as severe sepsis and acute coronary syndrome; detailed descriptions of these SAEs can be found in the Varespladib Investigator's Brochure (IB). Patients with conditions such as these are excluded from participation in this study.

Varespladib-methyl (LY333013) was administered to 45 subject in an Ophirex-sponsored, Phase 2, randomized, double-blind, placebo-controlled clinical trial (OPX-PR-01) of patients with snakebite envenoming. In that trial, there were no deaths, no treatment emergent serious adverse events, and no patients were discontinued from the study drug or unblinded by an investigator. The percentage of subjects experiencing a treatment emergent adverse event was lower in the varespladib-treated subjects than in the placebo-treated subjects (38% vs. 55%, p-value accounting for follow-up time = 0.08).

In subjects receiving continuous IV varespladib (LY315920) infusion, doses of up to 77 mg/kg over 7 days (0.46 mg/kg/hr) were tolerated well and treatment-related AEs were mild to moderate and were not clinically significant. Based on pharmacokinetic models, the proposed IV dosing in pediatric subjects of 0.6 mg/kg/hr will result in drug levels that are equivalent to those resulting from the dose of 0.45 mg/kg/hr given to adult subjects. Multiple oral doses of varespladib-methyl tablets (LY333013) up to 500 mg q12h were tolerated well. Thus, short-term treatment with varespladib or varespladib-methyl at doses up to 0.45 mg/kg/hr (IV) or500 mg q12h (oral) is not anticipated to induce clinically significant AEs or SAEs. Thus, the proposed protocol of administering the study drug by CRI at 0.45 mg/kg/hr and transitioning to oral dosage form with initial 500 mg varespladib-methyl followed by 250 mg q12h or the equivalent dosing in subjects aged 12 to 17 years (400 mg followed by 200 mg q 12h) and aged 5 to 11 years (200 mg followed by 100 mg q 12 h) is not anticipated to cause AEs, even in subjects with severe illness resulting from snakebite envenoming.

A summary of the pharmaceutical properties and known potential risks of varespladib-sodium and varespladib-methyl is provided in the current version of the varespladib IB. The Investigator must become familiar with all sections of the IB before the start of the study.

4 OBJECTIVES AND ENDPOINTS

Objectives	Endpoints
**Primary Objective**	**Primary Efficacy Endpoint**
• To evaluate the efficacy of intravenous (IV) varespladib in addition to standard of care (SOC) in subjects bitten by venomous snakes.	• For subjects bitten by elapids: Time to complete head-lift recovery defined by a 5-second head-lift of 5 seconds.<br>• For subjects bitten by vipers: Area Under the Curve (AUC) of an abbreviated Snakebite Severity Score (SSS) composed of local wound, hematologic, and neurologic subscores from Baseline (pre-dosing) to Day 14.<br>• The independent p-values from the elapids and the vipers will be analyzed using Fisher's combined probability test to obtain a single global p-value for testing the primary endpoint.
**Secondary Objectives**	**Secondary Efficacy Endpoints**
• To evaluate efficacy of varespladib and varespladib-methyl as treatment for $sPLA_2$-induced venom toxicities	• Area under the curve of the composite outcome of the pulmonary, cardiovascular, local wound, hematologic, renal, and nervous system sections of the snakebite severity score (SSS) from Baseline (pre-dosing) to Day 7 for all patients and for patients randomized in ≤5 hours from bite.<br>• Patient Specific Functional Scale (PSFS) score at Day 3 and Day 7.<br>• Complete snakebite severity score (SSS) recovery, defined as a composite score of 0 for the pulmonary, cardiovascular, local wound, hematologic, renal, and nervous system sections of the SSS at Day 28.<br>• Total antivenom requirement from Baseline through Day 28<br>• Duration of hospitalization from Baseline through Day 28<br>•
**Exploratory Objectives**	**Exploratory Endpoints**
• To further understand the effects of varespladib and varspladib methyl on outcomes in patients with snakebite envenoming	• Change in the composite outcome of the 6-item SSS (pulmonary, cardiovascular, local wound, hematologic, renal, and nervous system subscores) from Baseline through Day 28<br>• Change in the 6-item SSS from Baseline through Day 28 using compressed SSS scale<br>• Individual SSS subscores from Baseline through Day 28<br>• Focused hematologic score based on INR and platelets from Baseline through Day 28<br>• Coagulation abnormalities (separately PT, PTT, platelets, and fibrinogen) from Baseline through Day 28<br>• Change in NPRS from Baseline through Day 28<br>• Clinical Global Impression-Improvement (CGI-I) from Baseline through Day 28<br>• Patient Global Impression of Change (PGIC) from Baseline through Day 28<br>• Patient-Specific Functional Scale (PSFS) average score from Baseline through Day 28<br>• Levels of the myonecrosis marker, creatine kinase (CK), from Baseline through Day 3

<table>
<tr><td></td><td>

For patients bitten by elapids, change in 5-second head-lift duration from Baseline through Day 28
For patients bitten by elapids, grip strength from Baseline through Day 28
For patients bitten by elapids, duration of ventilatory support from Baseline through Day 28
Analgesic use from Baseline through Day 28
Transfusion requirement from Baseline (Day 1) through Day 28
Elapsed time from initiation of IV varespladib treatment until the transition to oral varespladib-methyl
All cause mortality through Day 28
Secretory phospholipase A2 (sPLA2) from Baseline through Day 7

</td></tr>
<tr><td>Objectives</td><td>Endpoints</td></tr>
<tr><td colspan="2">Pharmacokinetics Evaluation</td></tr>
<tr><td>
To assess pharmacokinetics (PK) of varespladib and varespladib-methyl in subjects with snakebite envenoming
</td><td>
PK parameters of varespladib in plasma from all study subjects using the following schedule. PK analysis will be conducted using population PK modeling methods.
<table>
<tr><th>IV</th><th>Oral
1st dose</th><th>Oral
2nd dose</th><th>Day 7 (Oral)</th></tr>
<tr>
<td>Pre-dose
0.5h post initiation
End of infusion</td>
<td>2 hours after first oral dose
Either 5-10 hours after first oral dose OR after 2nd, 3rd, or 4th in-hospital oral dose</td>
<td>If hospitalized: Sample prior to second oral dose
OR
If discharged prior to second dose: Sample prior to discharge</td>
<td>Pre-Dose</td>
</tr>
</table>
</td></tr>
<tr><td>Safety</td><td>Safety and Tolerability Endpoints</td></tr>
<tr><td></td><td>

Incidence and severity of adverse events (AEs), serious AEs (SAEs), and AEs leading to discontinuation of Investigational Product (IP)
Safety of varespladib-methyl as assessed by the number and rates of reported treatment-emergent adverse events (TEAEs) from beginning of treatment until last Follow-Up Visit/Telephone call at Day 28
Number of subjects with a treatment-related SAE from beginning of treatment until last Follow-Up Visit/Telephone call at Day 28
Safety as assessed by

Vital signs
Clinical laboratory evaluations: CBC, urinalysis, liver function tests [LFTs], renal function tests (creatinine, blood urea nitrogen [BUN], estimated glomerular filtration rate [eGFR])
12-lead electrocardiogram (ECG)

Concomitant medications and therapies
Columbia-Suicide Severity Rating Scale (C-SSRS) evaluated at Baseline or at the earliest time point clinically allowable (ideally Day 1) and then at every study visit through Day 28

</td></tr>
</table>

5 STUDY DESIGN

5.1 Overall Study Design and Plan

This is a multicenter, randomized, double-blind, placebo-controlled, phase 2 study designed to evaluate the safety, tolerability and efficacy of a continuous rate infusion (CRI) of IV varespladib followed by transition to the oral dosage form, varespladib-methyl, concurrently with SOC, in subjects bitten by venomous snakes.

Approximately 140 male and female subjects will be randomized to receive active varespladib or placebo (in addition to SOC) in a 1:1 ratio. Randomization will be stratified by snake type (copperhead, Mojave rattlesnake, non-Mojave rattlesnake, Russell's viper, Elapid, or other), and by age group: pediatrics aged 5 to 17 years or adults aged 18 years and older and resulting in 12 strata in total.

Patients eligible for the study must be male or female ≥ 5 years of age with venomous snakebite. The index event (snakebite) must be symptomatic and the patient must be willing (or legally authorized representative is willing) to provide informed consent prior to initiation of any study procedures. In India, inclusion will be limited to patients bitten by known or suspected Russell's viper or krait.

Patients must meet one of two categories of inclusion criteria:

> **Category 1**: The patient is enrolled within 5 hours of venomous snake bite or symptom onset with an SSS score* of ≥2 in one system and ≥1 in another system (2+1).
>
> OR
>
> **Category 2**: The patient has a suspected or confirmed bite from an elapid and is enrolled within 10 hours of bite or symptom onset with moderate to severe cranial nerve or skeletal muscle weakness**.

* Only local wound, pulmonary, cardiovascular, hematologic, or nervous system scores qualify for SSS inclusion criteria. Hematologic score may be used if available, but inclusion should not wait for laboratory results. Point of care tests (e.g., 20WBCT) may be used for enrollment, if used per site standard of care (see Appendix A). GI and Renal scores are not used for inclusion.

** Isolated ptosis does not meet the definition of moderate to severe neurotoxicity for enrollment.

One group will be treated with the SOC plus placebo and the other treated with SOC plus varespladib-methyl. The time between baseline and eligibility assessments, randomization, and IP administration should be minimized.

SSS scoring should be performed for inclusion assessment without waiting for receipt of Baseline laboratory results, see Appendix A for scoring checklists. One group will be treated with the SOC plus placebo and the other treated with SOC plus varespladib. There should be no delay in providing SOC. Sites are encouraged to develop and implement processes that will allow for initiation of IP as soon as possible after enrollment.

Primary efficacy outcome will be assessed separately for subjects bitten by elapids and patients bitten by vipers. For subjects bitten by elapids: Time to complete head-lift recovery defined by a 5-second head-lift of 5 second. For subjects bitten by vipers: Area Under the Curve (AUC) of an abbreviated Snakebite Severity Score (SSS) composed of local wound, hematologic, and neurologic subscores from Baseline (pre-dosing) to Day 14. The independent p-values from subjects bitten by elapids and subjects bitten by vipers will be analyzed using Fisher's combined probability test to obtain a single global p-value for testing the primary endpoint.

Additional details will be provided in the SAP.

Safety and tolerability will be assessed by evaluating AEs, vital sign measurements, clinical laboratory test results, ECGs, physical examination findings, and concomitant medications and therapies.

All AEs observed by the study personnel or reported by the subject during the study (from the time of the signing of the informed consent and/or assent through the End of Study [EOS] visit) will be documented.

Pharmacokinetic sampling will be performed in all subjects at timepoints specified in Section 9.3.2.1.

5.2 Rationale and Discussion of Study Design

Snakebite envenoming represents a deadly and unmet global medical need. While antivenoms comprise the current SOC for treatment of snakebites, they suffer from several limitations including specificity of each antivenom for specific species of snake, limited access to antivenom in rural areas, costly or impractical storage requirements, and delays in administration. Treatment of snakebite envenoming with the small-molecule drug varespladib has the potential to overcome several limitations of serum-based antivenoms that underpin traditional SOC.

The proposed study in the United States and India will provide coverage of a broad-spectrum of venomous snake genera, including elapids, vipers, and pit vipers in differing geographies and differing $sPLA_2$ structures. Recognizing that each snake type has a unique clinical profile and recognizing the large number of venomous snake species in India, enrollment in India is limited to patients bitten by Russell's viper or krait. These two snake types account for the majority of snakebite-related deaths in India and present with representative clinical syndromes. For elapid bites (e.g., krait and coral snake), because the bite is subcutaneous and venom uptake is via lymphatics, delays in presentation for care are common. Accordingly, enrollment for these patients is extended to within 10 hours of bite or symptom onset. Study sites will be selected based on demonstrated historical incidence of snake bites from species deemed relevant to this study, to ensure a broad range of envenoming toxins are expected to be encountered in potential study subjects.

This study was designed to exclude as few critically ill subjects as possible. The primary criteria for participation are that subjects must be ≥ 5 years of age, a sufficient severity of snakebite envenoming based on their SSS score (see Section 6.1), must not be pregnant or breast-feeding, and are not taking excluded medications.

The study design allows for both treatment arms (varespladib and placebo) to receive SOC (e.g., antivenom) concurrently. Thus, critically ill subjects may receive emergency treatment in a timely manner while being evaluated for the potential clinical benefit associated with inhibition of venom $sPLA_2$ by varespladib.

Because subjects with severe snakebites are initially evaluated in emergency departments and then admitted to the hospital, this study was designed to screen, enroll, and administer treatment during a single emergency department visit and associated hospitalization. Once a subject is stable and has progressed to oral administration, subjects who demonstrate substantial improvement may be discharged from the hospital according to institutional practice. Discharged patients will be expected to continue the investigational product (IP) treatment in an outpatient setting for a total of 7 days from start of the IV treatment.

Risks associated with the control (placebo) arm of this study include the same risks associated with SOC (antivenom).

The efficacy endpoints for this study were selected to evaluate if varespladib plus SOC is superior to SOC alone. The primary endpoint was selected to evaluate clear clinical benefit based on SSS (Dart et al., 1996), whereas secondary and exploratory endpoints were selected to evaluate sustained improvements in each subject's snakebite severity as well as QoL, healthcare utilization, and mortality during the month following the bite. The primary efficacy endpoint evaluates the efficacy of the IV form of varespladib over the course of 6 hours of IV administration. Additional analyses will assess the efficacy over the entire duration of the study. PK analysis will be performed at specified timepoints throughout the treatment period.

5.3 Selection of Doses in the Study

This study evaluates the safety, tolerability, and efficacy of varespladib in subjects bitten by venomous snakes from diverse regions and snake genera, including (but not limited to) pit vipers (e.g., rattlesnake, copperhead), vipers (e.g., Russell's viper), and elapids (e.g., coral snake, krait).

5.3.1 Study Design Rationale

LY315920 (varespladib) is a lyophilized drug packaged in vials containing 100 mg of active drug. LY333013 (varespladib-methyl) is formulated in an immediate-release film-coated oval tablet at a unit dosage strength of 250 mg for oral administration. Ophirex is developing a sequential treatment approach using intravenous varespladib (LY315920) followed by oral varespladib-methyl (LY333013) for 7 days total for the treatment of venomous snakebites. LY333013 is a prodrug that is rapidly metabolized to LY315920; LY315920 is a potent inhibitor of $sPLA_2$ in vitro and in vivo.

The proposed dosing of varespladib consists of an initial continuous infusion of 0.45 mg/kg/hr for adult subjects and 0.6 mg/kg/hr for pediatric subjects for 6 hours. This infusion will be continued until the subject meets clinical criteria for transition to oral IP or until 48 hours, at which time all patients will switch over to the oral drug unless a patient is

intubated and is unable to receive medication via nasogastric tube due to gastrointestinal bleeding, hemodynamic instability, or the decision of the treating provider. The first assessment to determine if the subject is ready to transition to the oral formulation will occur at 6 hours. Subsequent assessments will occur at least twice a day thereafter.

If the subject meets clinical criteria and is able to take the oral drug, the infusion will be stopped, and the oral drug will be initiated. Recommended clinical criteria for stopping the infusion will include the following:

1. SSS Nervous System Score ≤ 1;
2. clinically stable as judged by the treating physician;
3. able to tolerate oral intake.

If the subject does not meet these clinical criteria, the infusion will continue. Thereafter, the subject will be reassessed at least twice a day, and transitioned to the oral drug when they meet the clinical criteria for stopping infusion and are able to take the oral drug. The maximum duration of IV study drug is 48 hours (i.e. all subjects will switch over to the oral drug by 48 hours), unless a subject is intubated and is unable to receive medication via nasogastric tube. Regardless of when the subject transitions from intravenous to oral therapy, the total duration of therapy for all patients will be 7 days.

When transitioned to the oral dose, adult subjects will receive an initial oral dose of 500 mg varespladib-methyl (2 x 250 mg tablets) or placebo, followed by 250 mg varespladib-methyl (1 x 250 mg tablet) or placebo q12h for the remainder of the 7-day treatment period. Pediatric subjects aged 12 to 17 years inclusive will receive 400 mg varespladib-methyl (8 x 50 mg capsules) or placebo, followed by 200 mg varespladib-methyl (4 x 50 mg capsules) or placebo q 12 h. Pediatric subjects aged 5 to 11 years inclusive will receive 200 mg varespladib-methyl (4 x 50 mg capsules) or placebo, followed by 100 mg varespladib-methyl (2 x 50 mg capsules) or placebo q 12 h. The initiation of oral dosing should take place immediately after the end of IV infusion.

All subjects will receive treatment for a total of 7 days regardless of when they transition to the oral drug. Final dosing may occur on 7 or 8 calendar days, depending on whether the IV infusion was initiated in the morning or the afternoon.

Oral doses should be taken with food.

A summary of formulations and dosing for subjects is provided in Table 3.

Table 3 Summary of Dosing

**Age ≥ 18 yr (Adult)**	
**IV Dose**	0.45 mg/kg/hr
**Oral Formulation**	Tablet
**Initial Oral Dose**	500 mg (2 x 250 mg)
**Oral Dose (q12h)**	250 mg (1 x 250 mg)
**Age 12-17 yr (Pediatric)**	
**IV Dose**	0.6 mg/kg/hr
**Oral Formulation**	Capsule
**Initial Oral Dose**	400 mg (8 x 50 mg)
**Oral Dose (q12h)**	200 mg (4 x 50 mg)
**Age 5-11 yr (Pediatric)**	
**IV Dose**	0.6 mg/kg/hr
**Oral Formulation**	Capsule
**Initial Oral Dose**	200 mg (4 x 50 mg)
**Oral Dose (q12h)**	100 mg (2 x 50 mg)

Abbreviations: yr = year; q12h = once every 12 hours

5.3.2 Dose Justification

Normal levels of endogenous human $sPLA_2$ are approximately 2 to 5 ng/mL, but may increase 1000 fold to approximately 3 µg/mL in patients with severe sepsis or septic shock. In contrast, direct injection of snake venom can result in plasma concentrations of $sPLA_2$ substantially higher than 3 µg/mL. PK/PD models of envenoming suggest that a catastrophic snakebite injecting 200 mg total $sPLA_2$ (e.g., from the bite of an extremely large taipan with venom composed of > 80% $sPLA_2$) may result in plasma $sPLA_2$ concentrations up to 15 µg/mL in humans, whereas envenoming with a coral snake bite (i.e., 2.5 mg total $sPLA_2$) would result in $sPLA_2$ concentrations nearly 1000-fold lower (BSSA Modeling Report). The sPLA2 plasma concentrations resulting from bites from other snakes have not been well characterized.

An IV infusion of varespladib at 0.45 mg/kg/hr followed by oral dosing of 500 mg then 250 mg q12h is safe based on prior human safety studies of infusions exceeding this amount for up to 7 days with no clinically significant AEs or alternations in laboratory data suggestive of any safety concerns (J4A-LC-EZZB; -BWWB; -BWXC; OPX-VAR-PMX-2). This dosing approach is also anticipated to result in (1) molar concentrations of LY315920 approximately 5 times the highest concentrations of $sPLA_2$ likely to result from snake envenoming, and (2) clinically significant inhibition of $sPLA_2$ from most snakebite envenomings throughout the total venom exposure (including initial plasma $sPLA_2$ concentrations resulting from an injection of up to 200 mg total venom $sPLA_2$) (BSSA Modeling Report). Once the infusion of 0.45 mg/kg/hr reaches steady state, the anticipated plasma concentration of LY315920 is approximately 1900 ng/mL, which is anticipated to be sufficient to fully inhibit $sPLA_2$ toxin levels resulting from envenoming by all medically important snake species (Study Report J4A-LC-EZZB; OPX-VAR-PMX-2).

Upon transition to the initial oral dose of 500 mg, the plasma concentrations will not be higher than serum levels achieved from the initial IV infusion. Concentrations following consecutive 250 mg BID varespladib-methyl will remain sufficiently high (approximately 600 ng/mL) to maintain complete inhibition of residual venom $sPLA_2$ toxin. Previously, an intravenous dose up to 0.46 mg/kg/hr for 7 days or an oral dose of up to 500 mg BID for 8 weeks have been used for adult subjects throughout the clinical development of LY315920 and LY333013 and are known to be safe and well tolerated.

A physiologically-based pharmacokinetic (PBPK) model predicts pediatric patients aged 5 to <18 years require dose adjustment to a 0.6 mg/kg/hr IV infusion to result in steady state concentrations that are bioequivalent to the concentrations predicted for adult patients at 0.45 mg/kg/hr. Similarly, when pediatric patients switch from IV to oral therapy, the model indicates that an initial oral dose of 400 mg followed by 200 mg q12 hour for 7 days is required for pediatric patients aged 12 to <18 years, and half that dose is required for pediatric patients aged 5 to <12 years, to result in plasma concentrations that are bioequivalent to the concentrations predicted for adult patients at 500 mg followed by 250 mg q12 hour oral dosing for 7 days. (See Development of a PBPK Model for Varespladib with the Simcyp Population-Based Simulator and Simulation of the Systemic Exposure and Dose of Varespladib in Adults and Pediatric Subjects Using In Vitro and In Vivo Data. 12 February 2024. Certara UK, Simcyp Division.)

This study will include patients who have already received antivenom but have persistent, life-threatening signs and symptoms of snakebite envenoming. Persistent signs and symptoms after antivenom treatment may result from several conditions: (1) a high volume of venom injected; (2) a delay from the time of the bite until the time of initial antivenom treatment, allowing venom to extravasate and penetrate various tissues including neuromuscular junction; (3) therapeutic gaps in the neutralization potential of current antivenoms due to the regional, inter-, and intra-species variation in the protein component of snake venoms; (4) the presence of neurotoxicity, which is generally resistant to antivenom therapy. Varespladib plasma concentrations within OPX-PR-03 are designed to provide complete inhibition of $sPLA_2$ and resolution of clinical toxicity at a dose shown to be safe in previous clinical studies.

In sum, the proposed dose for adult subjects balances the predicted clinically significant inhibition of sPLA2 in venom with a well-documented safety profile of varespladib and varespladib-methyl.

5.4 Study Sites

The study will take place at approximately 8 to 14 sites in the United States and 8 to 14 sites in India. Each site is anticipated to screen enough subjects to randomize approximately 5 to 20 subjects. Study site activation and enrollment periods will be planned to allow maximum enrollment during the most active seasonal snakebite months in each region. A study site with a high recruitment rate may be allowed to recruit more subjects if other sites have slow enrollment.

5.5 End of Study Definition

The EOS is the date when the last subject's last study visit occurs.

6 STUDY POPULATION

6.1 Selection of Study Population

Investigators or their designees will maintain a screening log of all candidates who are considered for participation in this study, including reasons for ineligibility or refusal to participate. Consent, screening, randomization, and dosing will occur on the same day. Subjects who do not meet all the eligibility criteria will not be enrolled. This study is estimated to enroll and randomize 140 male and female subjects equally into 2 groups with 10% withdrawal or loss to follow-up anticipated through the time of the assessment of the primary outcome.

6.2 Study Entry Criteria

6.2.1 Inclusion Criteria

A subject will be eligible for study participation if he or she meets all the following criteria:

1. Is a male or female ≥ 5 years of age.
2. Patients must have known or suspected venomous snakebite. In India, enrollment will be restricted to patients bitten by suspected or confirmed Russell's viper (*Daboia russelii*) or krait (*Bungarus spp.*). In the U.S., any snakebite that meets all other criteria may be eligible.
3. Patients must meet one of two categories of inclusion criteria:

   Category 1: The patient is enrolled within 5 hours of venomous snakebite or symptom onset with an SSS score* of ≥2 in one system and ≥1 in another system (2+1).

   OR

   Category 2: The patient has a suspected or confirmed bite from an elapid and is enrolled within 10 hours of bite or symptom onset with moderate to severe cranial nerve or skeletal muscle weakness.**
4. Is willing (or legally authorized representative is willing) to provide informed consent prior to initiation of any study procedures.

* Only local wound, pulmonary, cardiovascular, hematologic, or nervous system scores qualify for SSS inclusion criteria. Hematologic score may be counted if available, but inclusion should not wait for laboratory results. Point of care tests (e.g., 20WBCT) may be used for enrollment, if used per site standard of care (see Appendix A). GI and Renal scores are not used for inclusion.

** Isolated ptosis does not meet the definition of moderate to severe neurotoxicity for enrollment.

6.2.2 Exclusion Criteria

A subject will be excluded from the study if he or she meets any of the following criteria:

1. Has history of or is suspected to have CVA or intracranial bleeding of any kind, acute coronary syndrome, MI, or severe pulmonary hypertension.
2. Has known history of inherited bleeding or coagulation disorder.
3. Is, at Screening Visit, using the following anticoagulants: warfarin/coumadin, argatroban, bilvalirudin, lepirudin, apixaban, dabigatran, clopidogrel, prasugrel, ticlodipine or another anticoagulant agent not specifically listed, or has used heparin, enoxaparin, fondaparinux, or other low molecular weight heparin or any antiarrhythmic drugs within 14 days prior to treatment.
4. Has a history of chronic liver disease such as chronic active viral hepatitis, alcohol-related liver disease, non-alcoholic steatohepatitis, non-alcoholic fatty liver disease, hemochromatosis, primary biliary cirrhosis, primary sclerosing cholangitis, autoimmune hepatitis.
5. Reports or has known pre-existing renal impairment or chronic kidney disease.
6. Has a known allergy or significant adverse reaction to varespladib or varespladib-methyl.
7. Is considered by the Investigator to be unable to comply with protocol requirements due to geographic considerations, psychiatric disorders, or other compliance concerns.
8. Is pregnant, has a positive serum human chorionic gonadotropin (hCG) pregnancy test or not willing to use a highly effective method of contraception for 14 days after initial treatment, or is breast-feeding.

6.3 Early Subject Withdrawal

All subjects are free to withdraw from participation in this study at any time, for any reason. The Investigator should make every reasonable attempt to keep subjects in the study; however, subjects must be withdrawn from the study if they withdraw consent to participate. Investigators must attempt to contact subjects who fail to attend scheduled visits by telephone or other means to capture any AEs and determine whether a subject should be withdrawn. Every possible attempt should be made to collect and report AEs upon withdrawal, as described in Section 10.2.

The sponsor reserves the right to withdraw a subject due to protocol deviations or other reasons.

If a subject is withdrawn before completing the study, the reason for withdrawal and the date of study discontinuation will be recorded on the appropriate page of the electronic case report form (eCRF). Whenever possible and reasonable, the evaluations that were to be conducted at the completion of the study should be performed at the time of withdrawal.

The reason for subject discontinuation from study medication or withdrawal from the study will be recorded on the eCRF. Subjects who sign the informed consent form and are randomized but do not receive the study intervention may be replaced. Subjects who sign the informed consent form, are randomized and receive the study intervention, and subsequently withdraw or are withdrawn or discontinued from the study will not be replaced.

6.4 Discontinuation of Study Intervention

An Investigator may discontinue a subject from study treatment for the following reasons:

- Pregnancy
- Significant study intervention or study procedure noncompliance
- If any clinical AE, laboratory abnormality, or other medical condition or situation occurs such that continued participation in the study would not be in the best interest of the subject (if there is reasonable evidence of clinical benefit to justify continuation on the protocol, this must be discussed with the Ophirex medical monitor)
- If the subject meets an exclusion criterion (either newly developed or not previously recognized) that precludes further study participation

If a clinically significant finding is identified (including, but not limited to, changes from Baseline) after enrollment, the Investigator or qualified designee will determine if any change in subject management is needed. Specifically, this includes withdrawal of subjects who were initially enrolled but whose history of chronic liver disease, renal laboratory tests, or pregnancy tests suggest they are subject to exclusion criteria 6, 7, or 10.

Discontinuation from IP does not mean discontinuation from the study, and remaining study procedures should be completed as indicated by the study protocol.

Section 13.6 describes procedures in the event of suspension or termination of the study for all subjects.

6.5 Subject Replacement Criteria

Withdrawn subjects will not be replaced. If a substantial number of subjects are withdrawn from the study, the sponsor will evaluate the need for developing replacement criteria.

Randomized subjects withdrawn from the study may not reenter. The subject number for a withdrawn subject will not be reassigned to another subject.

7 TREATMENTS

7.1 Identification of Investigational Products or Placebo

The IP used in this study is varespladib or varespladib-methyl along with matching placebo. Varespladib will be provided as an IV formulation and varespladib-methyl as an oral formulation.

- Varespladib (IV)
  - Varespladib (LY315920) is a lyophilized drug contained in 100 mg vials to be reconstituted in Water for Injection (WFI), followed by dilution into 0.9% Sodium Chloride Injection.

- Varespladib will be administered by intravenous infusion at a continuous rate of 0.45 mg/kg/hr for adults and 0.6 mg/kg/hr for pediatric patients aged 5 to 17 years.

- Varespladib-methyl (oral)
  - Tablet: Varespladib-methyl (LY333013) is an immediate-release, oval, film-coated tablet at a dosage strength of 250 mg for oral administration.
  - Capsule: Varespladib-methyl (LY333013) is formulated as an immediate-release capsule (50 mg).
- Placebo
  - The intravenous placebo will be saline (0.9%). Blinding will be ensured by covering the bag containing the investigational product with opaque covers.
  - The oral placebo tablet is supplied as a film-coated oval tablet to match the appearance of the LY333013 250 mg tablet and contains only the excipients present in the active tablet formulation: lactose monohydrate, microcrystalline cellulose, and magnesium stearate.
  - The oral placebo capsule is supplied as an immediate-release capsule to match the LY333013 50 mg capsule for oral administration and contains only the excipients: lactose monohydrate, microcrystalline cellulose, and magnesium stearate.

7.2 Selection of Timing of Dose for Each Subject

Varespladib, varespladib-methyl, or matching IV or tablet placebo will be administered to subjects with suspected or confirmed snakebite envenoming:

- Adult and pediatric subjects will receive a CRI at 0.45 mg/kg/hr and 0.6 mg/kg/hr, respectively, for a minimum of 6 hours. After 6 hours, the patient will be assessed. The recommended clinical criteria for stopping the IV infusion are a Nervous System subscore ≤ 1, judged clinically stable by the treating provider, and able to tolerate POs. Once the infusion is stopped, the oral dosage form, varespladib-methyl, will be administered. If the subject does not meet these criteria, the infusion will be continued. The subject will be reassessed at 12 hours and thereafter reassessed q12h, and transitioned to the oral drug when they are clinically improved and able to take the oral drug. All subjects will switch to the oral drug at 48 hours unless intubated and unable to receive oral medical via nasogastric tube. The maximum duration of the infusion will be a total of 7 days.

- When the subject transitions to the oral drug, adults will be given an initial dose of 500 mg varespladib-methyl (2 x 250 mg oral tablets) followed by 250 mg q12h or placebo for a total duration of 7 days from the initiation of the infusion. Pediatric subjects aged 12 to 17 years will receive 400 mg (8 x 50 mg oral capsules) followed by 200 mg (4 x 50 mg oral capsules) q 12h. Pediatric subjects aged 5 to 11 years will receive 200 mg (4 x 50 mg oral capsules) followed by 100 mg (2 x 50 mg oral capsules) q 12h. Administration of the oral drug should occur within 15 minutes of stopping the IV infusion.

7.3 Dose Adjustment Criteria

Dose adjustment is not planned for this study.

7.4 Treatment Compliance

All hospitalized subjects will be administered the IP or placebo at the study site under the surveillance of appropriate study personnel until discharged from the hospital. Intravenous study drug and tablet administration details will be recorded by the site. Subjects who are discharged during the 7-day treatment period will be informed of the need for treatment compliance and asked to record the date and time of each dose taken at home.

7.5 Method of Assigning Subjects to Treatment Groups

In this parallel-group randomized study, subjects who meet study entry criteria will be randomly assigned in a 1:1 ratio to active study drug or placebo. The randomization schedule will be computer generated using a permuted block algorithm and will randomly allocate IP to randomization numbers. The randomization numbers will be assigned sequentially through a central interactive web response system (IWRS) as subjects are entered into the study. The randomization schedule will be stratified by snake type (copperhead, Mojave rattlesnake, non-Mojave rattlesnake, Russell's viper, Elapid, or other), and by age group: pediatrics aged 5 to 17 years or adults aged 18 years and older, resulting in 12 strata in total. No one involved in the study performance will have access to the randomization schedule before official unblinding of treatment assignment.

The randomization schedule will be prepared by the study contract research organization (CRO) before the start of the study. No subject will be randomized into this study more than once for a single snakebite.

7.6 Blinding and Unblinding Treatment Assignment

All participants, investigators, and study personnel involved in the conduct of the study, including data management, will be blinded to treatment assignment with the exception of (1) a specified unblinded statistician and programmer from the CRO who will have access to the randomization code, and the data safety monitoring board (DSMB), and (2) one secondary pharmacist at each site who will be unblinded to prepare IP. This unblinded pharmacist will have no other study participation or activities. Details are provided in the Pharmacy Manual. The unblinded study personnel will not participate in study procedures or data analysis prior to unblinding of the study data to all study-related personnel upon database lock.

Study personnel will make every effort to safeguard the integrity of the study blind to minimize bias in the conduct of the study. Treatment unblinding is discouraged if knowledge of the treatment assignment will not materially change the planned management of a medical emergency. Unblinding will be permitted in a medical emergency that requires immediate knowledge of the subject's treatment assignment.

Unblinding should be discussed in advance with the medical monitor, if possible. For emergency unblinding, study personnel will use the IWRS. If the Investigator is not able to

discuss treatment unblinding in advance, they must notify the medical monitor as soon as possible about the unblinding incident without revealing the subject's treatment assignment.

Unblinding for an individual subject will not result in unblinding the treatment assignments for the remaining subjects in the study. Thus, the overall study blind will not be compromised. If a subject's treatment assignment is unblinded, he/she may or may not be asked to withdraw from the study. The Investigator will make this decision after consultation with the medical monitor.

7.7 Prohibited and Permitted Therapies

All concomitant medications (including over-the-counter medications and herbal supplements as well as sedatives and paralytics for intubated patients) and concomitant therapies used will be recorded in the source document and on the appropriate eCRF.

7.7.1 Prohibited Therapies

The prohibited therapies prior to enrollment are described in Section 7.2.2.

7.7.2 Permitted Therapies

All medications and therapies for snakebite envenoming, including SOC, are allowed after enrollment in this study. No other experimental therapies are permitted during participation in this study.

Initial inhibition/induction potential of varespladib towards major human CYP450 enzymes have been performed. Data suggest that the active form of varespladib-methyl does not inhibit or induce major CYP450 enzymes. Although varespladib-methyl has been given to over 3,600 subjects with few AEs, limited *in vitro* data exists regarding the potential of varespladib to act as an inducer for P450-mediated metabolism or impact major human transporters. Therefore, the sponsor recommends avoiding use of CYP450 inducers and substrates or inhibitors of major human transporters (but their use is not prohibited) during the period of IP administration based on the Investigator assessment of risk. A list of transporters is included on the FDA.gov Drug Development and Drug Interactions website in Table 5-1 and 5-2 of the Table of Substrates, Inhibitors and Inducers document (FDA, 2020):

- Table 3-3, "Examples of clinical inducers for P450-mediated metabolisms (for concomitant use clinical DDI studies and/or drug labeling) (12/03/2019)"
- Table 5-1, "Examples of clinical substrates for transporters (for use in clinical drug-drug interaction [DDI] studies and/or drug labeling) (12/03/2019)"
- Table 5-2, "Examples of clinical inhibitors for transporters (for use in clinical drug-drug interaction [DDI] studies and drug labeling) (9/26/2016)."

7.8 Treatment After End of Study

After the end of the study, each subject will be treated according to standard clinical practice.

7.9 Dispensing and Storage

The IP and placebo supplied by Ophirex is to be used exclusively in the clinical study according to the instructions in this protocol. The Investigator is responsible for dispensing the IP and placebo according to the dosage scheme and for ensuring proper storage of the products.

Until the IP is dispensed to the subjects, it must be stored in a securely locked area that is not generally accessible. The vials of varespladib may be stored at room temperature. Saline may be stored at room temperature. The oral IP and placebo may be stored at room temperature. The key to the storage area is to be kept by the Investigator or designee responsible for the IP. The stored IP will be accessible only to those persons authorized by the Investigator to dispense the products.

Detailed instructions for selecting and dispensing each dose of IP and placebo are in the study manual.

7.10 Drug Accountability

The Investigator or designee must confirm the receipt of the IP with his or her signature. A copy of this receipt must be kept by the Investigator and another copy will be stored at Ophirex, Inc. or designee.

The Investigator or designee must maintain adequate records showing the receipt, dispensing, return, or other disposition of the IP, including the date, quantity, batch or code number, and identification of subjects who received the IPs. The Investigator will not supply the IP to any person except those named as sub-Investigators on the Food and Drug Administration (FDA) form 1572, designated study personnel, and subjects in this study. The Investigator will not dispense the IPs from any study sites other than those listed on the FDA Form 1572. IP may not be relabeled or reassigned for use by other subjects. If any of the IP is not dispensed, or is lost, stolen, spilled, unusable, or received in a damaged container, this information must be documented and reported to the sponsor and appropriate regulatory agencies, as required.

Upon completion of the study, the IP (partly used, unused, and empty packaging, e.g., vials and tablet bottles) must be left in the original packaging and destroyed on site.

7.11 Labeling and Packaging

Labeling and packaging of varespladib vials, oral varespladib-methyl, and placebo will be performed by Alcami, Inc., 1726 North 23rd Str, Wilmington, NC 28405. Over labeling of varespladib vials for shelf-life extension based on on-going stability will be accomplished by Sharp Clinical Services, Inc, 2400 Baglyos Circle, Bethlehem PA 18020. Additional investigational product oral varespladib-methyl, and placebo manufactured and packaged by Mikart will also be labeled by Sharp Clinical Services, Inc, 2400 Baglyos Circle, Bethlehem PA 18020.

7.11.1 Labeling

Kits will have labels affixed that meet the applicable regulatory requirements and may include the kit or randomization number, IP, dosage strength, blinded lot number, protocol number, volume or number of tablets, caution statement, storage, and sponsor identification.

8 STUDY PROCEDURES

In accordance with 10 USC 980, subjects must provide written informed consent and/or assent before any study-related procedures are initiated, including the cessation of prohibited concomitant therapy. As snakebites may constitute life-threatening medical conditions that necessitate urgent intervention, a legally authorized representative may provide consent for subjects who are not able to provide informed consent because of the severity of their condition. Patients who enter the study with consent from a legally authorized representative will be re-consented as soon as they have the capacity to make an informed, voluntary decision to participate in the trial.

An optional abbreviated consent may be used as the basis for randomization of patients but not for the basis of initiating the study drug. The abbreviated consent contains all the important elements of the full consent. No patient will receive any study drug until they or a legally authorized representative has signed the full consent.

For the timing of assessments and procedures throughout the study, refer to the Schedule of Events (Section 2.2). Throughout the study, every reasonable effort should be made by study personnel to follow the timing of assessments and procedures in the Schedule of Events for each subject. If a subject misses a study visit for any reason, the visit should be rescheduled as soon as possible. In the event that subjects are unable to respond to study visit assessments (i.e., CGI-I, PGIC, PSFS, NPRS, C-SSRS), data from those study visits will be handled as described in Section 9.3.1.

8.1 Study Duration

The overall study duration is expected to be approximately 24 months.

The sequence and duration of the study periods will be as follows:

1. Screening/Enrollment/Treatment Duration: 7 days. As subjects are admitted to ED, screening, enrollment, and treatment will take place on the same day.
2. Follow-up: approximately 21 days (28 days after initial study intervention).

8.2 Study Periods and Visits

8.2.1 sPLA$_2$ and Pharmacokinetics Sample Timing

Note that the sPLA$_2$ and PK sample collection will be performed with a different timing than the other assessments, as described in the Schedule of Events (Table 1). sPLA$_2$ samples and PK samples will be drawn at the same times.

Specifically, $sPLA_2$ and PK sampling will be performed for all subjects as described in Section 9.3.2.1.

8.2.2 Screening (Enrollment and Baseline)

Because patients are enrolled after a snakebite with envenoming, each subject must be screened, and treated within the first day (Day 1) of the study regardless of whether they have previously received antivenom or not. For the purpose of determining eligibility, snake type identification may be based on photographs, patient report, or clinician judgement based on symptoms and physical examination. Following completion of all assessments and confirmation of eligibility, the subject will be randomized via the IWRS.

Snakebite is an acute, life-threatening, and time-sensitive condition. In order to reduce the time to initiation of study drug, an optional abbreviated consent containing all elements of the full consent is available and may be used as the basis for randomization of patients. Once randomized, the research pharmacist can then prepare the study drug while the bedside clinical team reviews the full informed consent. Thus, the use of the abbreviated consent allows full consent and drug preparation to occur in parallel and so reduces time from consent to treatment and allows the study to better approximate the time frame for initiating the study drug if given as part of usual clinical care to victims of snakebite envenoming in hospitals in the U.S. and India. No patient will receive any study drug until they or a legally authorized representative has signed the full consent.

The following critical assessments/procedures must be performed at enrollment, prior to initiation of treatment: informed consent, inclusion/exclusion criteria review, medical history, physical examination, vital signs, SSS, Head-Lift, NPRS, CGI-I, and pre-dose laboratory blood draws.

The following noncritical assessments should be performed as early as possible, but may be performed after randomization and initiation of study drug and SOC if required due to patient condition: demographic information, height/body weight, 12-lead ECG, Grip strength, PGIC, CGI-S, PSFS, and C-SSRS.

The following procedures will be performed at Screening:

- Baseline:
  - Optional: Obtain informed consent for screening and randomization using the abbreviated consent. The abbreviated consent may be the basis for randomization but not the basis for initiating the study drug.
  - Obtain informed consent, either from the subject or a legally authorized representative
  - Review inclusion/exclusion criteria
  - Record prehospital tourniquet use as a snakebite treatment
  - Record hospitalization details
  - Record pulmonary support interventions

- Perform physical examination
- Measure vital signs per institutional practice
- Perform individual assessments pertaining to the SSS (A scoring sheet for use by study personnel is provided in Appendix A; clinical evaluation of the overall SSS [Appendix B] will be performed after completion of the study):
    - Local wound severity
    - Pulmonary severity
    - Cardiovascular severity
    - Gastrointestinal severity
    - Hematologic severity (do not wait for laboratory results)
    - Renal severity (do not wait for laboratory results)
    - Nervous system severity
- Perform baseline CGI-I compared to previous clinical status
- Measure Head-Lift duration (0 to 5 seconds)
- Perform NPRS
- Collect blood and urine for the following laboratory assessments:
    - CBC
    - Serum chemistry
    - CK
    - LFTs
    - Hemolysis (lactate dehydrogenase [LDH])
    - Prothrombin time (PT), partial thromboplastin time (PTT), thrombin time, INR
    - Absolute fibrinogen
    - Urine or serum pregnancy (subjects of childbearing potential only)
- Collect samples for PK and sPLA2 analysis from all subjects (see Section 9.2.1 and Section 9.3.2.1)
- Confirm and record details of snakebite envenoming including snake type.
  Obtain photographic documentation where feasible
- Assess and record demographic information (may be performed after randomization; treatment should not be delayed)
- Assess and record medical history, including time of bite, time and details of any previous treatment for envenoming (including antivenom and other), current medications (e.g., prescription and nonprescription medications) and therapies including antivenom use and analgesics. Other information here may be performed after randomization; treatment should not be delayed.

- Measure weight and height (may be performed after randomization; treatment should not be delayed)
- 12-lead ECG (may be performed after randomization; treatment should not be delayed)
- Urinalysis (may be performed after randomization; treatment should not be delayed)
- Serum pregnancy test, if not already obtained (subjects of childbearing potential only; may be performed after randomization; treatment should not be delayed)
- Measure grip strength
- Perform PGIC, PSFS, CGI-I and CGI-S (may be performed after randomization; treatment should not be delayed)
- Perform C-SSRS (Baseline/Screening Form; if subject is unable to respond, perform C-SSRS as early as clinically possible on Day 1, screening and randomization should not be delayed for C-SSRS performance)
- In sedated, endotracheally intubated subjects, perform additional assessments according to instructions in Section 9.3.1.7.
- Randomize subjects and assign IP to treatment and placebo arms

8.2.3 Treatment Period

The treatment period is defined as the time of first dose on Day 1 through Day 7. Treatment should be initiated as soon as possible after snakebite envenoming, and in conjunction with SOC.

The following procedures will be performed on Day 1 of the Treatment Period:

8.2.3.1 Day 1 (Dosing, 0 Hours)

- Administer IP (0.45 mg/kg/hr for adults, 0.60 mg/kg/hr for 5 – 17 year olds) continuous rate IV infusion) (Day 1, 0 Hours) in treatment and placebo arms
- Record time to SOC and IP intervention
- Record details of antivenom use
- Record hospitalization details
- Record pulmonary support interventions
- Record any concomitant therapy
- Assess and record any AEs

8.2.3.2 Day 1 (0.5 to 1 Hour)

- Continue IP infusion
- Collect PK and sPLA2 samples 0.5 hours post initiation of infusion

8.2.3.3 Day 1 (3 to 4 Hours)

- Continue IP infusion
- Measure vital signs per institutional practice
- Perform individual assessments pertaining to the SSS (clinical evaluation of the actual SSS point score will be performed after completion of the study):
  - Local wound severity
  - Pulmonary severity
  - Cardiovascular severity
  - Gastrointestinal severity
  - Hematologic severity
  - Renal severity
  - Nervous system severity
- Measure Head-Lift duration (0 to 5 seconds)
- Perform NPRS
- Measure grip strength
- Collect blood for the following laboratory assessments:
  - CBC
  - Serum chemistry
  - LFTs
  - Hemolysis (LDH)
  - PT, PTT, thrombin time, INR
  - Absolute fibrinogen
  - CK
- Record concomitant medications and therapies (e.g., prescription and nonprescription medications, pain medications)
- Perform PGIC
- Perform CGI-I

- In sedated, endotracheally intubated subjects, perform additional assessments according to instructions in Section 9.3.1.7.
- Record hospitalization details
- Record pulmonary support interventions
- Assess and Record AEs

8.2.3.4 Day 1 (5 to 6 Hours)

- Continue IP infusion
- Measure vital signs per institutional practice
- Perform individual assessments pertaining to the SSS (clinical evaluation of the actual SSS point score will be performed after completion of the study):
  - Local wound severity
  - Pulmonary severity
  - Cardiovascular severity
  - Gastrointestinal severity
  - Hematologic severity
  - Renal severity
  - Nervous system severity
- Measure Head-Lift duration (0 to 5 seconds)
- Perform NPRS
- Measure grip strength
- 12-lead EKG
- Collect blood for the following laboratory assessments:
  - CBC
  - Serum chemistry
  - LFTs
  - Hemolysis (LDH)
  - PT, PTT, thrombin time, INR
  - Absolute fibrinogen
  - CK
- Record concomitant medications and therapies (e.g., prescription and nonprescription medications, pain medications)

- Perform CGI-I
- Perform PGIC
- In sedated, endotracheally intubated subjects, perform additional assessments according to instructions in Section 9.3.1.7.
- Perform PSFS
- Collect PK and sPLA2 samples from subset of subjects as described in Section 9.2.1 and Section 9.3.2.1 (at end of infusion)
- Record hospitalization details
- Record pulmonary support interventions
- Assess and record AEs

8.2.3.5 Day 1 (6 Hours)

- Assess patient to determine if they meet clinical criteria for switching to the oral medication (see Section 6.3 for clinical assessment details).
    - Review recommended clinical criteria for stopping the IV infusion (Nervous System subscore ≤ 1, clinically stable per treating provider, and able to tolerate POs) and if indicated, stop infusion and initiate oral medication.
    - If patient does not meet criteria for switching to oral medication, continue infusion.

8.2.3.6 Day 2 (18-24 hours after the "Day 1, 6 hour" assessment)

- Continue either infusion or oral treatment with IP
- If receiving infusion, reassess patient every 12 hours to determine eligibility for transition to oral drug
- Perform physical examination
- Measure vital signs per institutional practice
- Perform individual assessments pertaining to the SSS (clinical evaluation of the actual SSS point score will be performed after completion of the study):
    - Local wound severity
    - Pulmonary severity
    - Cardiovascular severity
    - Gastrointestinal severity
    - Hematologic severity
    - Renal severity

  - Nervous system severity
- Measure Head-Lift duration (0 to 5 seconds)
- Perform NPRS
- Measure grip strength
- Collect blood and urine for the following laboratory assessments:
  - CBC
  - Serum chemistry
  - LFTs
  - Hemolysis (LDH)
  - PT, PTT, thrombin time, INR
  - Absolute fibrinogen
  - CK
  - Urinalysis
- Perform CGI-I
- Perform PGIC
- Perform PSFS
- Assess C-SSRS (Since Last Visit Form)
- Collect PK and sPLA2 samples from subset of subjects as described in Section 9.3.2.1
- In sedated, endotracheally intubated subjects, perform additional assessments according to instructions in Section 9.3.1.7
- Record concomitant medications and therapies (e.g., prescription and nonprescription medications, pain medications)
- Record hospitalization details
- Record pulmonary support interventions
- Assess and Record AEs

8.2.3.7 Day 3 (48 hours ±12 hours)

- Continue oral treatment with IP (subjects should transition to oral IP after 48 hours)
- Perform physical examination
- Measure vital signs per institutional practice
- Perform individual assessments pertaining the SSS (clinical evaluation of the actual SSS

point score will be performed after completion of the study):

- Local wound severity
- Pulmonary severity
- Cardiovascular severity
- Gastrointestinal severity
- Hematologic severity
- Renal severity
- Nervous system severity

- Measure Head-Lift duration (0 to 5 seconds)
- Perform NPRS
- Measure grip strength
- 12-lead EKG
- Collect blood and urine for the following laboratory assessments:
  - CBC
  - Serum chemistry
  - LFTs
  - Hemolysis (LDH)
  - PT, PTT, thrombin time, INR
  - Absolute fibrinogen
  - CK
  - Urinalysis
- Perform CGI-I
- Perform PGIC
- Perform PSFS
- Assess C-SSRS (Since Last Visit Form)
- In sedated, endotracheally intubated subjects, perform additional assessments according to instructions in Section 9.3.1.7
- Record concomitant medication and therapies (e.g., prescription and nonprescription medications, pain medications)
- Record hospitalization details
- Record pulmonary support interventions

- Assess and Record AEs

8.2.3.8 Day 7 (7 Days ± 1 Day)

- Continue oral treatment with IP
- If receiving infusion, reassess patient every 12 hours to determine eligibility to transition to oral drug
- Perform physical examination
- Measure vital signs per institutional practice
- Perform individual assessments pertaining to the SSS (clinical evaluation of the actual SSS point score will be performed after completion of the study):
  - Local wound severity
  - Pulmonary severity
  - Cardiovascular severity
  - Gastrointestinal severity
  - Hematologic severity
  - Renal severity
  - Nervous system severity
- Measure Head-Lift duration (0 to 5 seconds)
- Perform NPRS
- Measure grip strength
- 12-lead ECG
- Collect blood and urine for the following laboratory assessments:
  - CBC
  - Serum chemistry
  - LFTs
  - PT, PTT, thrombin time, INR
  - Absolute fibrinogen
  - Urinalysis
- Collect PK and sPLA2 samples from all subjects as described in Section 9.2.1 and Section 9.3.2.1
- Perform CGI-I
- Perform PGIC

- Perform CGI-ER
- Perform PSFS
- Assess C-SSRS (Since Last Visit Form)
- In sedated, endotracheally intubated subjects, perform additional assessments according to instructions in Section 9.3.1.7
- Record concomitant medications and therapies (e.g., prescription and nonprescription medications, pain medications)
- Record hospitalization details
- Record pulmonary support interventions
- Assess and Record AEs

8.2.3.9 Day 14 (14 Days ± 2 Days)

- Perform physical examination
- Measure vital signs per institutional practice
- Perform individual assessments pertaining to the SSS (clinical evaluation of the actual SSS point score will be performed after completion of the study).
  - Local wound severity
  - Pulmonary severity
  - Cardiovascular severity
  - Gastrointestinal severity
  - Hematologic severity
  - Renal severity
  - Nervous system severity
- Measure Head-Lift duration (0 to 5 seconds)
- Perform NPRS
- Measure grip strength
- Collect blood and urine for the following laboratory assessments:
  - CBC
  - Serum chemistry
  - PT, PTT, thrombin time, INR
  - Absolute fibrinogen

- Perform CGI-I
- Perform PGIC
- Perform PSFS
- Assess C-SSRS (Since Last Visit Form)
- Record medical history, including current medications and therapies (e.g., prescription and nonprescription medications, pain medications)
- Record hospitalization details
- Record pulmonary support interventions
- Assess and Record AEs

8.2.3.10 Day 28 (28 ± 3 Days) (End of Study Visit)

- Perform physical examination
- Measure vital signs per institutional practice
- Measure body weight
- Perform individual assessments pertaining to the SSS (clinical evaluation of the actual SSS point score will be performed after completion of the study):
  - Local wound severity
  - Pulmonary severity
  - Cardiovascular severity
  - Gastrointestinal severity
  - Hematologic severity
  - Renal severity
  - Nervous system severity
- Measure Head-Lift duration (0 to 5 seconds)
- Perform NPRS
- Measure grip strength
- 12-lead ECG
- Collect blood and urine for the following laboratory assessments:
  - CBC
  - PT, PTT, thrombin time, INR
  - Absolute fibrinogen

- Serum chemistry
- LFTs
- Urinalysis
- Serum pregnancy test (subjects of childbearing potential only)

- Perform CGI-I
- Perform PSFS
- Perform PGIC
- Assess C-SSRS (Since Last Visit Form)
- Record concomitant medications and therapies (e.g., prescription and nonprescription medications, pain medications)
- Record hospitalization details
- Record pulmonary support interventions
- Assess and Record AEs

8.3 Assessments

8.3.1 Efficacy Assessments

Assessments used in this protocol include additional bedside assessment methods for endotracheally intubated subjects, the SSS, the CGI-I, the PGIC, the PSFS, the NPRS, grip strength, Head-Lift duration, and analgesic use. Each study subject will be evaluated using the PGIC, PSFS, grip strength, and NPRS at the timepoints indicated in the Schedule of Events (Table 1). The SSS evaluation is provided as a checklist in Appendix A and B; all other forms and assessments are provided in Appendix C.

Acute changes in a patient's clinical status that occur during the first 24 hours, including the period between enrollment and administration of IP, but outside of a "Scheduled Assessment" timepoint should be recorded as "Unscheduled Visit" in EDC systems, as appropriate.

Individuals that cannot respond to assessments due to severity of snakebite envenoming will be given a score corresponding to the worst score available in the assessment. Specific assessments and unable to respond scores are:

- PSFS = 0 (Unable to perform activity)
- PGIC =10 (Feel much worse)
- Grip Strength = 0
- 5-second head-lift = 0 seconds

8.3.1.1 Snakebite Severity Score

The SSS (Dart RC et al., 1996) is a tool that was developed to measure the severity of envenoming based on 6 body system categories. The composite SSS score to be used, herein, is based on 7 categories and includes respiratory paralysis, acute renal failure, major thrombotic events, and death.

The renal system category will be used to characterize the severity of acute renal injury (scoring provided in Appendix B). The baseline value for serum creatinine will be defined as the most recent serum creatinine in the past year, if one is available, or as the normal expected value for the patient.

Severe weakness or paralysis, including respiratory paralysis, will be scored a 3 in the nervous system section. Minimal, moderate, and severe weakness will be differentiated based on the presence of objective findings of weakness using available tools that include but are not limited to physical examination, physiologic testing or neuromonitoring, alone or in combination as available within the local standard of care.

Major thrombotic events will be scored in the appropriate SSS category as clinically presented.

Death will be scored as the maximum score in each section.

The 7 body systems are included as follows:

- Local wound (e.g., pain, swelling and ecchymosis)
- Pulmonary
- Cardiovascular
- Gastrointestinal
- Hematologic
- Nervous system
- Renal

Each of these 7 categories are graded at levels from Grade 0 to Grade 3 (pulmonary, cardiovascular, gastrointestinal, nervous, and renal systems) or Grade 0 to Grade 4 (local wound and hematologic symptoms). A higher score indicates worse signs or symptoms. SSS scores will be performed by study personnel using the scoring sheet provided in Appendix B. At each assessment timepoint, each of the 7 SSS subscores should be recorded and a justification for each score should be provided.

For the primary endpoint, patients bitten by vipers will be assessed using an abbreviated SSS that is the sum the local wound, hematologic, and nervous system subscores. The composite SSS, which is the basis for secondary outcomes, is the sum of the local wound, pulmonary, cardiovascular, hematologic, nervous system, and renal subscores.

8.3.1.2 Clinical Global Impression-Improvement

Efficacy determined using a CGI-I of efficacy (Appendix C1). The CGI-I at baseline should be determined using all available information, such as interviews with patients and family members, reports from other providers (including prehospital and transfer reports), medical records, and prior laboratory data (Busner and Targum, 2007) (Appendix C1). Baseline rating should be in comparison to previous clinical status using all available information. All post-baseline CGI-I assessments should be compared to the patient's condition at baseline (e.g., time of enrollment).

The CGI-I is defined as a selection of one of the following at each assessment period:

1 = very much improved since the initiation of treatment
2 = much improved
3 = minimally improved
4 = no change from baseline (the initiation of treatment)
5 = minimally worse
6 = much worse
7 = very much worse since the initiation of treatment

The Clinical Global Impression-Severity of Illness (CGI-S) Scale (Appendix C2) is also assessed at baseline but not at any other time points. It is not an assessment of efficacy. Rather, it is a baseline assessment that can be used to assist in the interpretation and statistical adjustment of efficacy outcomes.

8.3.1.2.1 Clinical Global Impression-Expected Recovery (CGI-ER)

This single question at Day 7 obtains a clinician assessment as to whether the patient's clinical course over the first week is better than expected, worse than expected, or as expected. For patients whose outcome is either better or worse than expected, we ask clinicians to describe the basis for this assessment.

8.3.1.3 Patient Global Impression of Change Scale (PGIC)

The PGIC (Appendix C3) is a short, widely used, qualitative metric to evaluate a subject's belief regarding the efficacy of treatment. This metric consists of a question asking the subject to score their overall change in symptoms, followed by a question regarding the degree of change.

8.3.1.4 Patient-Specific Functional Scale

The PSFS (Appendix C4) is a questionnaire that can be used to quantify activity limitation and measure functional outcome for patients. The clinician asks each subject to identify up to 3 activities that they are unable to perform due to their medical condition, and asks the subject to rate their ability to perform that activity on a scale from 0 (unable to perform) to 10 (able to perform to the same ability as before the medical condition). Analyses will be based on the average of the scores provided at each time point.

8.3.1.5 Numeric Pain Rating Scale

The NPRS is a numerical metric that is used by patients to evaluate pain they are experiencing. (Appendix C5).

8.3.1.6 Columbia-Suicide Severity Rating Scale

The C-SSRS is a short questionnaire, available in 114 languages, that is designed to provide suicide risk assessment. Ultimately, the determination of the presence of suicidal ideation or behavior depends on the judgment of the individual administering the scale and patients indicating ideation of self-harm at any point in the study should be evaluated and treated per institutional practice.

Generally, the C-SSRS should be administered at Baseline, or at the earliest time point clinically allowable (ideally on Day 1) and then at every study visit through Day 28. For the sake of this clinical study, modified versions of the C-SSRS will be administered at different study visits to minimize burden on study subjects. The modified C-SSRS forms are provided in Appendix C6 as the "C-SSRS Baseline/Screening Form" and the "C-SSRS Since Last Visit Form." The C-SSRS will not be administered to patients under 8 years of age.

8.3.1.7 Additional Assessments on Endotracheally Intubated Subjects

For SSS nervous system examination in endotracheally intubated patients:

1. Record current mechanical ventilation modes and all relevant ventilator settings; e.g., control mode ventilation, weaning mode ventilation, or independent breathing without any support. Commonly used ventilatory modes are listed in Appendix C8.
2. Remove sedation (*and* if applicable, paralytics) sufficiently early for awake examination (e.g., until patient is sufficiently awake to follow commands) per institutional practice.
3. Record SSS and Head-Lift once patients is awake and alert or at the 60-minute point after discontinuation of sedation, whichever comes first. If patients is receiving paralytics, these should also be stopped to ensure they do not interfere with assessments.
4. Record time from removal of sedation to examination and describe the level of alertness/sedation per institutional practice. If a sedation score is typically used, it should be recorded and the scoring system used should be specified.
5. In subjects who meet institutional weaning criteria, record time of weaning initiation, and mode and settings of mechanical ventilation as listed in Appendix C8 to indicate the level of support/independence to which they have progressed.
    a. Time of initiation of weaning should be documented by the investigator or clinical provider. Criteria for initiation of weaning should follow institutional practice but may consider the following factors: neuromuscular power; triggered breathing ≥ 12 per minute: systolic blood pressure ≥ 90 mmHg; Glasgow coma scale > 13 (Sehgal et al., 2022).
6. In subjects who meet institutional criteria for immediate extubation but deferred or postponed as per institutional practice (e.g., meets criteria for extubation at night but not

extubated until the next day), note patient status and record mode and settings of mechanical ventilation (see Appendix C8). For all subjects who receive intubation and ventilation, collect and record an additional complete SSS within 6 hours of extubation, unless an SSS has been obtained as part of routine assessments during this post-extubation time period.

7. If used per institutional practice, record negative inspiratory force (NIF)/maximum inspiratory pressure (MIP).

8.3.1.8 Head-Lift Duration

Patient should be supine on flat bed with no pillow. Ask patient to lift their head approximately 8 cm off of bed. Measure the duration in seconds that the patient can hold their head approximately 8 cm off of the bed. Stop after 5 seconds (i.e., maximum duration is 5 seconds).

8.3.1.9 Grip Strength

Grip strength will be evaluated using a dynamometer, as a measure of the severity of the neurological effects of snakebite. Grip strength should be measured in the dominant hand, or in the non-dominant hand if the bite occurred on dominant hand. For each assessment time point, all 3 values will be recorded, but analyses will be based on the highest value.

8.3.1.10 Analgesic Use

For each assessment time point, the patient's current analgesic use will be characterized by the clinical research team based on the strongest category of analgesic taken to treat snakebite-related pain.

8.3.2 Pharmacokinetics

8.3.2.1 Pharmacokinetic Analysis Methods

Testing of PK parameters will be outlined in the SAP. PK parameters of varespladib in plasma from all study subjects using the schedule presented in Table 4.

Table 4 Pharmacokinetic Sampling Schedule

IV	Oral<br>1st dose (500mg adult, 400 mg / 200 mg pediatric)	Oral<br>2nd dose (250mg adult, 200 mg / 100 mg pediatric)	Day 7 (Oral)
• Pre-dose<br>• 0.5h post initiation<br>• End of Infusion	• 2 hours after first oral dose<br>• Either 5-10 hours after first oral dose OR after 2nd, 3rd, or 4th in-hospital oral dose	• **If hospitalized**<br>Sample prior to second oral dose<br>OR<br>• **If discharged prior to second dose:**<br>Sample prior to discharge	• Pre-Dose

Population PK (PopPK) analysis will be conducted using the PK data collected in this study, as well as the data obtained from the OPX-PR-01 study. Additional details of the parameters and their calculation and evaluation will be included in a separate population PK analysis plan.

8.3.2.2 Pharmacokinetic Parameters

Standard PopPK methods model clearance (CL) and volume of distribution (Vd) of the central compartment. Additional PK parameters and covariates will be derived as data permit.

8.3.2.3 Sample Collection

Samples will be collected at the time points specified in Section 9.3.2.1 and the Schedule of Events (Section 2.2).

Blood

The total amount of blood to be collected will include samples for CBC, renal function tests, liver function test, coagulation profile, and biomarkers, $sPLA_2$ levels, and plasma drug levels.

Urine

Mid-stream or catheter urine collected for dipstick urinalysis. Urinalysis should not delay care and should not be obtained by catheter unless patient already being catheterized. Urine should not be collected from a bag, but rather from proximal port.

8.3.3 Safety Variables

Safety will be assessed by evaluating AEs, vital sign measurements, clinical laboratory test results, 12-lead ECGs, physical examination findings, and concomitant medications and therapies.

8.3.3.1 Clinical Laboratory Safety Assessments

8.3.3.1.1 Clinical Laboratory Tests to be Performed

Samples for the following laboratory tests will be collected at the time points indicated in the Schedule of Events (Table 1).

Complete blood count (CBC):	White blood cell count including differential, hemoglobin/hematocrit, platelets
Liver function:	Albumin, total bilirubin, alkaline phosphatase, alanine aminotransferase, aspartate aminotransferase, LDH
Serum chemistry:	Sodium, potassium, chloride, bicarbonate, creatinine, BUN, eGFR
Hemolysis	LDH
Myonecrosis	CK
Coagulation:	PT, PTT, absolute fibrinogen, INR
Urinalysis:	pH, specific gravity, blood, glucose, protein, ketones
Other:	$sPLA_2$ activity, PK
Serum pregnancy test:	evaluated for subjects of childbearing potential only

Routine laboratory specimens will be analyzed at local laboratories, as specified in the study laboratory manual. A central laboratory will be used for nonstandard assays.

8.3.3.1.2 Evaluation of Clinical Laboratory Values

Each local laboratory will provide the normal ranges of values for the clinical laboratory assessments in this study, and the local ranges will be regarded as the reference ranges on which decisions will be made by each Investigator.

If a laboratory value is out of the reference range, it is not necessarily clinically relevant. The Investigator must evaluate the out-of-range values and record his or her assessment of the clinical relevance in the appropriate eCRF.

All clinical laboratory values that in the Investigator's opinion show clinically relevant or pathological changes during or after termination of treatment must be reported as AEs and followed, as described in Section 10.2.6.

8.3.3.2 Clinical Examinations

8.3.3.2.1 Vital Signs

Vital signs will be assessed and recorded at the timepoints indicated in the Schedule of Events (Table 1) per institutional practice. In addition, body temperature will be measured and recorded every 4 to 8 hours during hospitalization.

8.3.3.2.2 Twelve-lead Electrocardiogram

A standard 12-lead ECG will be performed at the timepoints indicated in the Schedule of Events (Table 1) per institutional practice. All ECG recordings will be identified with the subject identification number, date, study timepoint, and time of the recording and will be attached to the subject's eCRF.

8.3.3.2.3 Physical Examination

A physical examination will be performed at the timepoints indicated in the Schedule of Events (Table 1) per institutional practice.

8.3.3.2.4 Neurological Assessment

For sedated, endotracheally intubated subjects, a separate neurological assessment will be performed according to Section 9.3.1.7.

8.3.3.3 Adverse Events

The definitions and management of AEs, and any special considerations for AEs, are provided in Section 10.

9 ADVERSE EVENTS

9.1 Definitions

9.1.1 Adverse Events

An AE is defined as any untoward medical occurrence in a patient or clinical investigation subject administered an IP that does not necessarily have a causal relationship with this treatment. An AE can therefore be any unfavorable and unintended sign (including an abnormal laboratory finding, for example), symptom, or disease temporally associated with the use of a medicinal product, whether related to the medicinal product, or not.

Pre-existing diseases or conditions will **not** be considered AEs unless there is an increase in the frequency or severity, or a change in the quality, of the disease or condition. Worsening of a pre-existing condition is considered an AE. Abnormal laboratory values related to liver and renal function that are deemed clinically significant by the investigator will always be considered as AEs if they have worsened from baseline. Any other laboratory values that are judged by the site investigator to be wholly caused by the snakebite envenoming will be regarded as AEs at the discretion of the investigator.

Events that occur in subjects treated with control product (SOC), or during treatment-free periods of the study, are also considered AEs.

9.1.2 Unexpected Adverse Event

An expected AE is one for which the nature or severity is consistent with the known AE profile of the product. For a pre-approval test product, the known information is contained in the IB. For a marketed product, the known information is contained in the current package insert for the product.

An unexpected adverse event (UAE) is one for which the nature or severity of which is not consistent with the applicable product information (e.g., IB for an unapproved IP or package insert/summary of product characteristics for an approved product). For example, hepatic necrosis would be unexpected (greater severity) if the IB only listed elevated hepatic enzymes or hepatitis. Likewise, cerebral vasculitis would be unexpected (greater specificity) if the IB only listed cerebral vascular accidents.

Furthermore, reports that add significant information on specificity or severity of a known, already documented adverse reaction constitute unexpected events. Examples would be (a) acute renal failure as an expected adverse reaction with a subsequent new occurrence of interstitial nephritis (interstitial nephritis would be unexpected) and (b) hepatitis with a first occurrence of fulminant hepatitis (fulminant hepatitis would be unexpected.)

9.1.3 Serious Adverse Events

An SAE is any untoward medical occurrence that at any dose:

- Results in death
- Is life-threatening
  NOTE: The term "life-threatening" in the definition of "serious" refers to an event in which the patient was at risk of death at the time of the event; it does not refer to an event that hypothetically might have caused death if it were more severe.
- Requires inpatient hospitalization or prolongation of existing hospitalization
  An elective hospital admission to treat a condition present before exposure to the IP, or a hospital admission for a diagnostic evaluation of an AE, does not qualify the condition or event as an SAE.
- Results in persistent or significant disability/incapacity
- Is a congenital anomaly
  NOTE: A congenital anomaly in an infant born to a mother who was exposed to the IP during pregnancy is an SAE. However, a newly diagnosed pregnancy in a subject that has received an IP is not considered an SAE unless it is suspected that the IP(s) interacted with a contraceptive method and led to the pregnancy.
- Is an important medical event
  NOTE: Medical and scientific judgment should be exercised in deciding whether it is appropriate to consider other situations serious, such as important medical events that may not be immediately life-threatening or result in death or hospitalization but may jeopardize the patient or may require intervention to prevent one of the other outcomes listed in the definition above. Examples of such events are intensive treatment in an emergency room or at home for allergic bronchospasm, blood dyscrasias, or convulsions that do not result in hospitalization, development of drug dependency, or drug abuse.

9.1.4 Treatment-Emergent Adverse Events

An AE is defined as treatment-emergent if the first onset or worsening is during or after the first administration of IP and not more than 30 days after the last administration of IP.

9.2 Event Assessment and Follow-Up of Adverse Events

The occurrence of an AE or SAE may come to the attention of study personnel during study visits and interviews of a study subject presenting for medical care or upon review by a study monitor.

All AEs including local and systemic reactions not meeting the criteria for SAEs will be captured on the appropriate CRF. Information to be collected includes event description, time of onset, clinician's assessment of severity, relationship to study product (assessed only by those with the training and authority to make a diagnosis), and time of resolution/stabilization of the event. All AEs occurring while on study must be documented appropriately regardless of relationship. All AEs will be followed to adequate resolution or stabilization.

Any medical condition that is present at the time that the subject is screened will be considered as Baseline and not reported as an AE. However, if the study subject's condition deteriorates at any time during the study, it will be recorded as an AE.

Changes in the severity of an AE, or an AE that is intermittent, will be documented as separate AEs to allow an assessment of the duration of the event at each level of severity to be performed. AEs characterized as intermittent require documentation of onset and duration of each episode.

Site staff will record all reportable AEs with start dates occurring any time after informed consent is obtained until 28 days after the last day of study participation. At each study visit, the Investigator will inquire about the occurrence of AE/SAEs since the last visit.

9.2.1 Assessment

The Investigator is responsible for the detection and documentation of events meeting the criteria and definition of an AE or SAE described previously. At each visit, the subject will be allowed time to spontaneously report any issues since the last visit or evaluation. The Investigator will then monitor and/or ask about or evaluate AEs using nonleading questions, such as

- "How are you feeling?"
- "Have you experienced any issues since your last visit?"
- "Have you taken any new medications since your last visit?"

Any clinically relevant observations made during the visit will also be considered AEs.

9.2.2 Evaluation

9.2.2.1 Severity of Adverse Events

The clinical severity of an AE will be classified as:

Mild	Usually transient and may require only minimal treatment or therapeutic intervention. The event does not generally interfere with usual activities of daily living.
Moderate	Usually alleviated with additional specific therapeutic intervention. The event interferes with usual activities of daily living, causing discomfort but poses no significant or permanent risk of harm to the subject.
Severe	Interrupts usual activities of daily living, or significantly affects clinical status, or may require intensive therapeutic intervention.

It is important to distinguish between severe AEs and SAEs. Severity is a classification of intensity whereas an SAE is an AE that meets serious criteria, as described in Section 10.1.3.

9.2.2.2 Seriousness

The Investigator is to evaluate whether the AE meets serious criteria, as described in Section 10.1.3.

9.2.2.3 Action(s) Taken

Action(s) taken may consist of:

IP interrupted	IP schedule was modified by temporarily terminating the prescribed regimen of IP.
IP withdrawn	IP schedule was modified through termination of IP.
Not applicable	Determination of a value is not relevant in the current context.
Unknown	Not known, not observed, not recorded, or refused.

9.2.2.4 Outcome at the Time of Last Observation

The outcome at the time of last observation will be classified as:

- Recovered/resolved
- Recovered/resolved with sequelae
- Recovering/resolving
- Not recovered/not resolved
- Fatal*

- Unknown

*Fatal should only be selected as an outcome when the AE results in death. If more than one AE is judged to be possibly related to the subject's death, the outcome of death should be indicated for each such AE. Although "fatal" is usually an event outcome, events such as sudden death or unexplained death should be reported as SAEs.

9.2.2.5 Adverse Event Relationship to Investigational Product

The Investigator must assess each AE's relationship to the IP, to antivenom, and to the snakebite. The Investigator must carefully consider possible manifestations of snakebite when assigning a relationship of specific AEs to study drug. The categories for classifying the Investigator's opinion of the relationships are as follows:

Definitely	A definite probability exists of a relationship between the AE and IP.
Probably	A reasonable probability exists of a relationship between the AE and IP.
Possibly	A possibility exists of a relationship between the AE and IP.
Unrelated	No reasonable possibility exists of a relationship between the AE and IP

9.2.3 Documentation

All AEs that occur within the period of observation for the study must be documented in the CRF with the following information, where appropriate. (The period of observation for the study is described in Section 10.2.)

- AE name or term
- When the AE first occurred (start date and time)
- When the AE stopped (stop date and time or an indication of "ongoing")
- Severity of the AE
- Seriousness (hospitalization, death, etc.)
- Actions taken
- Outcome
- Investigator opinion regarding the AE relationship to the IP

9.2.4 Treatment of Adverse Events

AEs that occur during the study will be treated, if necessary, by established standards of care. If such treatment constitutes a deviation from the protocol, the decision about whether the subject may continue in the study will be made by the sponsor after consultation with the Investigator and/or medical monitor.

If AEs occur in a subject that are not tolerable, the Investigator must decide whether to stop the subject's involvement in the study and/or treat the subject. Special procedures may be recommended for the specific IP, such as the collection of a serum sample for determining blood concentrations of IP, specific tapering procedures, or treatment regimens, as appropriate.

For double-blinded studies, it is not necessary to unblind a subject's treatment assignment in most circumstances, even if an SAE has occurred. If unblinding is necessary, see Section 8.6 for a description of the unblinding procedures.

9.2.5 Follow-Up

Any AE will be followed (up to a maximum of 30 days after the last dose of IP) to a satisfactory resolution, until it becomes stable, or until it can be explained by another known cause(s) (i.e., concurrent condition or medication) and clinical judgment indicates that further evaluation is not warranted. All findings relevant to the final outcome of an AE must be reported in the subject's medical record and recorded on the eCRF page.

9.2.6 Reporting

9.2.6.1 Serious Adverse Events

The Investigator or designee must report all SAEs promptly to the study CRO within 24 hours of first becoming aware of the event by completing, signing and dating the SAE Report Form, verifying the accuracy of the information recorded in the form with the source documents and eCRF, and sending the SAE form to the study CRO. All SAEs from US and India should be sent to: Safetyreporting@syneoshealth.com. This written report should be submitted on the SAE form provided for this purpose. At the time of first notification, the Investigator or designee should provide the following information, if available:

- Protocol number
- Reporter (study site and Investigator)
- Subject's study number
- Subject's year of birth
- Subject's sex
- Date of first dose of IP(s)
- Date of last dose of IP(s), if applicable
- Adverse event term
- Date of occurrence of the event
- A brief description of the event, outcome to date, and any actions taken

- The seriousness criteria(on) that were met
- Concomitant medication at onset of the event
- Relevant medical history information
- Relevant laboratory test findings
- Investigator's opinion of the relationship to IP(s) ("Is there a reasonable possibility that the IP caused the SAE? Yes or No?")
- Whether and when the Investigator was unblinded as to the subject's treatment assignment

Any missing or additional relevant follow-up information concerning the SAE should be sent to the sponsor/sponsor representative via the same contact details above as soon as possible on a follow-up SAE Report Form, together with the following minimal information (initial report, AEs, date of occurrence, subject identification (ID), study ID, IP, and site number); this will allow the follow-up information to be linked to the initial SAE report.

Specific information may be requested by the study CRO using a follow-up request form or via email communication.

The Investigator is required to comply with applicable regulations (including local laws and guidances) regarding the notification of his or her health authorities, institutional review board (IRB), principal and coordinating Investigators, study Investigators, and institutions. Each Investigator is obligated to learn about the reporting requirements for Investigators in his/her country. The study monitor can assist with this.

9.3 Special Considerations

9.3.1 Adverse Events of Special Interest

Investigators should specifically monitor several AEs associated with varespladib treatment, including cerebrovascular accident (CVA; stroke), MI, and malignant cardiac arrhythmias. However, these AEs may also be associated with many snakebites.

9.3.2 Pregnancy

All subjects of childbearing potential who participate in the study should be counseled on the need to practice adequate birth control and on the importance of avoiding pregnancy during treatment. Subjects should be instructed to contact the Investigator or study staff immediately if pregnancy occurs or is suspected.

Participants of reproductive potential must agree to use a highly effective method of contraception from the time of consent and for 14 days following his or her last dose of IP. Highly effective methods of contraception are as follows:

- combined (estrogen- and progestogen-containing) hormonal contraception associated with inhibition of ovulation (oral, intravaginal, or transdermal)

- progestogen-only hormonal contraception associated with inhibition of ovulation (oral, injectable, or implantable)
- intrauterine device, intrauterine hormone-releasing system
- bilateral tubal occlusion
- vasectomized partner
- sexual abstinence
- double-barrier method (condoms, sponge, diaphragm, with spermicidal jellies, or cream)

Pregnancy testing will be conducted prior to administration of the IP on every subject of childbearing potential. A subject who is found to be pregnant at the Screening Visit will be excluded from the study and considered to be a screening failure.

A subject who becomes pregnant during IP treatment will be immediately discontinued from receiving the study drug. The Investigator must report the pregnancy within 72 hours of learning of the pregnancy, to the study CRO pharmacovigilance team using the Pregnancy Data Collection Form via the same fax number and/or email address as for SAE reporting. The Investigator should contact the designated individual(s) who receive SAE notification and record information related to the pregnancy on a pregnancy form provided by the sponsor or its designee.

The Investigator is also responsible for following the pregnancy until delivery or termination. These findings must be reported on a pregnancy form and forwarded to the designated individual(s). The event meets the SAE criterion only if it results in a spontaneous abortion or a congenital anomaly.

9.3.3 Overdosage

As with any overdose, patients should be monitored closely and observed for expected and unexpected clinical and laboratory effects.

No specific antidote to varespladib or varespladib-methyl exists, so treatment should be directed at supportive care.

In the presence of normal hepatic and renal function, blood levels of varespladib would be expected to rapidly diminish. Clinical and laboratory effects of varespladib overdose, however, may be delayed and patients should be monitored until all such effects have resolved.

Based on preclinical toxicology studies, acute renal failure may result from high plasma levels of varespladib. Renal toxicity studies performed in rats suggest that forced diuresis may diminish the extent of renal injury following bolus administration of varespladib-methyl. No data in humans are available to support forced diuresis as a potential therapy. However, as the toxicity of many potential nephrotoxins can be diminished by increased urine flow rates, fluid or mannitol administration to promote diuresis may be reasonable in the setting of

varespladib or varespladib-methyl overdose. In circulation, varespladib is highly (approximately 90%) protein bound. Studies to determine if hemodialysis or hemoperfusion would be of benefit in patients with varespladib-methyl overdose have not been performed.

10 DATA SAFETY MONITORING BOARD

The DSMB will operate under a charter that will be finalized prior to the start of the study. The DSMB will evaluate the safety data, including summaries by group, at the intervals specified in the DSMB charter. In case of significant toxicity, the DSMB may choose to review the available safety data and recommend stopping recruitment in a particular dose group. If toxicity is observed, the treatment assignment for the subject(s) involved may be unblinded by the DSMB at its discretion. The DSMB will also review blinded Snakebite Severity Scores and generate queries as needed to ensure consistency of scoring across sites, using objective and standardized measures as available.

11 STATISTICS

11.1 Statistical Analysis

This section is a summary of the planned statistical analyses of the primary and secondary endpoints.

Descriptive statistical methods will be used to summarize the data from this study with confidence intervals calculated for the primary and secondary efficacy endpoints. Unless stated otherwise, the term "descriptive statistics" refers to the number of subjects (n), mean, median, standard deviation (SD), minimum, and maximum for continuous data; and frequencies and proportions for categorical data. All data collected during the study will be included in data listings.

P-values of less than 0.05 will be considered statistically significant based on a 2-sided test unless otherwise specified.

All statistical analyses will be conducted with the SAS® System, version 9.3 or higher.

11.1.1 Analysis Populations

The following analysis populations are planned for this study:

- **Safety Population (SAF):** The Safety Population includes all subjects who receive any amount of IP. Subjects who are members of the SAF population will be analyzed according to the treatment received. This population will be used for analysis of demographics, baseline characteristics, and all safety analyses.
- **Intent-To-Treat Population (ITT):** The ITT population includes all randomized subjects who have signed the full consent form. The ITT population will not include subjects who sign the abbreviated consent, are randomized, but decline to sign the full consent. Note: As a blinded trial, there is no mechanism by which the patient or clinical

team would be able to know the treatment assignment, so any decisions made by patients not to sign the full consent would be made independent of treatment arm.

Subjects included in the ITT population will be analyzed as randomized. The ITT population will be used for analysis of demographics, baseline characteristics, and all efficacy analyses.

- **Per-Protocol Population (PP):** The PP population includes all subjects in the ITT without any significant protocol deviation. Members of the PP population will be analyzed as randomized. This will be a supporting population for the primary efficacy analysis.
- **Pharmacokinetic Population (PK):** The PK Population includes all subjects in the SAF Population who provide at least 1 evaluable post-dose PK measurement. Subjects who are members of the PK population will be analyzed according to the treatment received.

Assignment of subjects to populations will be confirmed at a blinded data review meeting to be held before the study database is locked.

11.1.2 Study Subjects and Demographics

11.1.2.1 Disposition and Withdrawals

The numbers of subjects randomized, completing, and withdrawing, along with reasons for withdrawal, will be tabulated overall and by treatment group. The number of subjects in each analysis population will be reported.

11.1.2.2 Protocol Deviations

Protocol deviations will be identified and classified as important or not important for statistical analysis purposes before unblinding and will be summarized or listed as appropriate. Key protocol deviations will be used to exclude subjects from the PP population. Due to the Coronavirus Disease 2019 (COVID-19) global health crisis, study visits and protocol-defined procedures may be impacted; COVID-19-related protocol deviations are discussed in Appendix D.

11.1.2.3 Demographics and Other Baseline Characteristics

These analyses will be conducted for the Safety and ITT populations.

Demographic variables will include age, sex, height, and weight. Information on race and ethnicity will be collected for any eventual analysis of differences in response to the IP, in accordance with local regulatory requirements. Baseline subject characteristics will include medical history and physical examination findings.

Prior and concomitant medications will be summarized by treatment group, by the number and percentage of subjects taking each medication, classified using World Health Organization Drug Dictionary (WHO-DD) Anatomical Therapeutic Chemical classes and preferred terms.

11.1.3 Exposure and Compliance

IP exposure and dosing information including the number of doses and amount of IP received will be listed for each subject.

11.1.4 Efficacy Analysis

11.1.4.1 Primary Efficacy Endpoints

Statistical analysis of efficacy will be assessed (1) in elapids using time to complete recovery defined by a 5-second head-lift of 5 seconds and (2) in vipers using an area under the curve of an abbreviated SSS composed of Local Wound, Hematologic and Nervous System subscores from Baseline to Day 14. The p-values obtained from these two analyses of independent subgroups of patients will be tested at a 2-sided alpha level of 5%. These results will then be combined using Fisher's combined probability test, and a global p-value for the primary endpoint will be calculated. An overall benefit will only be declared if the p-values for both the viper and elapid groups directionally favor treatment with varespladib. Scoring for patients who die will be specified in the SAP. Missing measurements of the SSS will be imputed following the methods defined in the SAP. Additional details regarding the primary endpoint analysis is provided in the SAP.

11.1.4.2 Secondary Efficacy Endpoints

The secondary efficacy endpoints are listed below. Alpha protected endpoints will be specified in the SAP.

- Area under the curve of the composite outcome of the pulmonary, cardiovascular, local wound, hematologic, renal, and nervous system sections of the snakebite severity score (SSS) from Baseline (pre-dosing) to Day 7.
- Area under the curve of the composite outcome of pulmonary, cardiovascular, local wound, hematologic, renal, and nervous system sections of the snakebite severity score (SSS) from Baseline (pre-dosing) to Day 7, among patients randomized ≤5 hours after bite.
- Patient Specific Functional Scale (PSFS) score at Day 3.
- Patient Specific Functional Scale (PSFS) score at Day 7.
- Complete snakebite severity score (SSS) recovery, defined as a composite score of 0 for the pulmonary, cardiovascular, local wound, hematologic, renal, and nervous system sections of the SSS at Day 28.
- Total antivenom requirement from Baseline through Day 28
- Duration of hospitalization from Baseline through Day 28

11.1.4.3 Exploratory Endpoints

The exploratory efficacy endpoints are to evaluate the:

- Change in the composite outcome of the 6-item SSS (pulmonary, cardiovascular, local wound, hematologic, renal, and nervous system subscores) from Baseline through Day 28
- Change in the 6-item SSS from Baseline through Day 28 using compressed SSS scale
- Individual SSS subscores from Baseline through Day 28
- Focused hematologic score based on INR and platelets from Baseline through Day 28
- Coagulation abnormalities (separately PT, PTT, platelets and fibrinogen) from Baseline through Day 28
- Change in NPRS from Baseline through Day 28
- Clinical Global Impression-Improvement (CGI-I) from Baseline through Day 28
- Patient Global Impression of Change (PGIC) from Baseline through Day 28
- Patient-Specific Functional Scale (PSFS) average score from Baseline through Day 28
- Levels of myonecrosis marker (CK) from Baseline through Day 3
- For patients bitten by elapids, change in 5-second head-lift duration from Baseline through Day 28
- For patients bitten by elapids, grip strength from Baseline through Day 28
- For patients bitten by elapids, duration of ventilatory support from Baseline through Day 28
- Analgesic use from Baseline through Day 28
- Elapsed time from initiation of IV varespladib treatment until the transition to oral varespladib methyl
- All cause mortality through Day 28
- Secretory phospholipase A_2 ($sPLA_2$) activity from Baseline through Day 7

11.1.4.4 Primary Analysis

The ITT and PP analysis populations will be used to analyze the primary efficacy endpoint. The ITT population will be the primary population for the efficacy analyses.

Descriptive statistics for the 5-second head-lift in elapids and the abbreviated SSS composed of Local Wound, Hematologic, and Nervous System subscores in vipers will be summarized by treatment group. The SAP will present a detailed algorithm for scoring the SSS.

For subjects bitten by elapids, the time for complete head-lift recovery defined by a 5-second head-lift of 5 seconds will be compared between treatment groups with a linear model with the p-value calculated using a randomization test at the 5% two-sided significance level. Because the time to head lift of 5 seconds is measured at discrete time intervals, when a person first lifts his or her head for 5 seconds, the assigned time will be assumed to be half-way between the previous time of assessment and the time of successful head lift.

For subjects bitten by vipers, the area Under the Curve (AUC) of an abbreviated Snakebite Severity Score (SSS) composed of local wound, hematologic, and neurologic subscores from Baseline (pre-dosing) to Day 14 will be compared between treatment groups with an analysis of covariance model (ANCOVA) with baseline abbreviated SSS, country, and snake type as covariates. The comparison of varespladib to placebo will be tested at the 5% two-sided significance level.

The independent two-sided p-values from the elapid and viper models will be analyzed using Fisher's combined probability test to obtain a single global p-value for testing the primary endpoint, which will be tested at the 5% two-sided significance level. An overall benefit will only be declared if the p-values for both the viper and elapid groups directionally favor treatment with varespladib.

11.1.4.5 Secondary Analyses

Analysis of the secondary endpoints will be carried out for the ITT population.

All secondary endpoints will be summarized using frequencies and percentages for categorical endpoints, and numeric summary statistics (e.g., means, standard deviations, medians, quartiles) for quantitative endpoints.

Additional ANCOVA or a mixed model for repeated measures (MMRM) analyses will be considered for quantitative secondary endpoints. Logistic regressions will be considered for binary outcomes.

Additional details of the efficacy analyses will be given in the final SAP.

11.1.4.6 Exploratory, Corroborative, Sensitivity, and Other Analyses

Exploratory endpoint efficacy data will be summarized descriptively by visit within treatment groups.

All $sPLA_2$ levels from beginning of IP dosing to discharge will be presented and used to calculate maximum concentrations in both treatment groups and compare concentration-time curves using a nonlinear regression to quantify a change in the half-maximal effective concentration (EC_{50}) and its confidence interval, and to compute a *p*-value that tests the null hypothesis that there is no difference between curves.

This study will not have an interim analysis; however, on or about September 1, 2024, Ophirex

will calculate the variance for the primary endpoint (assessed separately for elapids and for vipers) using all available data. Calculations will be based on blinded data only, and results will remain blinded. This information will be used to recalculate the power of the study and to support decisions regarding whether or not to close the study or to continue enrollment until the total sample size is enrolled. The Statistical Analysis Plan will provide details regarding the variance calculation.

11.1.5 Clinical Pharmacology Analyses

11.1.5.1 Pharmacokinetics

For noncompartmental analysis, plasma concentrations will be listed and summarized at each time point using descriptive statistics; graphical representations will also be provided. The PK parameters will be summarized by dose using descriptive statistics.

11.1.6 Safety and Tolerability Analyses

All safety analyses will be performed on the Safety Population.

The variables for safety endpoints are TEAEs, vital signs, laboratory evaluations, 12-lead ECGs, physical examinations, and concomitant medications and therapies.

11.1.6.1 Adverse Events

Treatment-emergent AEs are defined as AEs that first occur or worsen in severity after the first dose of IP and prior to the 30 days after last administration of IP. AEs will be coded using the Medical Dictionary for Regulator Activities (MedDRA). For each treatment group, the number of TEAEs and incidence rates will be tabulated by preferred term and System Organ Class.

Treatment-emergent AEs, SAEs, TEAEs leading to death, and TEAEs leading to discontinuation of IP will be summarized for each treatment group based on frequency, maximum severity, relationship to IP.

11.1.6.2 Clinical Laboratory Evaluations

All serum chemistry, hematology, urinalysis, liver function tests, coagulation profile measurements, biomarker levels, and $sPLA_2$ levels will be summarized for each treatment group using descriptive statistics by visit for observed values and changes from Baseline.

Shift tables for measurements described above, summarizing the shifts from Baseline (high; normal; low) at each post-Baseline visit, will be presented.

11.1.6.3 Vital Signs

All vital signs will be summarized for each treatment group using descriptive statistics at each visit for raw numbers and change from Baseline.

11.1.6.4 12-lead Electrocardiograms

All ECG measurements will be summarized for each treatment group using descriptive statistics by visit for raw numbers and change from Baseline.

Additionally, frequencies and percentages of Investigator ECG interpretations by visit will be presented.

11.1.6.5 Physical Examination Findings

The number and percentage of subjects with normal and abnormal findings in the complete physical examination will be displayed for each treatment group.

11.1.6.6 Biomarkers

Observed values and changes from Baseline in biomarkers such as CK will be summarized using descriptive statistics by visit.

11.2 Sample Size Determination

The planned approximate total enrollment is 140 subjects. The randomization scheme will be 1:1 with approximately one-half of subjects randomized to receive varespladib plus SOC and approximately one-half randomized to receive placebo plus SOC. Randomization will be stratified by snake type (copperhead, Mojave rattlesnake, non-Mojave rattlesnake, Russell's viper, Elapid, other), and by age group: pediatrics aged 5 to 17 years or adults aged 18 years and older, resulting in 12 strata in total.

The determination of the effectiveness of varespladib-methyl versus placebo will be based on the primary efficacy endpoint. The sample size for this phase 2 study was based on a formal power calculation that reflects data obtained from a similar study of oral varespladib for snakebite envenoming (OPX-PR-01).

The following assumptions were used to calculate power of the primary analysis:

- P-values for elapids and vipers are based on two-sided 5% Type 1 error rates.
- Randomization Ratio 1:1. Randomization will be stratified by snake type: copperhead, Mojave rattlesnake, non-Mojave rattlesnake, Russell's viper, Elapid, or other, and by age group: pediatrics aged 5 to 17 years or adults aged 18 years and older.
- The power for the elapid analysis will be greater than 90%. To determine power, we assumed 44 participants with elapid bites and calculated a mean difference in time to recovery between the treatment groups based on expected outcomes. The mean difference based on expected outcomes was then compared to the distribution of mean differences for 200 cases in which varespladib and placebo were randomly assigned in a 1:1 ratio (22 for each treatment group) to the expected outcome data.
- The power for showing a difference between varespladib and placebo in the viper analysis will be approximately 77%. This calculation assumes the study will enroll at least 120 viper-bite patients, a difference in the relevant outcome (composite local wound, hematology, and neurologic subscores of the SSS AUC from Baseline to Day 14) of 125 points and a standard deviation of 225.

- Further, it assumes that roughly 10% of randomized patients will fail to complete the study and that those patients will be assigned imputed values as if they had been assigned placebo.

The primary outcome will be tested using Fisher's combination of p-values at a two-sided 5% Type 1 error rate. Under the above conditions, the study will have over 90% power to show a statistically significant difference between varespladib and placebo.

12 STUDY CONDUCT

Steps to ensure the accuracy and reliability of data include the selection of qualified Investigators and appropriate study sites, review of protocol procedures with the Investigator and associated personnel before the study, periodic monitoring visits, and meticulous data management.

12.1 Sponsor and Investigator Responsibilities

12.1.1 Sponsor Responsibilities

The sponsor is obligated to conduct the study in accordance with strict ethical principles (Section 15). The sponsor reserves the right to withdraw a subject from the study (Section 7.3), to terminate participation of a study site at any time (Section 13.7), and/or to discontinue the study (Section 13.6).

Ophirex, Inc. agrees to provide the Investigator with sufficient material and support to permit the Investigator to conduct the study according to the study protocol.

12.1.2 Investigator Responsibilities

By signing the Investigator's Agreement (Section 17.1), the Investigator indicates that he or she has read the protocol carefully, fully understands the requirements, and agrees to conduct the study in accordance with the procedures and requirements described in this protocol.

The study will be conducted in accordance with ICH Good Clinical Practice (GCP) and applicable U.S. Code of Federal Regulations (CFR). The principal Investigator will assure that no deviation from, or changes to, the protocol will take place without prior agreement from the IND sponsor, funding agency (Department of Defense) and documented approval from the IRB, except where necessary to eliminate an immediate hazard(s) to the study subjects. All personnel involved in the conduct of this study have completed Human Subjects Protection and ICH GCP training.

Investigators should ensure that all persons who are delegated study-related responsibilities are adequately qualified and informed about the protocol, the IPs, and their specific duties within the context of the study. Investigators are responsible for providing Ophirex, Inc. with documentation of the qualifications, GCP training, and research experience for themselves and their staff as required by the sponsor and the relevant governing authorities.

To ensure compliance with the guidelines, the study may be audited by an independent person. The Investigator agrees, by written consent to this protocol, to cooperate fully with compliance checks by allowing access to all study documentation by authorized individuals.

12.1.3 Confidentiality and Privacy

Subject confidentiality and privacy must be strictly held in trust by the participating Investigators, their staff, and the sponsor(s) and their interventions. This confidentiality is extended to cover testing of biological samples and genetic tests in addition to the clinical information relating to subjects. Therefore, the study protocol, documentation, data, and all other information generated must be held in strict confidence. No information concerning the study, or the data will be released to any unauthorized third party without prior written approval of the sponsor.

All research activities will be conducted in as private a setting as possible.

The study monitor, other authorized representatives of the sponsor, representatives of the IRB, regulatory agencies, or pharmaceutical company supplying study product may inspect all documents and records required to be maintained by the Investigator, including, but not limited to, medical records (office, clinic, or hospital) and pharmacy records for the subjects in this study. This study is funded by the Department of Defense (DoD) and therefore representatives of the DoD are authorized to review research records. The clinical study site will permit access to such records.

The study subject's contact information will be securely stored at each clinical site for internal use during the study. At the end of the study, all records will continue to be kept in a secure location for as long a period as dictated by the reviewing IRB, institutional policies, or sponsor requirements.

Study subject research data, which is for purposes of statistical analysis and scientific reporting, will be transmitted to and stored at the study CRO. This will not include the subject's contact or identifying information. Rather, individual subjects and their research data will be identified by a unique study identification number. The study data entry and study management systems used by clinical sites and the study CRO will be secured and password protected. At the end of the study, all study databases will be de-identified and archived.

12.2 Site Initiation

Study personnel may not screen or enroll subjects into the study until after receiving notification from the sponsor or its designee that the study can be initiated at the study site. The study site will not be authorized for study initiation until:

1. The study site has received the appropriate IRB or Ethics Committee approval for the protocol and the appropriate informed consent form (ICF).
2. All regulatory documents have been submitted to and approved by the sponsor or its designee. (Department of Defense-supported research sites will require Office of Human Research Oversight (OHRO) review and approval prior to initiation.)

3. The study site has a Clinical Trial Agreement in place.
4. The study site Federal Wide Assurance has been verified
5. Study site personnel, including the Investigator, have participated in a study initiation meeting.

12.3 Screen Failures

Screen failures are defined as subjects who consent to participate in the clinical study but are not subsequently randomly assigned to the study intervention or entered in the study. A minimal set of screen failure information is required to ensure transparent reporting of screen failure subjects to meet the Consolidated Standards of Reporting Trials (CONSORT) publishing requirements and to respond to queries from regulatory authorities. Minimal information includes demography, screen failure details, eligibility criteria, and any SAE.

12.4 Study Documents

All documentation and material provided by Ophirex, Inc. for this study are to be retained in a secure location and treated as confidential material.

12.4.1 Informed Consent

Consent forms describing in detail the study intervention, study procedures, and risks will be given to the subject, and written documentation of informed consent is required prior to starting intervention/administering study intervention. As snakebites may constitute life-threatening medical conditions that necessitate urgent intervention, a legally authorized representative may provide consent for subjects who are not able to provide informed consent because of the severity of their condition.

The abbreviated consent is an optional document that may be used as the basis for randomization of patients but not for the basis of initiating the study drug. The abbreviated consent contains all the important elements of the full consent. The use of the abbreviated consent is anticipated to reduce the time from bite to initiation of study drug, which may have therapeutic value to patients randomized to active drug and will also allow the study to better approximate the time frame for initiating the study drug if given as part of usual clinical care to patients presenting to the emergency department with snakebite envenoming. No patient will receive any study drug until they or a legally authorized representative has signed the full consent.

Informed consent is a process that is initiated prior to the individual's agreeing to participate in the study and continues throughout the individual's study participation. Consent forms will be IRB-approved and the subject will be asked to read and review the document. The Investigator will explain the research study to the subject and answer any questions that may arise. A verbal explanation will be provided in terms suited to the subject's comprehension of the purposes, procedures, and potential risks of the study and of their rights as research subjects. Subjects will have the opportunity to carefully review the written consent form and ask questions prior to signing. The subjects should have the opportunity to discuss the study with their family or surrogates or think about it prior to agreeing to participate. The subject

will sign the informed consent document prior to any procedures being done specifically for the study. Subjects must be informed that participation is voluntary and that they may withdraw from the study at any time, without prejudice. A copy of the informed consent document and of the abbreviated consent form, if used, will be given to the subjects for their records. The informed consent process will be conducted and documented in the source document (including the date) and the form signed before the subject undergoes any study-specific procedures. The rights and welfare of the subjects will be protected by emphasizing to them that the quality of their medical care will not be adversely affected if they decline to participate in this study.

12.4.2 Investigator's Regulatory Documents

The regulatory documents are listed in the study plan.

The regulatory documents must be received from the Investigator and reviewed and approved by Ophirex, Inc. or its designee before the study site can initiate the study and before Ophirex, Inc. will authorize shipment of IP to the study site. Copies of the Investigator's regulatory documents must be retained at the study site in a secure location. Additional documents, including a copy of the protocol and applicable amendment(s), the varespladib IB, eCRF completion guidelines, copies of regulatory references, copies of IRB correspondence, and IP accountability records should also be retained as part of the Investigator's regulatory documents. It is the Investigator's responsibility to ensure that copies of all required regulatory documents are organized, current, and available for inspection.

12.4.3 Case Report Forms

By signing the Investigator's Agreement (Section 17.1), the Investigator agrees to maintain accurate eCRFs and source documentation as part of the case histories for all subjects who sign an ICF.

Electronic case report forms are considered confidential documents and should be protected accordingly. The sponsor or its designee will provide the necessary training on the use of the specific eCRF system used during the study to ensure that the study information is captured accurately and appropriately.

To ensure data accuracy, eCRF data for individual subject visits should be completed as soon as possible after the visit. All requested information must be entered in the electronic data capture (EDC) system according to the completion guidelines provided by the sponsor or its designee.

The eCRFs must be signed by the Investigator or a sub-Investigator. These signatures serve to attest that the information contained in the eCRF is accurate and true.

12.4.4 Source Documents

Information recorded in the EDC system should be supported by corresponding source documentation. Examples of acceptable source documentation include, but are not limited to,

hospital records, clinic and office charts, laboratory notes, and recorded data from automated instruments, memoranda, and pharmacy dispensing records. Source data permits not only reporting and analysis but also verification throughout the study for the purposes of confirmation, quality control, audit, or inspection. It is important that source data and the records that hold those data are:

- Accurate
- Legible
- Contemporaneous
- Original
- Attributable
- Complete
- Consistent
- Enduring
- Available when needed

Study monitors will perform ongoing source data verification to confirm that critical protocol data (i.e., source data) entered into the eCRFs by authorized site personnel are accurate, complete, and verifiable from source documents.

Before study initiation, the types of source documents that are to be generated will be clearly defined in the study plan. Any protocol data that will be permitted to be entered directly into the eCRFs (i.e., with no prior written or electronic record of the data) will be specified.

Source documents that are required to verify the validity and completeness of data entered into the eCRFs must not be obliterated or destroyed and must be retained per the policy for retention of records described below.

12.5 Data Quality Control

Ophirex, Inc. and its designees will perform quality control checks on this clinical study.

12.5.1 Monitoring Procedures

Ophirex, Inc. and/or its designee will conduct site visits to monitor the study and ensure compliance with the protocol, GCP, and applicable regulations and guidelines. The assigned clinical research associate(s) (CRA[s]) will visit the Investigator and study site at periodic intervals and maintain periodic communication. The Investigator agrees to allow the CRA(s) and other authorized Ophirex, Inc. personnel access. The CRA(s) will maintain current personal knowledge of the study through observation, review of study records and source documentation, and discussion of the conduct of the study with the Investigator and staff. While on site, the CRA(s) will review

- Regulatory documents, directly comparing entries in the EDC system with the source documents
- Consenting procedures
- AE procedures
- Storage and accountability of IP and study materials

The CRA will ask for clarification and/or correction of any noted inconsistencies. Procedures for correcting eCRF are described in the study plan. As representatives of the sponsor, CRAs are responsible for notifying project management of any noted protocol deviations.

By signing the Investigator's Agreement (Section 17.1), the Investigator agrees to meet with the CRA(s) during study site visits; to ensure that study staff is available to the CRA(s) as needed; to provide the CRA(s) access to all study documentation, to the clinical supplies dispensing and storage area; and to assist the monitors in their activities, if requested. Further, the Investigator agrees to allow Ophirex, Inc. or designee auditors or inspectors from regulatory agencies to review records and to assist the inspectors in their duties, if requested.

For additional information, please refer to the clinical monitoring plan.

12.5.2 Data Management

Ophirex, Inc. or designee will be responsible for activities associated with the data management of this study. The standard procedures for handling and processing records will be followed per GCP and the study CRO's standard operating procedures. A comprehensive data management plan will be developed, including a data management overview, description of database contents, annotated CRF, pre-entry review list, self-evident correction conventions, query contacts, and consistency checks.

Study site personnel will be responsible for providing resolutions to all data queries. The Investigator will be required to document electronic data review to ensure the accuracy of the corrected and/or clarified data. Procedures for soliciting and documenting resolution to data queries are described in the study plan.

12.5.3 Quality Assurance/Audit

This study will be subject to audit by Ophirex, Inc. or its designee. Audits may be performed to check compliance with GCP guidelines and can include:

- site audits
- Trial Master File (TMF) audits
- database audits
- document audits (e.g., protocol and/or CSR)

Ophirex, Inc. or its designee may conduct additional audits on a selection of study sites, requiring access to subject notes, study documentation, and facilities or laboratories used for the study.

The study site, facilities, all data (including source data), and documentation will be made available for audit by quality assurance auditors and for IRB or regulatory authorities according to GCP guidelines. The Investigator agrees to cooperate with the auditor during the visit and will be available to supply the auditor with CRFs or other files necessary to conduct that audit. Any findings will be strictly confidential.

If a regulatory authority informs the Investigator that it intends to conduct an inspection, the Investigator shall notify Ophirex, Inc. immediately. Ophirex, Inc. will then promptly notify the OHRO of a pending regulatory inspection.

12.6 Study Termination

The study may be terminated at Ophirex, Inc.'s discretion at any time and for any reason.

12.6.1 Premature Study Termination

The study may be temporarily suspended or terminated prematurely at any time, for any reason, by Ophirex, Inc.

Written notification documenting the reason for study suspension or termination will be provided by the sponsor to Investigators, IRBs, and appropriate regulatory authorities. If the study is prematurely terminated or suspended, the Investigator will promptly inform study subjects. Study subjects will be contacted, as applicable, and be informed of changes to study visit schedule.

Circumstances that may warrant termination or suspension include, but are not limited to:

- Determination of unexpected, significant, or unacceptable risk to subjects
- Insufficient compliance to protocol requirements
- Any instances of serious or continuing noncompliance with the federal regulations or IRB requirements
- Data that are not sufficiently complete and/or evaluable
- Sponsor may terminate the study at any time for any reason.

Study sites may be asked to have all subjects currently participating in the study complete all the assessments for the Early Termination Visit.

The study may resume once concerns about safety, protocol compliance, and data quality are addressed, and satisfy the sponsor, IRB and/or appropriate regulatory authority.

12.7 Study Site Closure

At the end of the study, all study sites will be closed. Ophirex, Inc. may terminate participation of a study site at any time. Examples of conditions that may require premature termination of a study site include, but are not limited to, the following:

- Noncompliance with the protocol and/or applicable regulations and guidelines
- Inadequate subject enrollment

12.7.1 Record Retention

The Investigator shall retain and preserve 1 copy of all data generated during the study, specifically including, but not limited to, those defined by GCP as essential until:

- At least 3 years after the last marketing authorization for the IP has been approved or the sponsor has discontinued its research with the IP, or
- At least 3 years have elapsed since the formal discontinuation of clinical development of the IP.

These documents should be retained for a longer period, however, if required by the applicable regulatory requirement(s) or if needed by the sponsor.

At the end of such period, the Investigator shall notify the sponsor in writing of his or her intent to destroy all such material. The sponsor has 30 days to respond to the Investigator’s notice, and the sponsor has further opportunity to retain such materials at the sponsor’s expense.

12.8 Changes to the Protocol

This protocol cannot be altered or changed except through a formal protocol amendment by Ophirex, Inc. The protocol amendment must be signed by the Investigator and approved by the IRB and OHRO before it may be implemented. Protocol amendments will be filed with the appropriate regulatory agency(s) having jurisdiction over the conduct of the study.

12.9 Use of Information and Publication

All information concerning varespladib, varespladib-methyl, Ophirex, Inc.’s operations, patent applications, formula, manufacturing processes, basic scientific data, and formulation information supplied by Ophirex, Inc. or its designee to the Investigator, and not previously published, is considered confidential and remains the sole property of Ophirex, Inc. Case report forms also remain the property of Ophirex, Inc. The Investigator agrees to use this information for purposes of study execution through finalization.

The information developed in this study will be used by Ophirex, Inc. in connection with the continued development of varespladib or varespladib-methyl and thus may be disclosed as required to other clinical Investigators or government regulatory agencies.

The information generated by this study is the property of Ophirex, Inc. Publication or other public presentation of varespladib or varespladib-methyl data resulting from this study requires prior review and written approval of Ophirex, Inc. Abstracts, manuscripts, and presentation materials should be provided to Ophirex, Inc. for review at least 30 days prior to the relevant submission deadline. It is agreed that the results of the study will not be submitted for presentation, abstract, poster exhibition, or publication by the Investigator until Ophirex, Inc. has reviewed and commented on such a presentation or manuscript for publication. If applicable, this study will be registered at ClinicalTrials.gov, and results from this study will be submitted to ClinicalTrials.gov.

13 FINAL CLINICAL STUDY REPORT

Ophirex, Inc. will retain ownership of the data.

The final CSR will be written within 6 months of completion of the clinical part of the study. This report will include a summary of the study results based on a statistical evaluation and clinical assessment of the protocol-defined endpoints.

The final study results will be submitted to the regulatory authorities and OHRO.

14 ETHICAL AND LEGAL CONSIDERATIONS

14.1 Good Clinical Practice

This study will be conducted in compliance with the April 1996 ICH Guidance for Industry GCP E6 (including archiving of essential study documents), the Integrated Addendum to ICH E6 (R2) of November 2016, and the applicable regulations of the country in which the study is conducted.

14.2 Subject Information and Informed Consent and/or Assent

A properly constituted, valid IRB or EC must review and approve the protocol, the Investigator's ICF, and related subject information and recruitment materials before the start of the study.

It is the responsibility of the Investigator to ensure that written informed consent and/or assent is obtained from the subject before any activity or procedure is undertaken that is not part of routine care.

If any enrolled participant becomes a prisoner and thus a vulnerable subject, the investigator must promptly report this information to their IRB and to the sponsor, who will report the information to the OHRO of the U.S. Army Medical Research and Development Command.

14.3 Approval by Institutional Review Board or Ethics Committee

For IND studies, the minimum standards of conduct and requirements for informed consent and/or assent are defined in the FDA regulations.

A valid IRB or EC must review and approve this protocol before study initiation. Written notification of approval is to be provided by the Investigator to the sponsor's designated contact before shipment of IP supplies, and will include the date of the committee's approval and the chairperson's signature.

Until written approval by the IRB or EC has been received by the Investigator, no subject may undergo any procedure not part of routine care for the subject's condition.

Protocol amendments must also be reviewed and approved by the IRB or EC. Written approval from the IRB or EC or a designee must be received by Ophirex, Inc. before implementation.

Prior to implementation of any actions, OHRO approval is required. In addition, any amendments will also require OHRO approval prior to implementation.

14.4 Finance and Insurance

The Department of Defense, United States Army Medical Materiel Development Activity (USAMMDA), is funding this study. Details on finance and insurance will be provided in a separate agreement between the Investigator and the sponsor.

15 REFERENCES

Adis. Varespladib. American Journal of Cardiovascular Drugs. 2011;11:137-143.

Alangode A, Rajan K, Nair BG. Snake antivenom: Challenges and alternate approaches. Biochem Pharmacol. 2020a;181:114135.

Alangode A, Reick M, Reick M. Sodium oleate, arachidonate, and linoleate enhance fibrinogenolysis by Russell's viper venom proteinases and inhibit FXIIIa; a role for phospholipase A2 in venom induced consumption coagulopathy. Toxicon. 2020b;186:83-93.

Albulescu LO, Xie C, Ainsworth S, et al. A therapeutic combination of two small molecule toxin inhibitors provides broad preclinical efficacy against viper snakebite. Nat Commun. 2020;11(1):6094.

Anderson BO, Moore EE, Banerjee A. Phospholipase A2 regulates critical inflammatory mediators of multiple organ failure. J Surg Res. 1994;56(2):199-205.

Anil A, Singh S, Bhalla A, Sharma N, Agarwal R, Simpson ID. Role of neostigmine and polyvalent antivenom in Indian common krait (*Bungarus caeruleus*) bite. J Infect Public Health. 2010;3(2):83-87.

Arce-Bejarano R, Lomonte B, Gutierrez JM. Intravascular hemolysis induced by the venom of the Eastern coral snake, *Micrurus fulvius*, in a mouse model: identification of directly hemolytic phospholipases A2. Toxicon. 2014;90:26-35.

Barbosa ED, Lima Neto JX, Bezerra KS, Oliveira JIN, Machado LD, Fulco UL. Quantum Biochemical Investigation of Lys49-PLA2 from *Bothrops moojeni*. J Phys Chem B. 2021;125(47):12972-12980.

Bickler PE. Amplification of Snake Venom Toxicity by Endogenous Signaling Pathways. Toxins (Basel). 2020;12(2).

Bittenbinder MA, Zdenek CN, Op den Brouw B, et al. Coagulotoxic Cobras: Clinical Implications of Strong Anticoagulant Actions of African Spitting *Naja* Venoms That Are Not Neutralised by Antivenom but Are by LY315920 (Varespladib). Toxins (Basel). 2018;10(12).

Bocian A, Urbanik M, Hus K, et al. Proteome and Peptidome of *Vipera berus* Venom. Molecules. 2016;21(10).

Bryan-Quiros W, Fernandez J, Gutierrez JM, Lewin MR, Lomonte B. Neutralizing properties of LY315920 toward snake venom group I and II myotoxic phospholipases A2. Toxicon. 2019;157:1-7.

Bulfone TC, Samuel SP, Bickler PE, Lewin MR. Developing Small Molecule Therapeutics for the Initial and Adjunctive Treatment of Snakebite. J Trop Med. 2018;2018:4320175.

Busner J, Targum SD. The clinical global impressions scale: applying a research tool in clinical practice. Psychiatry. 2007 Jul;4(7):28-37.

Dart RC, Hurlbut KM, Garcia R, Boren J. Validation of a severity score for the assessment of crotalid snakebite. Ann Emerg Med. 1996;27(3):321-326.

Fernandez ML, Quartino PY, Arce-Bejarano R, et al. Intravascular hemolysis induced by phospholipases A2 from the venom of the Eastern coral snake, *Micrurus fulvius*: Functional profiles of hemolytic and non-hemolytic isoforms. Toxicol Lett. 2018;286:39-47.

Ferraz CR, Arrahman A, Xie C, et al. Multifunctional Toxins in Snake Venoms and Therapeutic Implications: From Pain to Hemorrhage and Necrosis. Frontiers in Ecology and Evolution. 2019;7(218).

Food and Drug Administration (FDA), 2020. Drug Development and Drug Interactions | Table of Substrates, Inhibitors and Inducers. Accessed 08 June 2021. https://www.fda.gov/drugs/drug-interactions-labeling/drug-development-and-drug-interactions-table-substrates-inhibitors-and-inducers

Gerardo CJ, Vissoci JRN, Evans CS, Simel DL, Lavonas EJ. Does This Patient Have a Severe Snake Envenomation?: The Rational Clinical Examination Systematic Review. JAMA Surg. 2019;154(4):346-354.

Gopalakrishnan N. Snake Envenoming—An Underreported Cause of Acute Kidney Injury. Kidney Int Rep. 2019;4(5):643-646.

Gutierrez JM, Calvete JJ, Habib AG, Harrison RA, Williams DJ, Warrell DA. Snakebite envenoming. Nat Rev Dis Primers. 2017;3:17063.

Gutierrez JM, Lomonte B. Phospholipases A2: unveiling the secrets of a functionally versatile group of snake venom toxins. Toxicon. 2013;62:27-39.

Kerns RT, Kini RM, Stefansson S, Evans HJ. Targeting of venom phospholipases: the strongly anticoagulant phospholipase A(2) from *Naja nigricollis* venom binds to coagulation factor Xa to inhibit the prothrombinase complex. Arch Biochem Biophys. 1999;369(1):107-113.

Kini RM. Excitement ahead: structure, function and mechanism of snake venom phospholipase A2 enzymes. Toxicon. 2003;42(8):827-840.

Kini RM. Anticoagulant proteins from snake venoms: structure, function and mechanism. Biochem J. 2006;397(3):377-387.

Lewin MR, Samuel SP, Merkel J, Bickler P. Varespladib (LY315920) Appears to Be a Potent, Broad-Spectrum, Inhibitor of Snake Venom Phospholipase A2 and a Possible Pre-Referral Treatment for Envenomation. Toxins (Basel). 2016;8(9).

Lewin MR, Gutierrez JM, Samuel SP, et al. Delayed Oral LY333013 Rescues Mice from Highly Neurotoxic, Lethal Doses of Papuan Taipan (*Oxyuranus scutellatus*) Venom. Toxins (Basel). 2018a;10(10).

Lewin MR, Gilliam LL, Gilliam J, et al. Delayed LY333013 (Oral) and LY315920 (Intravenous) Reverse Severe Neurotoxicity and Rescue Juvenile Pigs from Lethal Doses of *Micrurus fulvius* (Eastern Coral Snake) Venom. Toxins (Basel). 2018b;10(11).

Lomonte B, Rangel J. Snake venom Lys49 myotoxins: From phospholipases A(2) to non-enzymatic membrane disruptors. Toxicon. 2012;60(4):520-530.

Lomonte B, Tsai WC, Urena-Diaz JM, et al. Venomics of New World pit vipers: genus-wide comparisons of venom proteomes across *Agkistrodon*. J Proteomics. 2014;96:103-116.

Longbottom J, Shearer FM, Devine M, et al. Vulnerability to snakebite envenoming: a global mapping of hotspots. Lancet. 2018;392(10148):673-684.

Otero R, Gutierrez J, Beatriz Mesa M, et al. Complications of *Bothrops, Porthidium*, and *Bothriechis* snakebites in Colombia. A clinical and epidemiological study of 39 cases attended in a university hospital. Toxicon. 2002;40(8):1107-1114.

Park EJ, Choi S, Kim HH, Jung YS. Novel Treatment Strategy for Patients with Venom-Induced Consumptive Coagulopathy from a Pit Viper Bite. Toxins (Basel). 2020;12(5).

Pla D, Sanz L, Quesada-Bernat S, et al. Phylovenomics of *Daboia russelii* across the Indian subcontinent. Bioactivities and comparative *in vivo* neutralization and *in vitro* third-generation antivenomics of antivenoms against venoms from India, Bangladesh and Sri Lanka. J Proteomics. 2019;207:103443.

Prasarnpun S, Walsh J, Awad SS, Harris JB. Envenoming bites by kraits: the biological basis of treatment-resistant neuromuscular paralysis. Brain. 2005;128(Pt 12):2987-2996.

Ranawaka UK, Lalloo DG, de Silva HJ. Neurotoxicity in snakebite—the limits of our knowledge. PLoS Negl Trop Dis. 2013;7(10):e2302.

Saikia D, Thakur R, Mukherjee AK. An acidic phospholipase A(2) (RVVA-PLA(2)-I) purified from *Daboia russelli* venom exerts its anticoagulant activity by enzymatic hydrolysis of plasma phospholipids and by non-enzymatic inhibition of factor Xa in a phospholipids/Ca(2+) independent manner. Toxicon. 2011;57(6):841-850.

Salvador GHM, Borges RJ, Lomonte B, Lewin MR, Fontes MRM. The synthetic varespladib molecule is a multi-functional inhibitor for PLA2 and PLA2-like ophidic toxins. Biochim Biophys Acta Gen Subj. 2021;1865(7):129913.

Salvador GHM, Gomes AAS, Bryan-Quiros W, et al. Structural basis for phospholipase A2-like toxin inhibition by the synthetic compound Varespladib (LY315920). Sci Rep. 2019;9(1):17203.

Sehgal IS, Gandra RR, Dhooria S, et al. A randomised trial of adaptive support ventilation in patients with neuroparalytic snake envenomation. Br J Anaesth. 2022.

Senji Laxme RR, Khochare S, de Souza HF, et al. Beyond the 'big four': Venom profiling of the medically important yet neglected Indian snakes reveals disturbing antivenom deficiencies. PLoS Negl Trop Dis. 2019;13(12):e0007899.

Silva-Carvalho R, Gaspar MZ, Quadros LHB, et al. *In vivo* treatment with varespladib, a phospholipase A2 inhibitor, prevents the peripheral neurotoxicity and systemic disorders induced by *Micrurus corallinus* (coral snake) venom in rats. Toxicol Lett. 2021.

Snyder DW, Bach NJ, Dillard RD, et al. Pharmacology of LY315920/S-5920, [[3-(aminooxoacetyl)-2-ethyl-1- (phenylmethyl)-1H-indol-4-yl]oxy] acetate, a potent and selective secretory phospholipase A2 inhibitor: A new class of anti-inflammatory drugs, SPI. J Pharmacol Exp Ther. 1999;288(3):1117-1124.

Surm JM, Moran Y. Insights into how development and life-history dynamics shape the evolution of venom. Evodevo. 2021;12(1):1.

Tan CH, Lingam TMC, Tan KY. Varespladib (LY315920) rescued mice from fatal neurotoxicity caused by venoms of five major Asiatic kraits (*Bungarus spp.*) in an experimental envenoming and rescue model. Acta Tropica, in press. 2021.

Tanaka GD, Furtado Mde F, Portaro FC, Sant'Anna OA, Tambourgi DV. Diversity of *Micrurus* snake species related to their venom toxic effects and the prospective of antivenom neutralization. PLoS Negl Trop Dis. 2010;4(3):e622.

Tasoulis T, Isbister GK. A Review and Database of Snake Venom Proteomes. Toxins (Basel). 2017;9(9).

Vaiyapuri S, Vaiyapuri R, Ashokan R, et al. Snakebite and its socio-economic impact on the rural population of Tamil Nadu, India. PLoS One. 2013;8(11):e80090.

Williams DJ, Faiz MA, Abela-Ridder B, et al. Strategy for a globally coordinated response to a priority neglected tropical disease: Snakebite envenoming. PLoS Negl Trop Dis. 2019;13(2):e0007059.

Xiao H, Pan H, Liao K, Yang M, Huang C. Snake Venom PLA2, a Promising Target for Broad-Spectrum Antivenom Drug Development. Biomed Res Int. 2017;2017:6592820.

Xie C, Albulescu LO, Bittenbinder MA, et al. Neutralizing Effects of Small Molecule Inhibitors and Metal Chelators on Coagulopathic *Viperinae* Snake Venom Toxins. Biomedicines. 2020a;8(9).

Xie C, Albulescu LO, Still KBM, et al. Varespladib Inhibits the Phospholipase A2 and Coagulopathic Activities of Venom Components from Hemotoxic Snakes. Biomedicines. 2020b;8(6).

Xie J, Covassin N, Chahal AA, et al. Effect of Adaptive Servo-Ventilation on Periodic Limb Movements in Sleep in Patients With Heart Failure. Am J Cardiol. 2019;123(4):632-637.

Youngman NJ, Walker A, Naude A, Coster K, Sundman E, Fry BG. Varespladib (LY315920) neutralises phospholipase A2 mediated prothrombinase-inhibition induced by *Bitis* snake venoms. Comp Biochem Physiol C Toxicol Pharmacol. 2020;236:108818.

Zambelli VO, Picolo G, Fernandes CAH, Fontes MRM, Cury Y. Secreted Phospholipases A(2) from Animal Venoms in Pain and Analgesia. Toxins (Basel). 2017;9(12).

Ziganshin RH, Kovalchuk SI, Arapidi GP, et al. Quantitative proteomic analysis of Vietnamese krait venoms: Neurotoxins are the major components in *Bungarus multicinctus* and phospholipases A2 in *Bungarus fasciatus*. Toxicon. 2015;107(Pt B):197-209.

16 ATTACHMENTS

16.1 Investigator's Agreement

PROTOCOL NUMBER:	OPX-PR-03
PROTOCOL TITLE:	Randomized, Double-Blinded, Placebo-Controlled Study to Evaluate the Safety, Tolerability, and Efficacy of Intravenous Varespladib Followed by Oral Varespladib in Addition to Standard of Care in Subjects Bitten by Venomous Snakes
FINAL PROTOCOL:	Version 6.0, 21 October 2024

I have read this protocol and agree to conduct this clinical study as outlined herein. I will ensure that all sub-Investigators and other study staff members have read and understand all aspects of this protocol. I agree to cooperate fully with Ophirex, Inc. during the study. I will adhere to all FDA, ICH, and other applicable regulations and guidelines regarding clinical studies on an IP during and after study completion.

Principal Investigator:

Printed Name:

____

Signature:

____

Date:

____

APPENDICES

A. Scoring Sheets for Snakebite Severity Score Inclusion Criteria

B. Scoring Sheet for Composite Snakebite Severity Score Efficacy Endpoints

C. Study-specific Requirements

- C1a. Clinical Global Impression – Improvement (CGI-I)
- C1b. Clinical Global Impression - Expected Recovery (CGI-ER)
- C2. Clinical Global Impression – Severity of Illness (CGI-S)
- C3. Patient Global Impression of Change (PGIC)
- C4. Patient-Specific Functional Scale (PSFS)
- C5. Numeric Pain Rating Scale (NPRS)
- C6. Columbia-Suicide Severity Rating Scale
- C7. Neurological Evaluation (Grip Strength)
- C8. Modes of Mechanical Ventilation
- C9. Head-Lift Duration
- C10. Analgesic Use

D. COVID-19 Protocol Deviations

E. Regulations and GCP Guidelines

APPENDIX A SCORING SHEET FOR SNAKEBITE SEVERITY INCLUSION CRITERIA*

This Appendix provides information on scoring for inclusion of patients bitten by vipers who are screened within 5 hours of snakebite or symptom onset. Regardless of previous antivenom administration, subjects should be included if they present at the time of eligibility assessment with 2 points in any of the system scores shown below and 1 or more additional points in any other category below OR a 3 or greater in any category below. This inclusion criteria scoring should not wait until hematologic laboratories are evaluated. Point of care testing (e.g., 20WBCT) may be used for enrollment determinations for the Hematologic system, if performed per site standard of care. Severe abnormalities of other coagulation parameter laboratory values, including 20WBCT, should be scored as a 4 in the Hematologic system subscore for purposes of enrollment only. Appendix B should be used for Baseline and subsequent SSS scoring.

**Local wound**	
No signs/symptoms	0
Pain, swelling, or ecchymosis within 5–7.5 cm of bite site	1
Pain, swelling, or ecchymosis involving less than half the extremity (7.5–50 cm from bite site)	2
Pain, swelling, or ecchymosis involving half to all of extremity (50–100 cm from bite site)	3
Pain, swelling, or ecchymosis extending beyond affected extremity (more than 100 cm of bite site)	4
**Pulmonary symptoms**	
No signs/symptoms	0
Dyspnea, minimal chest tightness, mild/vague discomfort, respirations of 20–25 breaths per minute	1
Moderate respiratory distress, 26–40 bpm	2
Cyanosis, air hunger, extreme tachypnea, or respiratory insufficiency/failure	3
**Cardiovascular system**	
No signs/symptoms	0
HR 100–125 BPM, palpitations, generalized weakness, benign dysrhythmia, or hypertension	1
HR 126–175 BPM, or hypotension with SBP > 100 mmHg	2
HR > 175 BPM, or hypotension with SBP < 100 mmHg, malignant dysrhythmia, or cardiac arrest	3
**Hematologic symptoms**	
No signs/symptoms	0
Coagulation parameters slightly abnormal: PT ULN-20 secs, PTT ULN-50 secs, platelets 100–150K/mL, or fibrinogen 100–150 mcg/mL. (ULN defined by local lab.)	1
Coagulation parameters abnormal: PT 20–50 secs, PTT 50–75 secs, platelets 50–100K/mL, or fibrinogen 50–100 mcg/mL	2
Coagulation parameters abnormal: PT 50–100 secs, PTT 75–100secs, platelets 20–50K/mL, or fibrinogen < 50 mcg/mL	3
Coagulation parameters markedly abnormal, with serious bleeding or the threat of spontaneous bleeding; unmeasurable PT or PTT, platelets < 20 K/mL, undetectable fibrinogen, severe abnormalities of other laboratory values also fall into this category	4
**Nervous system**	
No signs/symptoms	0
Minimal weakness (patient reported weakness or ptosis, but normal 5-second head-lift), apprehension, headache, dizziness, chills, or paresthesia	1
Moderate weakness (5-second head-lift >0 and <5 seconds, difficulty swallowing, dysarthria, or dysconjugate gaze), apprehension, headache, dizziness, chills, paresthesia, confusion, fasciculation in area of bite site	2
Severe weakness (5-second head-lift = 0 second, flaccid paralysis), confusion, lethargy, seizures, coma, psychosis, or generalized fasciculation	3
TOTAL	

*Appendix A applies to SSS scoring for Inclusion Criteria Only. Appendix B describes baseline and additional SSS scoring used for Efficacy Endpoints.

APPENDIX B COMPOSITE SNAKEBITE SEVERITY SCORE FOR BASELINE AND OTHER EFFICACY ENDPOINTS

**Local wound**	
No signs/symptoms	0
Pain, swelling, or ecchymosis within 5–7.5 cm of bite site	1
Pain, swelling, or ecchymosis involving less than half the extremity (7.5–50 cm from bite site) Pain,	2
swelling, or ecchymosis involving half to all of extremity (50–100 cm from bite site)	3
Pain, swelling, or ecchymosis extending beyond affected extremity (more than 100 cm of bite site)	4
**Pulmonary symptoms**	
No signs/symptoms	0
Dyspnea, minimal chest tightness, mild/vague discomfort, respirations of 20–25 breaths per minute	1
Moderate respiratory distress, 26–40 bpm	2
Cyanosis, air hunger, extreme tachypnea, or respiratory insufficiency/failure	3
**Cardiovascular system**	
No signs/symptoms	0
HR 100–125 BPM, palpitations, generalized weakness, benign dysrhythmia, or hypertension HR 126–	1
175 BPM, or hypotension with SBP > 100 mmHg	2
HR > 175 BPM, or hypotension with SBP < 100 mmHg, malignant dysrhythmia, or cardiac arrest	3
**Gastrointestinal system**	
No signs/symptoms	0
Pain, tenesmus, or nausea	1
Vomiting or diarrhea	2
Repeated vomiting, diarrhea, hematemesis, or hematochezia	3
**Hematologic symptoms**	
No signs/symptoms	0
Coagulation parameters slightly abnormal: PT ULN-20 secs, PTT ULN-50 secs, platelets 100–150K/mL, or fibrinogen 100–150 mcg/mL. (ULN defined by local lab)	1
Coagulation parameters abnormal: PT 20–50 secs, PTT 50–75 secs, platelets 50–100K/mL, or fibrinogen 50–100 mcg/mL Coagulation	2
parameters abnormal: PT 50–100 secs, PTT 75–100secs, platelets 20–50K/mL, or fibrinogen < 50 mcg/mL	3
Coagulation parameters markedly abnormal, with serious bleeding or the threat of spontaneous bleeding; unmeasurable PT or PTT, platelets < 20 K/mL, undetectable fibrinogen, severe abnormalities of other laboratory values also fall into this category	4
**Nervous system**	
No signs/symptoms	0
Minimal apprehension, headache, weakness (patient-reported weakness or ptosis, but normal 5-second head-lift), dizziness, chills, or paresthesia	1
Moderate apprehension, headache, weakness (5-second head-lift >0 and <5 seconds, difficulty swallowing, dysarthria, or dysconjugate gaze), dizziness,	2
chills, paresthesia, confusion, or fasciculation in area of bite site	3
Severe confusion, lethargy, weakness (head-lift = 0 seconds or flaccid paralysis), seizures, coma, psychosis, or generalized fasciculation	
**Renal system**	
Normal creatinine and urine output	0
Creatinine 1.5 to 1.9 times baseline, increase in creatinine ≥0.3mg/dL (≥26.5 µmol/L) from baseline, or urine output <0.5 mL/kg/h for >6 h Creatinine 2	1
to 2.9 times baseline or urine output <0.5 mL/kg/h for >12 h	2
Creatinine ≥3.0 times baseline, increase in creatinine to ≥4.0 mg/dL (≥353.6 µmol/L), urine output <0.3 mL/kg/h for ≥24 h or anuria ≥12 h, or initiation of renal replacement therapy	3
TOTAL	

*Where applicable, confirmatory laboratory and physiologic test results will be used to score the SSS for both Baseline and efficacy endpoints.

APPENDIX C STUDY-SPECIFIC REQUIREMENTS

**Protocol Appendix**	**Assessment Tool**
C1a	Clinical Global Impression – Improvement (CGI-I)
C1b	Clinical Global Impression – Expected Recovery (CGI-ER)
C2	Clinical Global Impression – Severity of Illness (CGI-S) Scale
C3	Patient Global Impression of Change (PGIC)
C4	Patient-Specific Functional Scale (PSFS)
C5	Numeric Pain Rating Scale (NPRS)
C6	Columbia-Suicide Severity Rating Scales
C7	Neurological evaluation (Grip Strength)
C8	Modes of Mechanical Ventilation
C9	Head-Lift Assessment
C10	Analgesic Use

Appendix C1a: Clinical Global Impression-Improvement (CGI-I) Form

Baseline CGI-I rating should be in comparison to previous clinical assessment. All post-baseline CGI-I assessments should be compared to baseline CGI-I.

Patient Name: Date:

Clinician Name:

Clinical Global Impression – Global Improvement (CGI –I) Scale

Rate total improvement whether or not, in your clinical judgment, it is due entirely to drug treatment.

Compared to his/her condition at baseline, how much has he/she changed?

- ☐ 0 = Not assessed
- ☐ 1 = Very much improved
- ☐ 2 = Much improved
- ☐ 3 = Minimally improved
- ☐ 4 = No change
- ☐ 5 = Minimally worse
- ☐ 6 = Much worse
- ☐ 7 = Very much worse

Appendix C1b: Clinical Global Impression-Expected Recovery (CGI-ER)

Based on all aspects of the patient's initial presentation (including snake type, severity of envenoming, patient characteristics), please characterize the patient's recovery from the snakebite over the first week.

- ☐ Worse than expected
- ☐ As expected
- ☐ Better than expected

If worse or better than expected, please explain why you are characterizing the recovery in this way.

Appendix C2: Clinical Global Impression – Severity of Illness (CGI-S) Scale

Clinical Global Impression – Severity of Illness (CGI –S) Scale

Rate severity of illness, in your clinical judgment, based on typical venomous snakebite presentation.

Considering your total clinical experience with this particular population, how ill is the patient at this time?

- ☐ 0 = Not assessed
- ☐ 1 = Normal, not at all ill
- ☐ 2 = Borderline ill
- ☐ 3 = Mildly ill
- ☐ 4 = Moderately ill
- ☐ 5 = Markedly ill
- ☐ 6 = Severely ill
- ☐ 7 = Among the most extremely ill patients

Appendix C3: Patient Global Impression of Change Scale

Patients' Global Impression of Change (PGIC) scale.

Name:______________________________ **Date:**__________ **DOB:**__________

Chief Complaint:____________________________

Since beginning treatment at this clinic, how would you describe the change (if any) in ACTIVITY LIMITATIONS, SYMPTOMS, EMOTIONS and OVERALL QUALITY OF LIFE, related to your painful condition? (tick ONE box).

No change (or condition has gotten worse)	☐	1
Almost the same, hardly any change at all	☐	2
A little better, but no noticeable change	☐	3
Somewhat better, but the change has not made any real difference	☐	4
Moderately better, and a slight but noticeable change	☐	5
Better, and a definite improvement that has made a real and worthwhile difference	☐	6
A great deal better, and a considerable improvement that has made all the difference	☐	7

In a similar way, please circle the number below, that matches your degree of change since beginning care at this clinic:

Much Better					No Change					Much Worse
0	1	2	3	4	5	6	7	8	9	10

Protocol OPX-PR-01<br>Subject ID: ___ ___ ___-___ ___ ___<br>Date of Visit: ____/_____/______<br>DD / MMM / YYYY	Subject Signature________________________<br>Date________________________

Reference: Hurst H, Bolton J. Assessing the clinical significance of change scores recorded on subjective outcome measures. J Manipulative Physiol Ther 2004; 27:26-35.

Appendix C4: Patient-Specific Functional Scale

The PSFS is a 3-item instrument that is administered verbally. Completion takes approximately 2 minutes.

On the initial assessment, the question is: "I am going to ask you to identify 3 important activities that you are unable to do or are having difficulty with as a result of your snake bite. Today, are there any activities that you are unable to do or having difficulty with because of your snake bite?"

The patient then provides 3 activities. The patient then provides a rating for each item on an 11-item ordinal scale, where 0 is "unable to perform activity" and 10 is "able to perform activity at the same level as before injury or problem." The range of possible single activity scores is 0 to 10 and the range of possible total score is 0 to 10.

For all follow-up assessments, the question is: "When I assessed you on [state previous assessment date], you told me that you had difficulty with [read all activities from the list]. Today, do you still have difficulty with [read and have patient score each item on the list]?"

References:

Westaway MD, Stratford PW, Binkley JM. The patient-specific functional scale: validation of its use in persons with neck dysfunction. *J Orthop Sports Phys Ther* 1998; 27(5): 331-338.

Stratford P, Gill C, Westaway M, Binkley J. Assessing disability and change on individual patients: a report of a patient specific measure. *Physiotherap Canada* 1995; 47(4): 258-63.

Horn KK, Jennings S. Richardson G, van Vliet D, Hefford C, Abbott JH. The Patient-Specific Functional Scale: psychometrics, clinimetrics, and applications as a clinical outcome measure. J Orthop Sports *Phys Ther* 2012; 42(1):30-42.

Instrument: None

A sample PSFS assessment form is attached below.

The Patient-Specific Functional Scale

This useful questionnaire can be used to quantify activity limitation and measure functional outcome for patients with any orthopaedic condition.

Clinician to read and fill in below: Complete at the end of the history and prior to physical examination.

Initial Assessment:

I am going to ask you to identify up to three important activities that you are unable to do or are having difficulty with as a result of your ________________ problem. Today, are there any activities that you are unable to do or having difficulty with because of your ________________ problem? (Clinician: show scale to patient and have the patient rate each activity).

Follow-up Assessments:

When I assessed you on (state previous assessment date), you told me that you had difficulty with (read all activities from list at a time). Today, do you still have difficulty with: (read and have patient score each item in the list)?

Patient-specific activity scoring scheme (Point to one number):

0	1	2	3	4	5	6	7	8	9	10

Unable to perform activity

Able to perform activity at the same level as before injury or problem

(Date and Score)

Activity	Initial					
1.						
2.						
3.						

Total score = sum of the activity scores/number of activities
Minimum detectable change (90%CI) for average score = 2 points
Minimum detectable change (90%CI) for single activity score = 3 points

(C) 1995 P Stratford, reprinted with permission

Appendix C5: NPRS Form

The numeric pain rating scale (NPRS) is an 11-point scale for patient self-reporting of pain. This scale is administered verbally by the clinical site staff and recorded by the administrator. An example NPRS is provided below. For patients aged 5 to 7, locally available pain-rating scales used in children (e.g., the Wong-Baker FACES pain scale) may be used to support scoring.

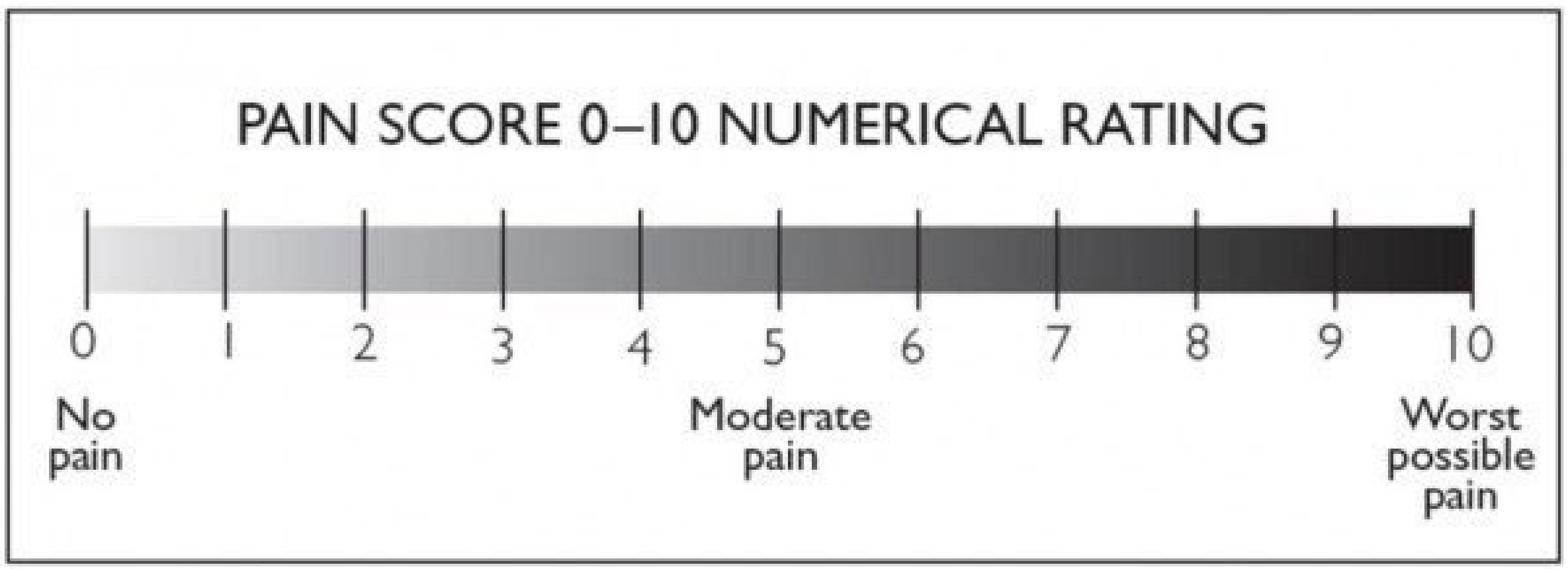

Appendix C6: Columbia-Suicide Severity Rating Scale Forms

1. C-SSRS Baseline/Screening Form
2. C-SSRS Since Last Visit Form

C-SSRS Baseline/Screening Form

COLUMBIA-SUICIDE SEVERITY RATING SCALE (C-SSRS)

Baseline/Screening Version

Version 1/14/09

Posner, K.; Brent, D.; Lucas, C.; Gould, M.; Stanley, B.; Brown, G.; Fisher, P.; Zelazny, J.; Burke, A.; Oquendo, M.; Mann, J.

Disclaimer:

This scale is intended to be used by individuals who have received training in its administration. The questions contained in the Columbia-Suicide Severity Rating Scale are suggested probes. Ultimately, the determination of the presence of suicidal ideation or behavior depends on the judgment of the individual administering the scale.

Definitions of behavioral suicidal events in this scale are based on those used in **The Columbia Suicide History Form***, developed by John Mann, MD and Maria Oquendo, MD, Conte Center for the Neuroscience of Mental Disorders (CCNMD), New York State Psychiatric Institute, 1051 Riverside Drive, New York, NY, 10032. (Oquendo M. A., Halberstam B. & Mann J. J., Risk factors for suicidal behavior: utility and limitations of research instruments. In M.B. First [Ed.]* Standardized Evaluation in Clinical Practice, *pp. 103 -130, 2003.)*

For reprints of the C-SSRS contact Kelly Posner, Ph.D., New York State Psychiatric Institute, 1051 Riverside Drive, New York, New York, 10032; inquiries and training requirements contact posnerk@nyspi.columbia.edu

© 2008 The Research Foundation for Mental Hygiene, Inc.

SUICIDAL IDEATION		
Ask questions 1 and 2. If both are negative, proceed to "Suicidal Behavior" section. If the answer to question 2 is "yes", ask questions 3, 4 and 5. If the answer to question 1 and/or 2 is "yes", complete "Intensity of Ideation" section below.	**Lifetime: Time He/She Felt Most Suicidal**	**Past __ Months**
**1. Wish to be Dead**<br>Subject endorses thoughts about a wish to be dead or not alive anymore, or wish to fall asleep and not wake up.<br>***Have you wished you were dead or wished you could go to sleep and not wake up?***<br><br>If yes, describe:	**Yes** ☐ **No** ☐	**Yes** ☐ **No** ☐
**2. Non-Specific Active Suicidal Thoughts**<br>General non-specific thoughts of wanting to end one's life/commit suicide (e.g., *"I've thought about killing myself"*) without thoughts of ways to kill oneself/associated methods, intent, or plan during the assessment period.<br>***Have you actually had any thoughts of killing yourself?***<br><br>If yes, describe:	**Yes** ☐ **No** ☐	**Yes** ☐ **No** ☐
**3. Active Suicidal Ideation with Any Methods (Not Plan) without Intent to Act**<br>Subject endorses thoughts of suicide and has thought of at least one method during the assessment period. This is different than a specific plan with time, place or method details worked out (e.g. thought of method to kill self but not a specific plan). Includes person who would say, *"I thought about taking an overdose but I never made a specific plan as to when, where or how I would actually do it…and I would never go through with it."*<br>***Have you been thinking about how you might do this?***<br><br>If yes, describe:	**Yes** ☐ **No** ☐	**Yes** ☐ **No** ☐
**4. Active Suicidal Ideation with Some Intent to Act, without Specific Plan**<br>Active suicidal thoughts of killing oneself and subject reports having <u>some intent to act on such thoughts</u>, as opposed to *"I have the thoughts but I definitely will not do anything about them."*<br>***Have you had these thoughts and had some intention of acting on them?***<br><br>If yes, describe:	**Yes** ☐ **No** ☐	**Yes** ☐ **No** ☐
**5. Active Suicidal Ideation with Specific Plan and Intent**<br>Thoughts of killing oneself with details of plan fully or partially worked out and subject has some intent to carry it out.<br>***Have you started to work out or worked out the details of how to kill yourself? Do you intend to carry out this plan?***<br><br>If yes, describe:	**Yes** ☐ **No** ☐	**Yes** ☐ **No** ☐
INTENSITY OF IDEATION		
The following features should be rated with respect to the most severe type of ideation (i.e., 1-5 from above, with 1 being the least severe and 5 being the most severe). Ask about time he/she was feeling the most suicidal.<br><br><u>Lifetime</u> - ***Most Severe Ideation:*** ______ (*Type # (1-5)*) ____________________ (*Description of Ideation*)<br><br><u>Past X Months</u> - ***Most Severe Ideation:*** ______ (*Type # (1-5)*) ____________________ (*Description of Ideation*)	Most Severe	Most Severe
**Frequency**<br>***How many times have you had these thoughts?***<br>(1) Less than once a week (2) Once a week (3) 2-5 times in week (4) Daily or almost daily (5) Many times each day	____	____
**Duration**<br>***When you have the thoughts how long do they last?***<br>(1) Fleeting - few seconds or minutes<br>(2) Less than 1 hour/some of the time<br>(3) 1-4 hours/a lot of time<br>(4) 4-8 hours/most of day<br>(5) More than 8 hours/persistent or continuous	____	____
**Controllability**<br>***Could/can you stop thinking about killing yourself or wanting to die if you want to?***<br>(1) Easily able to control thoughts<br>(2) Can control thoughts with little difficulty<br>(3) Can control thoughts with some difficulty<br>(4) Can control thoughts with a lot of difficulty<br>(5) Unable to control thoughts<br>(0) Does not attempt to control thoughts	____	____
**Deterrents**<br>***Are there things - anyone or anything (e.g., family, religion, pain of death) - that stopped you from wanting to die or acting on thoughts of committing suicide?***<br>(1) Deterrents definitely stopped you from attempting suicide<br>(2) Deterrents probably stopped you<br>(3) Uncertain that deterrents stopped you<br>(4) Deterrents most likely did not stop you<br>(5) Deterrents definitely did not stop you<br>(0) Does not apply	____	____
**Reasons for Ideation**<br>***What sort of reasons did you have for thinking about wanting to die or killing yourself? Was it to end the pain or stop the way you were feeling (in other words you couldn't go on living with this pain or how you were feeling) or was it to get attention, revenge or a reaction from others? Or both?***<br>(1) Completely to get attention, revenge or a reaction from others<br>(2) Mostly to get attention, revenge or a reaction from others<br>(3) Equally to get attention, revenge or a reaction from others and to end/stop the pain<br>(4) Mostly to end or stop the pain (you couldn't go on living with the pain or how you were feeling)<br>(5) Completely to end or stop the pain (you couldn't go on living with the pain or how you were feeling)<br>(0) Does not apply	____	____

© 2008 Research Foundation for Mental Hygiene, Inc. C-SSRS—Baseline/Screening (Version 1/14/09) Page 1 of 2

SUICIDAL BEHAVIOR (Check all that apply, so long as these are separate events; must ask about all types)	Lifetime	Past __ Years
**Actual Attempt:** A potentially self-injurious act committed with at least some wish to die, *as a result of act*. Behavior was in part thought of as method to kill oneself. Intent does not have to be 100%. If there is ***any*** intent/desire to die associated with the act, then it can be considered an actual suicide attempt. ***There does not have to be any injury or harm***, just the potential for injury or harm. If person pulls trigger while gun is in mouth but gun is broken so no injury results, this is considered an attempt. Inferring Intent: Even if an individual denies intent/wish to die, it may be inferred clinically from the behavior or circumstances. For example, a highly lethal act that is clearly not an accident so no other intent but suicide can be inferred (e.g., gunshot to head, jumping from window of a high floor/story). Also, if someone denies intent to die, but they thought that what they did could be lethal, intent may be inferred. ***Have you made a suicide attempt?*** ***Have you done anything to harm yourself?*** ***Have you done anything dangerous where you could have died?*** ***What did you do?*** ***Did you_____ as a way to end your life?*** ***Did you want to die (even a little) when you_____?*** ***Were you trying to end your life when you _____?*** ***Or Did you think it was possible you could have died from_____?*** ***Or did you do it purely for other reasons / without ANY intention of killing yourself (like to relieve stress, feel better, get sympathy, or get something else to happen)?*** (Self-Injurious Behavior without suicidal intent) If yes, describe:	Yes ☐ No ☐ Total # of Attempts ____	Yes ☐ No ☐ Total # of Attempts ____
**Has subject engaged in Non-Suicidal Self-Injurious Behavior?**	Yes ☐ No ☐	Yes ☐ No ☐
**Interrupted Attempt:** When the person is interrupted (by an outside circumstance) from starting the potentially self-injurious act *(if not for that, actual attempt would have occurred)*. Overdose: Person has pills in hand but is stopped from ingesting. Once they ingest any pills, this becomes an attempt rather than an interrupted attempt. Shooting: Person has gun pointed toward self, gun is taken away by someone else, or is somehow prevented from pulling trigger. Once they pull the trigger, even if the gun fails to fire, it is an attempt. Jumping: Person is poised to jump, is grabbed and taken down from ledge. Hanging: Person has noose around neck but has not yet started to hang - is stopped from doing so. ***Has there been a time when you started to do something to end your life but someone or something stopped you before you actually did anything?*** If yes, describe:	Yes ☐ No ☐ Total # of interrupted ____	Yes ☐ No ☐ Total # of interrupted ____
**Aborted Attempt:** When person begins to take steps toward making a suicide attempt, but stops themselves before they actually have engaged in any self-destructive behavior. Examples are similar to interrupted attempts, except that the individual stops him/herself, instead of being stopped by something else. ***Has there been a time when you started to do something to try to end your life but you stopped yourself before you actually did anything?*** If yes, describe:	Yes ☐ No ☐ Total # of aborted ____	Yes ☐ No ☐ Total # of aborted ____
**Preparatory Acts or Behavior:** Acts or preparation towards imminently making a suicide attempt. This can include anything beyond a verbalization or thought, such as assembling a specific method (e.g., buying pills, purchasing a gun) or preparing for one's death by suicide (e.g., giving things away, writing a suicide note). ***Have you taken any steps towards making a suicide attempt or preparing to kill yourself (such as collecting pills, getting a gun, giving valuables away or writing a suicide note)?*** If yes, describe:	Yes ☐ No ☐	Yes ☐ No ☐
**Suicidal Behavior:** Suicidal behavior was present during the assessment period?	Yes ☐ No ☐	Yes ☐ No ☐

Answer for Actual Attempts Only	Most Recent Attempt Date:	Most Lethal Attempt Date:	Initial/First Attempt Date:
**Actual Lethality/Medical Damage:** 0. No physical damage or very minor physical damage (e.g., surface scratches). 1. Minor physical damage (e.g., lethargic speech; first-degree burns; mild bleeding; sprains). 2. Moderate physical damage; medical attention needed (e.g., conscious but sleepy, somewhat responsive; second-degree burns; bleeding of major vessel). 3. Moderately severe physical damage; *medical* hospitalization and likely intensive care required (e.g., comatose with reflexes intact; third-degree burns less than 20% of body; extensive blood loss but can recover; major fractures). 4. Severe physical damage; *medical* hospitalization with intensive care required (e.g., comatose without reflexes; third-degree burns over 20% of body; extensive blood loss with unstable vital signs; major damage to a vital area). 5. Death	*Enter Code* ____	*Enter Code* ____	*Enter Code* ____
**Potential Lethality: Only Answer if Actual Lethality=0** Likely lethality of actual attempt if no medical damage (the following examples, while having no actual medical damage, had potential for very serious lethality: put gun in mouth and pulled the trigger but gun fails to fire so no medical damage; laying on train tracks with oncoming train but pulled away before run over). 0 = Behavior not likely to result in injury 1 = Behavior likely to result in injury but not likely to cause death 2 = Behavior likely to result in death despite available medical care	*Enter Code* ____	*Enter Code* ____	*Enter Code* ____

© 2008 Research Foundation for Mental Hygiene, Inc.

C-SSRS Since Last Visit Form

COLUMBIA-SUICIDE SEVERITY RATING SCALE (C-SSRS)

Since Last Visit

Version 1/14/09

Posner, K.; Brent, D.; Lucas, C.; Gould, M.; Stanley, B.; Brown, G.; Fisher, P.; Zelazny, J.; Burke, A.; Oquendo, M.; Mann, J.

Disclaimer:

This scale is intended to be used by individuals who have received training in its administration. The questions contained in the Columbia-Suicide Severity Rating Scale are suggested probes. Ultimately, the determination of the presence of suicidal ideation or behavior depends on the judgment of the individual administering the scale.

Definitions of behavioral suicidal events in this scale are based on those used in **The Columbia Suicide History Form**, *developed by John Mann, MD and Maria Oquendo, MD, Conte Center for the Neuroscience of Mental Disorders (CCNMD), New York State Psychiatric Institute, 1051 Riverside Drive, New York, NY, 10032. (Oquendo M. A., Halberstam B. & Mann J. J., Risk factors for suicidal behavior: utility and limitations of research instruments. In M.B. First [Ed.]* Standardized Evaluation in Clinical Practice, *pp. 103 -130, 2003.)*

For reprints of the C-SSRS contact Kelly Posner, Ph.D., New York State Psychiatric Institute, 1051 Riverside Drive, New York, New York, 10032; inquiries and training requirements contact posnerk@nyspi.columbia.edu

© 2008 The Research Foundation for Mental Hygiene, Inc.

SUICIDAL IDEATION	
Ask questions 1 and 2. If both are negative, proceed to "Suicidal Behavior" section. If the answer to question 2 is "yes", ask questions 3, 4 and 5. If the answer to question 1 and/or 2 is "yes", complete "Intensity of Ideation" section below.	**Since Last Visit**
**1. Wish to be Dead**<br>Subject endorses thoughts about a wish to be dead or not alive anymore, or wish to fall asleep and not wake up.<br>***Have you wished you were dead or wished you could go to sleep and not wake up?***<br><br>If yes, describe:	Yes ☐ No ☐
**2. Non-Specific Active Suicidal Thoughts**<br>General non-specific thoughts of wanting to end one's life/commit suicide (e.g., *"I've thought about killing myself"*) without thoughts of ways to kill oneself/associated methods, intent, or plan during the assessment period.<br>***Have you actually had any thoughts of killing yourself?***<br><br>If yes, describe:	Yes ☐ No ☐
**3. Active Suicidal Ideation with Any Methods (Not Plan) without Intent to Act**<br>Subject endorses thoughts of suicide and has thought of at least one method during the assessment period. This is different than a specific plan with time, place or method details worked out (e.g., thought of method to kill self but not a specific plan). Includes person who would say, *"I thought about taking an overdose but I never made a specific plan as to when, where or how I would actually do it.....and I would never go through with it".*<br>***Have you been thinking about how you might do this?***<br><br>If yes, describe:	Yes ☐ No ☐
**4. Active Suicidal Ideation with Some Intent to Act, without Specific Plan**<br>Active suicidal thoughts of killing oneself and subject reports having <u>some intent to act on such thoughts</u>, as opposed to *"I have the thoughts but I definitely will not do anything about them".*<br>***Have you had these thoughts and had some intention of acting on them?***<br><br>If yes, describe:	Yes ☐ No ☐
**5. Active Suicidal Ideation with Specific Plan and Intent**<br>Thoughts of killing oneself with details of plan fully or partially worked out and subject has some intent to carry it out.<br>***Have you started to work out or worked out the details of how to kill yourself? Do you intend to carry out this plan?***<br><br>If yes, describe:	Yes ☐ No ☐

INTENSITY OF IDEATION	
The following features should be rated with respect to the most severe type of ideation (i.e.,1-5 from above, with 1 being the least severe and 5 being the most severe).<br>***Most Severe Ideation:*** ________ ________________________<br>*Type # (1-5)* *Description of Ideation*	Most Severe
**Frequency**<br>***How many times have you had these thoughts?***<br>(1) Less than once a week (2) Once a week (3) 2-5 times in week (4) Daily or almost daily (5) Many times each day	___
**Duration**<br>***When you have the thoughts how long do they last?***<br>(1) Fleeting - few seconds or minutes<br>(2) Less than 1 hour/some of the time<br>(3) 1-4 hours/a lot of time<br>(4) 4-8 hours/most of day<br>(5) More than 8 hours/persistent or continuous	___
**Controllability**<br>***Could/can you stop thinking about killing yourself or wanting to die if you want to?***<br>(1) Easily able to control thoughts<br>(2) Can control thoughts with little difficulty<br>(3) Can control thoughts with some difficulty<br>(4) Can control thoughts with a lot of difficulty<br>(5) Unable to control thoughts<br>(0) Does not attempt to control thoughts	___
**Deterrents**<br>***Are there things - anyone or anything (e.g., family, religion, pain of death) - that stopped you from wanting to die or acting on thoughts of committing suicide?***<br>(1) Deterrents definitely stopped you from attempting suicide<br>(2) Deterrents probably stopped you<br>(3) Uncertain that deterrents stopped you<br>(4) Deterrents most likely did not stop you<br>(5) Deterrents definitely did not stop you<br>(0) Does not apply	___
**Reasons for Ideation**<br>***What sort of reasons did you have for thinking about wanting to die or killing yourself? Was it to end the pain or stop the way you were feeling (in other words you couldn't go on living with this pain or how you were feeling) or was it to get attention, revenge or a reaction from others? Or both?***<br>(1) Completely to get attention, revenge or a reaction from others<br>(2) Mostly to get attention, revenge or a reaction from others<br>(3) Equally to get attention, revenge or a reaction from others and to end/stop the pain<br>(4) Mostly to end or stop the pain (you couldn't go on living with the pain or how you were feeling)<br>(5) Completely to end or stop the pain (you couldn't go on living with the pain or how you were feeling)<br>(0) Does not apply	___

© 2008 Research Foundation for Mental Hygiene, Inc.

SUICIDAL BEHAVIOR<br>*(Check all that apply, so long as these are separate events; must ask about all types)*	Since Last Visit
**Actual Attempt:**<br>A potentially self-injurious act committed with at least some wish to die, *as a result of act*. Behavior was in part thought of as method to kill oneself. Intent does not have to be 100%. If there is ***any*** intent/desire to die associated with the act, then it can be considered an actual suicide attempt. ***There does not have to be any injury or harm***, just the potential for injury or harm. If person pulls trigger while gun is in mouth but gun is broken so no injury results, this is considered an attempt.<br>Inferring Intent: Even if an individual denies intent/wish to die, it may be inferred clinically from the behavior or circumstances. For example, a highly lethal act that is clearly not an accident so no other intent but suicide can be inferred (e.g., gunshot to head, jumping from window of a high floor/story). Also, if someone denies intent to die, but they thought that what they did could be lethal, intent may be inferred.<br>***Have you made a suicide attempt?***<br>***Have you done anything to harm yourself?***<br>***Have you done anything dangerous where you could have died?***<br>***What did you do?***<br>***Did you______as a way to end your life?***<br>***Did you want to die (even a little) when you_____?***<br>***Were you trying to end your life when you_____?***<br>***Or Did you think it was possible you could have died from_____?***<br>***Or did you do it purely for other reasons / without ANY intention of killing yourself (like to relieve stress, feel better, get sympathy, or get something else to happen)?*** (Self-Injurious Behavior without suicidal intent)<br>If yes, describe:	Yes ☐ No ☐<br>Total # of Attempts<br>____
**Has subject engaged in Non-Suicidal Self-Injurious Behavior?**	Yes ☐ No ☐
**Interrupted Attempt:**<br>When the person is interrupted (by an outside circumstance) from starting the potentially self-injurious act *(if not for that, actual attempt would have occurred)*.<br>Overdose: Person has pills in hand but is stopped from ingesting. Once they ingest any pills, this becomes an attempt rather than an interrupted attempt. Shooting: Person has gun pointed toward self, gun is taken away by someone else, or is somehow prevented from pulling trigger. Once they pull the trigger, even if the gun fails to fire, it is an attempt. Jumping: Person is poised to jump, is grabbed and taken down from ledge. Hanging: Person has noose around neck but has not yet started to hang - is stopped from doing so.<br>***Has there been a time when you started to do something to end your life but someone or something stopped you before you actually did anything?***<br>If yes, describe:	Yes ☐ No ☐<br>Total # of interrupted<br>____
**Aborted Attempt:**<br>When person begins to take steps toward making a suicide attempt, but stops themselves before they actually have engaged in any self-destructive behavior. Examples are similar to interrupted attempts, except that the individual stops him/herself, instead of being stopped by something else.<br>***Has there been a time when you started to do something to try to end your life but you stopped yourself before you actually did anything?***<br>If yes, describe:	Yes ☐ No ☐<br>Total # of aborted<br>____
**Preparatory Acts or Behavior:**<br>Acts or preparation towards imminently making a suicide attempt. This can include anything beyond a verbalization or thought, such as assembling a specific method (e.g., buying pills, purchasing a gun) or preparing for one's death by suicide (e.g., giving things away, writing a suicide note).<br>***Have you taken any steps towards making a suicide attempt or preparing to kill yourself (such as collecting pills, getting a gun, giving valuables away or writing a suicide note)?***<br>If yes, describe:	Yes ☐ No ☐
**Suicidal Behavior:**<br>Suicidal behavior was present during the assessment period?	Yes ☐ No ☐
**Suicide:**	Yes ☐ No ☐
Answer for Actual Attempts Only	Most Lethal Attempt Date:
**Actual Lethality/Medical Damage:**<br>0. No physical damage or very minor physical damage (e.g., surface scratches).<br>1. Minor physical damage (e.g., lethargic speech; first-degree burns; mild bleeding; sprains).<br>2. Moderate physical damage; medical attention needed (e.g., conscious but sleepy, somewhat responsive; second-degree burns; bleeding of major vessel).<br>3. Moderately severe physical damage; *medical* hospitalization and likely intensive care required (e.g., comatose with reflexes intact; third-degree burns less than 20% of body; extensive blood loss but can recover; major fractures).<br>4. Severe physical damage; *medical* hospitalization with intensive care required (e.g., comatose without reflexes; third-degree burns over 20% of body; extensive blood loss with unstable vital signs; major damage to a vital area).<br>5. Death	*Enter Code*<br>____
**Potential Lethality: Only Answer if Actual Lethality=0**<br>Likely lethality of actual attempt if no medical damage (the following examples, while having no actual medical damage, had potential for very serious lethality: put gun in mouth and pulled the trigger but gun fails to fire so no medical damage; laying on train tracks with oncoming train but pulled away before run over).<br>0 = Behavior not likely to result in injury<br>1 = Behavior likely to result in injury but not likely to cause death<br>2 = Behavior likely to result in death despite available medical care	*Enter Code*<br>____

© 2008 Research Foundation for Mental Hygiene, Inc. C-SSRS—Since Last Visit (Version 1/14/09) Page 2 of 2

Appendix C7: Grip Strength

Grip strength will be evaluated using a dynamometer, as a measure of the severity of the neurological effects of snakebite. Grip strength should be measured in the dominant hand, or in the non-dominant hand if the bite occurred on dominant hand.

The grip strength assessment is an objective measurement of hand function. The subject is asked to grip a dynamometer and squeeze with maximal force. The measurement is repeated for a total of 3 trials and the greatest value is recorded. Completion takes approximately 1 minute.

References:

Jones LA. The assessment of hand function: a critical review of techniques. J Hand Surg 1989; 14A:221-8.

Peters MJH, van Nes SI, Vanhoutte EK Bakkers M, van Doorn PA, Merkies ISJ, Faber CG, PeriNomS Study group. Revised normative values for grip strength with the Jamar dynamometer. J Periph Nervous Sys 2011; 16(1): 47-50.

Instrument: A standard dynamometer, such as the Jamar Hand Dynamometer, will be used according to the manufacturer's instructions.

Appendix C8: Modes of Mechanical Ventilation

Record the following in all endotracheally intubated patients. Modes of Ventilation and times where modes and settings changed will be collected in the CRF.

- Mechanical ventilation modes and ventilator settings
  - Control mode ventilation
    - Volume control
    - Pressure control
    - Assist control
    - Pressure-regulated volume control
  - Weaning mode ventilation
    - Synchronized intermittent mandatory ventilation
    - Pressure support
    - Adaptive support ventilation
    - Airway pressure release ventilation
    - T piece
  - Other
    (Since novel modes such as adaptive support ventilation and airway pressure release ventilation can be used during initiation, maintenance, or weaning phases of mechanical ventilation, clear documentation is warranted)
- Record NIF if collected per institutional practice

Appendix C9: Head-Lift Duration

The subject is asked to raise their head approximately 8 cm off the bed (enough to see clear separation from the head and the mattress). The duration of time that the patient can keep their head off the bed is measured for a maximum duration of 5 seconds (e.g. 1, 2, 3, 4, 5 seconds or if using timing device e.g. 4.25 seconds). Please note the duration of Head-Lift in seconds. Record up to 2 decimal places if using an electronic timing device (e.g. stop-watch). The measurement is repeated for a total of 3 trials and the greatest value up to 5 seconds is recorded. If the patient achieves 5 second head lift on the first attempt, then the task is completed. Completion takes approximately 1 minute.

References:

Heier T, Caldwell JE, Feiner JR, Liu L, Ward T, Wright PM. Relationship between normalized adductor pollicis train-of-four ratio and manifestations of residual neuromuscular block: a study using acceleromyography during near steady-state concentrations of mivacurium. *Anesthesiology.* 2010;113:825–832.

Lewin MR, Bickler P, Heier T, Feiner J, Montauk L, Mensh B. Reversal of experimental paralysis in a human by intranasal neostigmine aerosol suggests a novel approach to the early treatment of neurotoxic envenomation. *Clin Case Rep*. 2013;1(1):7-15. doi:10.1002/ccr3.3

Appendix C10: Analgesic Use

This analgesic use assessment is derived from data in the patient's medication history as collected through recording of concomitant medication usage, supplemented by focused questions if needed.

For each assessment time point, the patient's current analgesic use will be characterized by the clinical research team based on the strongest category of analgesic taken to treat snakebite-related pain, as follows:

Analgesic use category	Ordinal scale value
No analgesic	1
Nonprescription analgesic only	2
Prescription analgesic, non-opioid	3
Opioid analgesic (including tramadol, and opioid combination products)	4

The result is a 4-point ordinal scale with a range of possible values. Collection of these data should require less than 1 minute in addition to the time for overall medication use.

Reference: None

Instrument: None

APPENDIX D COVID-19 PROTOCOL DEVIATIONS

The impact of COVID-19 on this clinical study and study subjects will be extensively described in the SAP and in the CSR. The following aspects will be summarized when protocol deviations are due to COVID-19 infection or restriction:

- Changes to treatment dispensation
- Changes to treatment administration
- Changes to visit windows to accommodate delays for some assessments
- Missing efficacy endpoints
- Missing visits

APPENDIX E REGULATIONS AND GOOD CLINICAL PRACTICE GUIDELINES

1. Regulations

Refer to the following United States CFR:

- FDA Regulations 21 CFR, Parts 50.20 – 50.27
  Subpart B – Informed Consent of Human Subjects
- FDA Regulations 21 CFR, Parts 56.107 – 56.115
  Part 56 – Institutional Review Boards
  Subpart B – Organization and Personnel
  Subpart C – IRB Functions and Operations
  Subpart D – Records and Reports
- FDA Regulations 21 CFR, Parts 312.50 – 312.70
  Subpart D – Responsibilities of Sponsors and Investigators

2. Good Clinical Practice Guidelines

ICH GCP guidelines can be found at the following URL:

https://ich.org/page/efficacy-guidelines
